# The contingent impact of wind farms on game mammal density demonstrated in a large-scale analysis of hunting bag data in Poland

**DOI:** 10.1038/s41598-024-76999-2

**Published:** 2024-10-25

**Authors:** Daniel Klich, Joanna Kawka, Rafał Łopucki, Zuzanna Kulis, Gigorij Yanuta, Maciej Budny

**Affiliations:** 1 Department of Animal Genetics and Conservation, Warsaw University of Life Sciences (SGGW), Ciszewskiego 8, 02‑786 Warsaw, Poland; 2 Department of Biomedicine and Environmental Research, The John Paul II Catholic University of Lublin, Konstantynów 1J, 20-708 Lublin, Poland; 3 Polish Hunting Association, Research Station, Sokolnicza 12, 64-020 Czempiń, Poland

**Keywords:** Ungulates, Herbivores, Carnivores, Roe deer, Wild boar, Renewables infrastructure, Environmental impact, Population dynamics

## Abstract

Wind farms are still developing dynamically worldwide, with promising prospects for further growth. Therefore, the assessment of their impact on animals has been carried out. So far, few studies have been conducted on game mammals, and their results are divergent. Previous studies on the impact of wind farms on game species were typically based on regional research covering one or, at most, several wind farms. In this study, we aimed to verify the effect of wind farms on the density of game mammals through a large-scale analysis at the country level, using lowland Poland as an example. The study was based on hunting bag data from open-field hunting districts. It covered seven game species: roe deer (*Capreolus capreolus*), wild boar (*Sus scrofa*), red fox (*Vulpes vulpes*), raccoon dog (*Nyctereutes procyonoides*), European badger (*Meles meles*), European polecat (*Mustela putorius*), and European hare (*Lepus europaeus*). We used Corine Land Cover to account for differences in land cover and the area covered by wind farms in generalized linear mixed models. The study showed that in agricultural landscapes, mainly herbivorous species of game mammals were related to land cover types. These species tend to exhibit higher densities in agricultural areas containing more natural landscape features. Conversely, mesocarnivores are primarily driven by the abundance of prey with little to no observable effects from land cover types. Only roe deer and wild boar presented lower densities with an increase in the area covered by wind farms (for roe deer: estimate: − 0.05, 95% CI: − 0.1–0.0; for wild boar: estimate: − 0.03, 95% CI: − 0.11–0.05), while no effect was observed for mesocarnivores or European hare. The underlying reasons for these relationships remain unclear and require more specific studies. The uncertainty regarding the cause of the observed effects did not allow for a large-scale assessment of the impact of further wind energy development on the studied game mammals.

**Supplementary Information:**

The online version contains supplementary material available at 10.1038/s41598-024-76999-2.

## Introduction

Wind farms are still developing dynamically worldwide, with promising prospects for further growth^1–3^. This technology is already common, and its effects are increasingly being verified for environmental, economic, and political reasons^4–9^.

The impact of wind farms on animals, including birds, bats, and mammals, has been assessed^10,11^. However, the effects on game animals have been considered less significant^12^. This is because these species, in addition to their economic and trophy value, have more abundant populations and demonstrate high adaptability to changing environments (e.g.^13,14^). Nevertheless, theoretical and experimental studies have suggested a potential negative impact of wind turbines on mammals. For example, Santos et al.^15^ simulated that a decrease in species diversity may be connected to the presence of wind farms. According to Koschinski et al.^16^ and Madsen et al.^17^, a behavioral response of mammals to wind turbine noise should be expected. Subsequent studies confirmed the impact of wind farms on selected mammal species, including game species, but the results of different studies were only sometimes consistent^18^. For many years, there has been a lack of sufficiently intensive field studies to confirm these hypotheses. This aspect seems important because game species are under regular hunting pressure, which can produce a more robust response to human pressure than to natural threats^19^, and an additional, evolutionarily new environmental factor may become significant (e.g.^20–22^).

So far, few studies have been conducted on game mammals, and their results are contradictory. This also applies to ungulate species. For example, wind farm development did not affect the home range and diet of Rocky Mountain elk (*Cervus elaphus*)^23^. Flydal et al.^24^ showed no significant effect of wind farms on the behavior of semi-domesticated reindeer (*Rangifer tarandus*). According to Taylor et al.^25^, wind energy infrastructure did not influence the winter survival of pronghorn (*Antilocapra americana*), though it was suggested that larger wind farms could affect survival. Milligan et al.^26^ also reported a lack of consistent adverse effects on pronghorn. For reindeer, both avoidance and lack of influence of wind farms have been observed^27,28^. However, wind farms were found to reduce the use of movement corridors and grazing habitats, increasing the fragmentation of reindeer calving ranges^29^. Skarin et al.^30^ found that the operation phase of wind farms had a stronger adverse impact on reindeer habitat selection than the construction phase. For roe deer (*Capreolus capreolus*), Łopucki et al.^31^ showed a tendency to avoid wind farm interiors and direct proximity to turbines, and Klich et al.^32^ reported an increase in stress levels in roe deer inhabiting larger wind farms. In forest habitats, however, no effect of wind turbines on the presence of roe deer (*Capreolus capreolus*), wild boar (*Sus scrofa*), or water deer (*Hydropotes inermis*) has been shown^33^.

Contradictory study findings have also been obtained for other game mammals. In a study by Łopucki et al.^31^, red foxes (*Vulpes vulpes*) showed lower track density in wind farm areas than control areas; still, no adverse effect was observed regarding the proximity to individual turbines. The authors explained this effect by the probable lower hunting efficiency of red foxes within the wind farm due to turbine noise (which blurs the sounds of their prey) rather than avoidance of the infrastructure itself. Similarly, heavily poached golden jackals (*Canis aureus*) in India exhibited significantly lower occupancy in wind farm areas^34^. American martens (*Martes americana*) showed a decline in spatial use of wind farm areas^35^, and Sirén speculated that there is a potential to reduce the viability of local marten populations^36^.

In contrast, in forest habitats with wind farms, Kim et al.^33^ showed no effect on badgers (*Meles leucurus*) and raccoon dogs (*Nyctereutes procyonoides*) but reported a negative impact on martens (*Martes flavigula*). No effect of wind turbines on occupancy was observed for black-naped hare (*Lepus nigricollis*)s in India^34^, but lower track density was observed for European hare (*Lepus europaeus*) within wind farms and near turbines^31^. Rabin et al.^37^ showed increased vigilance and anti-predator behavior in California ground squirrels (*Otospermophilus beecheyi*) exposed to wind turbine noise.

All the previously mentioned studies on the impact of wind farms on game species were local and covered sites from one to a few wind farms. However, large-scale studies need to consider the diversity of species’ responses in various environmental and population contexts. According to Schöll and Nopp-Mayr^18^, different response types to wind farms within one species may be related to local, including demographic, differences. Large-scale studies, although usually provide low-resolution information, would give a more general understanding of the issue. Therefore, they can provide a basis for local studies and prevent the extrapolation of observations from a single wind farm to a national or continental scale.

Given this context, we aimed to assess the impact of wind farms on the abundance of a small group of common game mammals through a large-scale analysis at the country level, using the lowland Poland area as an example. For this purpose, we utilized hunting bag data, commonly employed as a proxy in studies on game species, to assess abundance and population dynamics^38–41^ and environmental impact studies^42–44^. In this study, we hypothesized that game mammals would exhibit lower abundance in areas with a higher density of wind farms. We anticipated that these animals would be affected by a combination of environmental factors, like new infrastructure, hunting, and other human pressures. Recognizing that habitat structure can influence animal density, we included basic land cover types in our analysis.

## Methods

The study was based on game management units - hunting districts, the smallest administrative units in Poland for which detailed and publicly available data on game animals are provided. These districts are leased by hunting clubs within the Polish Hunting Association. Each hunting club is required to prepare an annual hunting report, which includes, among other information, the total annual harvest per species in each leased hunting district. Each hunting club is required to assess the game numbers and report by 10 March each year. The assessment of game numbers is verified by the forest district managers of the forest districts where the hunting district is located. Various methods are used for the estimation of animal numbers. Still, the most common is the hunter estimation method, which is based on year-round observations of animals and gives the most accurate results in open areas^45,46^. In addition, other methods are sometimes used, such as strip counting or winter tracking. In the case of predators, spotlight count is also used. However, as Hušek et al.^47^ showed, these methods give comparable results. For this study, we obtained data on game mammals in Poland for the 2020/2021 hunting season from the resources of the Research Station of the Polish Hunting Association in Czempiń (https://czempin.pzlow.pl/). The data on game animals included the number of animals harvested during the hunting season per 100 hectares of the hunting district area (Table S1). The mean area of the analyzed hunting districts was 6,127 hectares.

The climate in Poland is temperate, determined by two – polar maritime (oceanic) and continental – air masses. Average monthly temperatures range from - 4–-1 °C in cold months to 17–19 °C in warm months. Frosty days (Tmax < 0 °C) are registered from November through to March, though with the greatest number in January (11–14 days in the month). Annual precipitation in central and northern Poland equals 500–700 mm, with an increase to the south (780 mm in the belt of uplands and 1100 mm in the mountains)^48,49^. Poland is a country with a varied topography with the seacoast, lowlands, uplands, and mountains. Lowlands (regions of up to 300 m ASL) dominate in Poland, covering nearly 92% of the country^49^. In some parts of Poland, there are numerous lake regions located mainly in northern Poland. The uplands, areas between 300 and 500 m ASL, constitute 5.6% of Poland’s area. They are located in Poland’s southern and eastern parts, and their landscape is more diverse than in the lowlands. The mountains are located in southern Poland, by the borders with the Czech Republic and Slovakia, and reach altitudes from 500 to almost 2500 m ASL. They present a distinct type of landscape and significant altitude differences^49^. About 62% of the country’s total area is occupied by agricultural land (including arable land - about 45%, meadows and pastures − 9% and others), mainly located in the lowlands^50^ Semi-natural and forest areas cover about 30% of Poland, and coniferous forests are the dominant forest type. Higher land cover diversity is observed in eastern and southern Poland^50,51^.

**Fig. 1:**
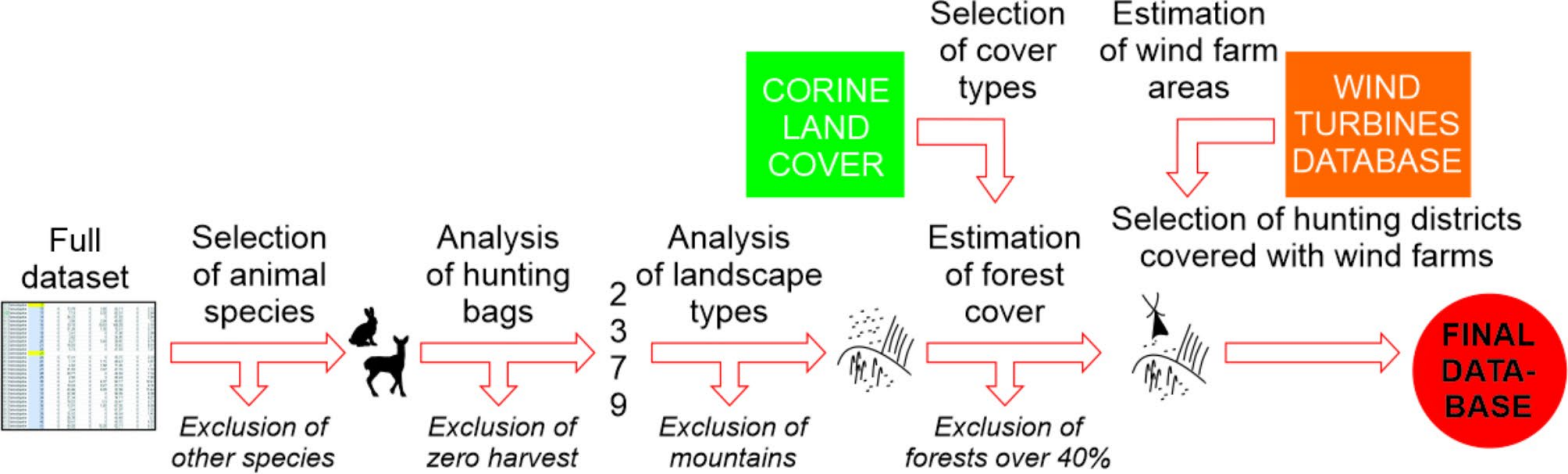
Diagram showing the data elaboration and selection order.

The data were pre-processed to select species for analysis. We selected species adaptable to various environments with a substantial harvest to ensure an adequate number of observations for analysis. These species needed to be present and hunted throughout lowland Poland. Among ungulates, we chose species dwelling in agricultural landscapes: roe deer (*Capreolus capreolus*) and wild boar (*Sus scrofa*). Moreover, we included the following five species: red fox (*Vulpes vulpes*), raccoon dog (*Nyctereutes procyonoides*), European badger (*Meles meles*), European polecat (*Mustela putorius*), and European hare (*Lepus europaeus*). We only considered hunting districts where at least one specimen was harvested for each species. Instances with zero harvests were excluded to prevent potential biases in the database, which could result from unreported numbers, species absence in the hunting plan, or temporary hunting suspensions in specific districts. Additionally, to minimize landscape variations that could influence harvesting levels due to local conditions, hunting districts in the Sudetes and Carpathian Mountain ranges in southern Poland were excluded from the analysis (Fig. 1). The mountains occupy low parts of Poland, and are characterized by a completely different landscape, climate conditions and land cover.

**Fig. 2:**
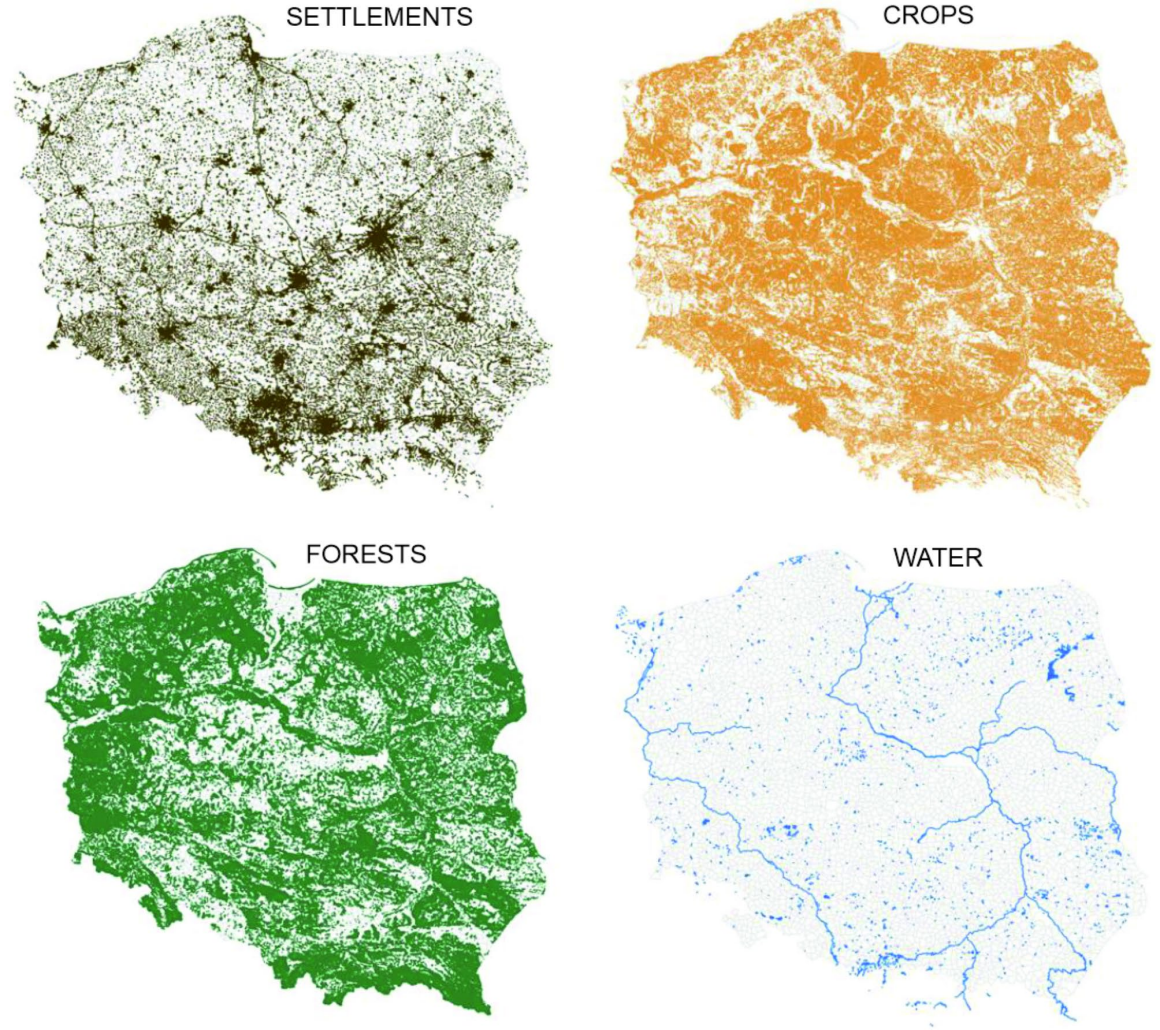
Selected land cover types for analysis: SETTLEMENTS (CLC classes: 1.1, 1.2 and 1.3), CROPS (CLC classes: 2.1 and 2.2), FORESTS (CLC classes: 3.1 and 3.2) and WATER (CLC classes: 4.1 and 5.1), source: https://clc.gios.gov.pl/.

Next, we assigned basic land cover types for each hunting district based on Corine Land Cover (CLC 2018, https://clc.gios.gov.pl/). We applied the vector CLC layer (polygons) to the boundaries of hunting districts (vector layer-polygons) and calculated the percentage of forest class (CLC class 3.1) using QGIS 3.22. Based on the forest class, we selected open-field hunting districts where the forest class was at most 40% of the district’s area. This selection was made because onshore wind farms in Poland are typically located in agricultural landscapes. Additionally, we calculated the percentage of four main land cover types in each hunting district using CLC layer, where selected Corine Land Cover classes were added to create four main cover types (Fig. 2):

- SETTLEMENTS (including continuous urban fabric, discontinuous urban fabric, industrial areas, transport units, and construction sites; CLC classes: 1.1, 1.2, and 1.3),
- CROPS (mainly arable land and permanent crops; CLC classes: 2.1 and 2.2),
- FORESTS (areas covered with natural woody vegetation, including forests and shrub vegetation; CLC classes: 3.1 and 3.2),
- WATER (including wetlands and water bodies; CLC classes: 4.1 and 5.1).

**Fig. 3:**
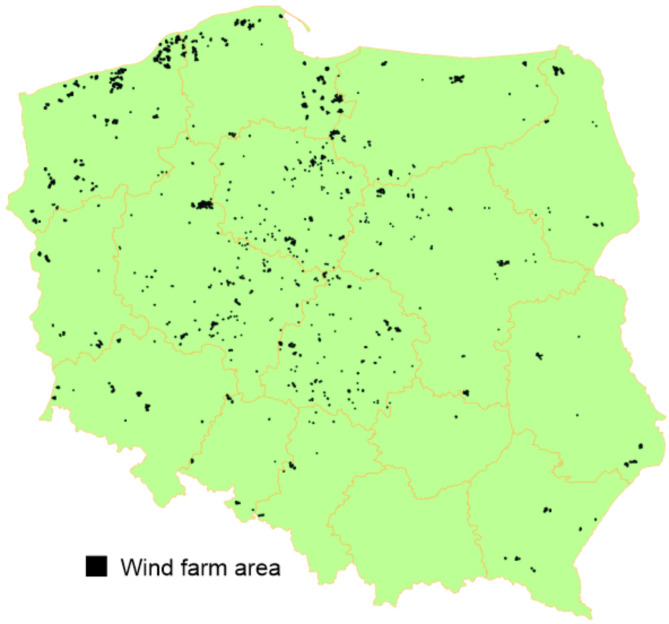
Distribution of wind farm areas in Poland (wind farm areas were calculated with 700 ha buffers for turbines).

Next, we assessed the potential spatial impact of wind farms in Poland using the list of registered wind turbines available in the Polish Aviation Obstacles database (https://caa-pl.maps.arcgis.com/apps/webappviewer/index.html?id=252d2be2e6104adcb9be8201660a05b3.pdf). We created a point layer based on the geographical coordinates of operating wind turbines for 2021. For each turbine point, we generated a buffer with a radius of 700 m. These buffers were combined to create a vector layer (polygon) representing the extent of coverage for each wind farm. Using this wind farm cover layer, we calculated the percentage of each hunting district’s area that a wind farm covered. The selection of a 700-meter buffer was based on existing research indicating this distance as the impact radius of wind turbines on animals^31^ (Fig. 3). For further analysis, we included all hunting districts where wind farms were present (Fig. 1).

Finally, for the statistical analysis, we obtained 478 hunting districts for roe deer, 469 hunting districts for wild boar, 483 hunting districts for red fox, 463 hunting districts for raccoon dog, 464 hunting districts for European badger, 335 hunting districts for European polecat and 154 hunting districts for European hare (Table 1). Harvest range (ind/100 ha) for each species was as follows: roe deer: 0.6–34, wild boar: 0.1–43.5, red fox: 1.0–60.3, raccoon dog: 0.1–17.9, European badger: 0.1–5.6, European polecat: 0.1–11.2, and European hare: 0.4–22.5. Depending on the species, hunting districts were covered in various percentages by selected cover types. CROPS were the main cover type and constituted 37.1 to 99.2 or 99.4% of the hunting district area, but higher minimal coverage was in the case of the European hares (55.3%). FORESTS constituted 0.0 to 39.5% for European hare and 0.0 to 43.6% for all other species. SETTLEMENTS covered from 0.0 to 22.4% for European polecat, 0.0 to 27.9 for raccoon dogs, European badgers, and European hare, and up to 50.3% for other species. WATER covered 0.0 to 0.1% for European hare and 0.0 to 43.6% for all other analyzed species. Wind farm area (with a 700 m buffer) covered from 0.0 to 36.3% of hunting districts for European hares and from 0.0 to 58.8% of hunting districts for all other studied species (Table 1).

**Table 1:** Main features of the studied hunting grounds for each species (range given for percentages). (*0.0 represent percentage of wind farm cover lower than 0.05 but always higher than 0).

	Districts (*n*)	Settlements (%)	Crops (%)	Forests (%)	Water (%)	Wind farm (%)*
Roe deer	478	0.0–50.3	37.1–99.4	0.0–43.6	0.0–27.3	0.0–58.6
Wild boar	469	0.0–50.3	37.1–99.4	0.0–43.6	0.0–27.3	0.0–58.6
Red fox	483	0.0–50.3	37.1–99.4	0.0–43.6	0.0–27.3	0.0–58.8
Racoon dog	463	0.0–27.9	37.1–99.2	0.0–43.6	0.0–27.3	0.0–58.8
European badger	464	0.0–27.9	37.1–99.2	0.0–43.6	0.0–27.3	0.0–58.8
European polecat	335	0.0–22.4	37.1–99.2	0.0–43.6	0.0–27.3	0.0–58.8
European hare	154	0.0–27.9	55.3–99.2	0.0–39.5	0.0–0.1	0.0–36.3

### Statistical analysis

Before the final statistical analyses, we verified if the hunting bags were correlated with the density of animals estimated for hunting districts. Therefore, we used Pearson’s correlation coefficient for each analyzed species separately. For all species, the correlation was moderate and significant; for roe deer: *r* = 0.527, *p* < 0.001, for wild boar: *r* = 0.591, *p* < 0.001, for red fox: *r* = 0.351, *p* < 0.001, for raccoon dog: *r* = 0.726, *p* < 0.001, for European badger: *r* = 0.518, *p* < 0.001, for European polecat: *r* = 0.523, *p* < 0.001 and for European hare: *r* = 0.464, *p* < 0.001 (Figure S1). We also analyzed the relation of hunting bags and animal density using a generalized linear mixed model with gamma distribution and log link function. We included the ID of voivodeships (administrative regions in Poland) as a random factor to account for variations in game mammal populations across different regions. All relations were significant (Table S2).

To evaluate the impact of wind farms and land cover types on the abundance of game mammals, we employed a generalized linear mixed model (GLMM) in Lme4 package in R (https://cran.r-project.org/web/packages/lme4/index.html)^52^. GLMMs are suitable for handling correlations among multiple observations from unique sampling units. Therefore, we included the ID of voivodeships (administrative regions in Poland) as a random factor to account for variations in game mammal populations across different regions. We used a gamma distribution with a log link function, with model parameters estimated through maximum likelihood. Seven models were constructed, each focusing on one of the seven studied species. The dependent variable in each model was the number of harvested individuals of a given species per 100 hectares of hunting district area. In all models, we incorporated five explanatory variables expressed as percentages of the hunting district area: SETTLEMENTS, CROPS, FORESTS, WATER, and WIND FARM. Summarized data for all variables are presented in Table 1, with detailed data provided in Table S1.

Model selection was based on the corrected Akaike information criterion (AICc)^53^. The best-fit models were identified by the lowest AICc score, and models with a ΔAICc < 2 were considered plausible following Burnham and Anderson’s guidelines. We explored all possible combinations of explanatory variables and compared them to a null (intercept-only) model. Final model parameters and 95% confidence intervals (95% CI) were estimated using model averaging across all models with ΔAICc < 2, implemented with the ‘modavg’ package (https://cran.r-project.org/web/packages/AICcmodavg/index.html)^54^.

## Results

**Fig. 4:**
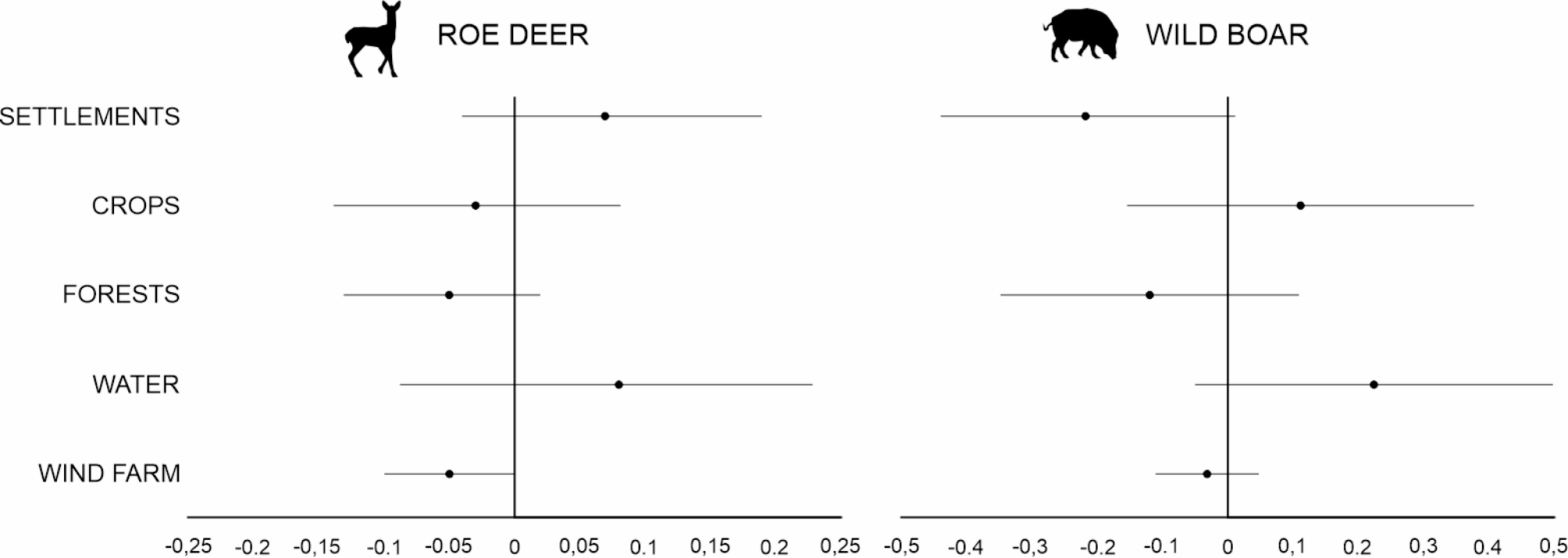
Parameter estimates of roe deer and wild boar response to land cover types and wind farm. Model averaged estimates and confidence intervals are taken from top models within ΔAICc < 2 (variables: see methods). The best random model (with random intercept and random slope) for roe deer abundance included FORESTS and WIND FARM and for wild boar SETTLEMENTS and FORESTS (Table S3).

The final AICc model set for *roe deer* included seven models with a ΔAICc < 2 (Table S3). The top model identified included FORESTS and WIND FARM. The hunting bag for roe deer increased with SETTLEMENTS and WATER. Still, it decreased with CROPS, FORESTS, and WIND FARM (Fig. 4). The variable WIND FARM appeared in the roe deer hunting bag as it was retained in all models, with an estimate of -0.05 and its 95% CI in the range of -0.1–0.0. However, the models showed poor fit, as indicated by a ΔAICc of 2.71 between the null and top models (Table S3). The direction of the effect for other variables was inconclusive, as their 95% CIs distinctly overlapped 0.

Similarly, seven models were included in the AICc model set for *wild boar*, but the top model included only SETTLEMENTS and FORESTS. The hunting bag for wild boar increased with CROPS and WATER but decreased with other cover types and WIND FARM (Fig. 4). For all variables, the direction of the effect was inconclusive, as their 95% CIs overlapped 0 (estimate: -0.03, 95%CI: -0.11–0.05). However, the negative effect of SETTLEMENTS was most pronounced, as the overlap was minimal. Despite the notable ΔAICc of 16.25 between the top model and the null model, WIND FARM was included in the model ranked sixth (Table S3).

**Fig. 5:**
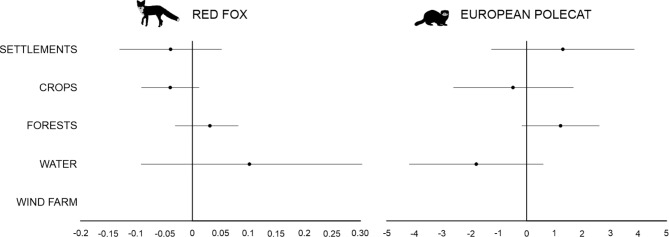
Parameter estimates of red fox and European polecat response to land cover types and wind farm. Model averaged estimates and confidence intervals are taken from top models within ΔAICc < 2 (variables: see methods). The best random model (with random intercept and random slope) for red fox abundance included only CROPS, while for European polecat the best model included CROPS and WATER (Table S3).

The AICc model set for *red fox* included six models with a ΔAICc < 2 (Table S3), but the top model included only CROPS. The hunting bag for red fox increased with FORESTS and WATER, and decreased with SETTLEMENTS and CROPS. WIND FARM was not included in the final AICc model set (Table S3). However, for all variables, the direction of the effect was inconclusive as the 95% CI overlapped 0. The negative effect of CROPS appeared most pronounced, with the least overlap (Fig. 5). Similar to roe deer, the models for red fox seemed poorly fitted, with a ΔAICc of 2.10 between the null model and the top model (Table S3).

The effects were inconclusive for *raccoon dog* and *European badger* as the null models fell within ΔAICc < 2, with the second-ranked model for raccoon dog and the highest-ranked model for European badger (Table S3).

The AICc model set for *European polecat* included seven models with a ΔAICc < 2, but the top model included CROPS and WATER (Table S3). The hunting bag for European polecat increased with SETTLEMENTS and FORESTS, but decreased with CROPS and WATER (Fig. 5). WIND FARM was not included in the final AICc model set (Table S3). Similarly, for all variables, the direction of the effect was inconclusive as the 95% CI overlapped 0. However, the negative impact of FORESTS was most pronounced, with the lowest overlap (Fig. 5).

**Fig. 6:**
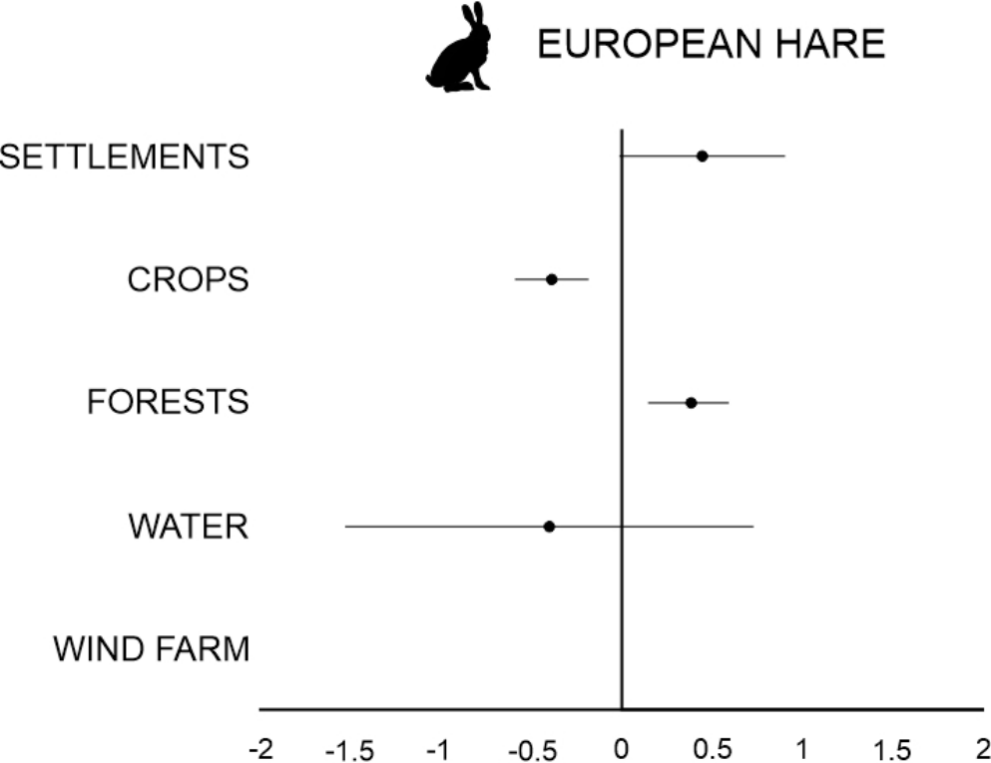
Parameter estimates of European hare response to land cover types and wind farm. Model averaged estimates and confidence intervals are taken from top models within ΔAICc < 2 (variables: see methods). The best random model (with random intercept and random slope) for European hare abundance included only CROPS (Table S3).

The final AICc model set for *European hare* included only three models with a ΔAICc < 2, and the top model included only CROPS (Table S3). The hunting bag for European hare increased with SETTLEMENTS and FORESTS, but decreased with CROPS and WATER. WIND FARM was not included in the final AICc model set (Table S3). For WATER, the direction of the effect was inconclusive as the 95% CI overlapped 0. However, the effects were clear for SETTLEMENTS, CROPS, and FORESTS (Fig. 6).

## Discussion

In our study, we utilized official hunting bag data from the Research Station of the Polish Hunting Association in Czempiń. Such data are commonly used in various studies on game species^38–43^. We carefully pre-processed the data to minimize factors that could impact our findings’ reliability.

In our study, we observed several intriguing relationships that suggest significant directions for further research into the interactions between game species and the contemporary environment under various human pressures. It’s important to note that the context of open-field hunting districts situated within agricultural landscapes may not always align with general trends observed for the analyzed animal species. Most animal species did not exhibit clear relationships concerning land cover types, which is consistent with previous findings indicating that species density is influenced by many factors (e.g.^55–61^), including local variables that are challenging to capture. Therefore, we focus on discussing the most prominent relationships—those showing clearer trends—while omitting others that likely reflect localized patterns and necessitate a more detailed approach not pursued in this study.

### Effects of land cover types

Roe deer did not exhibit specific relationships with land cover types in the agricultural landscape of open-field hunting districts. However, the results indicated a more pronounced negative relationship with forests. Although woodlands are crucial for roe deer as safe habitats during both daytime and the hunting season^62,63^, this species shows higher abundance in agriculture-dominated landscapes^61,64^ and a minimal amount of forest is sufficient for roe deer to thrive in human-dominated landscapes^65^.

Similarly, wild boar showed ambiguous relationships with land cover types. The most notable negative effect was observed for settlements. This finding is unsurprising given that wild boar has been heavily hunted for years, particularly due to African swine fever (ASF). Consequently, the population of this species has significantly declined, leading to further ecological consequences^66–68^. Wild boar tend to avoid areas where obtaining quality food involves a high risk of being killed^69^. Therefore, increased hunting pressure leads to an expectation that wild boar will avoid settlements associated with human presence, even in agricultural landscapes.

Mesocarnivores exhibited even more ambiguous relationships compared to ungulates. No clear explanation for their abundance could be determined for two of the four species (raccoon dog and European badger). Mesocarnivores depend on prey abundance rather than specific habitat types (e.g.^70–72^). Moreover, most of the analyzed species, such as red fox, raccoon dog, and European badger, are found in various habitat types, including those typical of agricultural landscapes in central Europe^73–76^. In the case of the European polecat, there was a clear positive relationship with forests. The European polecat is strongly associated with forest habitats^77,78^.

The abundance of European hare showed a positive relationship with settlements and forests and a negative relationship with crops. Previous studies have consistently demonstrated a negative impact of agricultural development on European hares^79–81^. While forests are not typically preferred habitats for this species^82^, the “FORESTS” class in our study also includes successional phases with uncultivated meadow areas, which can support high densities of hares^83^. On the other hand, hares tend to avoid settlements (e.g.^84^). We observed a similar effect with forests in the European hare, likely as a response to large-scale croplands.

### Effects of wind farms

Our large-scale study revealed that wind farms had a slight negative impact on the abundance of roe deer and wild boar, though these effects were weak. Previous studies have suggested potential impacts of wind farms on roe deer^31,32^, but not specifically on wild boar^33^. Interestingly, in South Korea, where the wild boar population has also been affected by African Swine Fever^85^, study focused on forest habitats rather than agricultural landscapes^33^. This suggests that in forested areas where wild boars have adequate shelter, their response to wind farms may be less pronounced than in agricultural landscapes. In the study by Łopucki et al.^31^ in agricultural landscape, although there were insufficient wild boar tracks for detailed analysis, more tracks of this species were observed in control areas compared to within wind farms. The observed negative relationships of roe deer and wild boar with wind farms could also be influenced by hunters’ preferences rather than actual lower animal densities. Imperio et al.^86^ noted that hunting bags may better reflect animal density when considering hunting efforts. More hunters may result in more hunted individuals, higher hunting efficacy and more efficient control^87,88^. However, we could not account for the hunting effort in our study. To our knowledge, no research has assessed the impact of wind farms on hunting efforts or hunters’ preferences. Hunting effort is also difficult to assess. In the aging societies of Europe, many hunters hunt sporadically due to their age^89^. For this reason, in our assessment, not always the number of hunters but the number of hours spent in the hunting district could indicate hunting effort. Unfortunately, such data was not available to us. However, it should be noted that hunting bags were correlated with animal density, which may indicate that the results reflect wind farms’ impact on the density of roe deer and wild boar. This is confirmed by the results of other studies that showed avoidance of areas with wind farms by ungulates^30,31,34^.

The absence of an apparent effect of wind farms on mesocarnivores can largely be attributed to these species’ reliance on prey abundance (e.g.^70–72^). Previous research indicates that potential prey populations do not exhibit reduced abundance on wind farms. Some studies suggest that insect abundance may even be higher on wind farms than control areas, possibly due to the attractiveness of white turbine bases (e.g^90,91^). While vibrational noise from turbines can impact earthworms, the distance over which earthworm abundance is affected is typically short^92^. A similar situation applies to small mammals; despite observations of increased stress in some species on wind farms^93^, a clear negative impact on small mammal abundance has yet to be demonstrated^94,95^. Therefore, it is plausible that comparable prey abundance between wind farms and other areas results in similar mesocarnivore abundance. Furthermore, other benefits could offset any potential adverse effects of noise or other disturbances from operational turbines. For instance, mesocarnivores in wind farm areas might be attracted to carrion beneath turbines or find enhanced access to prey due to modified landscape features (e.g.^96,97^).

Our findings indicate that wind farms do not have a discernible impact on the European hare. Previous studies have shown varied responses of this species to wind farms, with reduced habitat utilization observed in Poland^31^, but no demonstrable effect seen in India^34^. Unlike other game mammals studied here, the density of hares is locally influenced by reintroduction efforts. The European hare is Poland’s most frequently reintroduced game mammal species, with several thousand individuals released annually^98,99^. Therefore, it becomes challenging to isolate the impact of wind farms in a population artificially bolstered by reintroductions. Nevertheless, given the high predation pressure attributed to the increased red fox population^100^, it remains plausible that wind farms could potentially influence the European hare. However, this would necessitate a more nuanced investigation, including an analysis of reintroduction efforts, which is beyond the scope of our current study.

## Conclusions

The study has highlighted that different cover types notably influence herbivorous species of game mammals in agricultural landscapes. These species generally thrive in farm areas with more natural landscape elements. For wild boars, there is a distinct avoidance of places with higher human activity, likely due to intensified hunting pressure. In contrast, mesocarnivores are primarily influenced by prey abundance with minimal observable effects of specific land cover types. The European polecat, however, showed a clear preference for forested areas.

Interestingly, only roe deer and wild boar negatively correlated with wind farms, while no significant effect was observed for European hares and mesocarnivores. The reasons underlying these relationships remain unclear and warrant further specific studies. The uncertainty regarding the causation of these effects currently impedes large-scale assessments of wind energy development on these studied game mammals. Wind farms represent a relatively new landscape feature, and the effects observed may be transient as animals potentially adapt to their presence. Conversely, greater coverage of wind farms across larger areas may lead to reduced densities of these herbivores, although predicting the threshold of such impacts remains challenging.

## Electronic supplementary material

Below is the link to the electronic supplementary material.

Supplementary Material 1

Supplementary Material 2

Supplementary Material 3

Supplementary Material 4

## Data Availability

Data is provided within supplementary information files.
