# Supplementary material for: The contingent impact of wind farms on game mammal density demonstrated in a large-scale analysis of hunting bag data in Poland
Source: Sci Rep. 2024 Oct 25;14:25290. doi: 10.1038/s41598-024-76999-2 (PMC11511958; doi:10.1038/s41598-024-76999-2)
Supplement: Supplementary file 1 — Supplementary Material 1 [file 41598_2024_76999_MOESM1_ESM.docx]

Table S1. Raw data on annual harvest of given species and percentage of main cover types in hunting grounds.

*Roe deer*

| Harvest  (ind./100ha) | SETTLEMENTS  (%) | CROPS  (%) | FORESTS  (%) | WATER  (%) | WIND FARM  (%) |
| --- | --- | --- | --- | --- | --- |
| 34.03 | 0.119335 | 0.541145 | 0.336507 | 0.003013 | 0.018434 |
| 29.8 | 0.032981 | 0.830037 | 0.110184 | 0.026798 | 0.145802 |
| 29.62 | 0.077906 | 0.776797 | 0.145296 | 0 | 0.026626 |
| 27.54 | 0.039841 | 0.816561 | 0.143598 | 0 | 0.101836 |
| 24.64 | 0.098285 | 0.880774 | 0.020941 | 0 | 0.173938 |
| 23.29 | 0.041239 | 0.678391 | 0.280371 | 0 | 0.166558 |
| 21.45 | 0.065776 | 0.916671 | 0.017553 | 0 | 0.021232 |
| 21.04 | 0.040076 | 0.833117 | 0.087333 | 0.039474 | 0.241711 |
| 20.28 | 0.095858 | 0.675914 | 0.223198 | 0.005031 | 0.017825 |
| 20.11 | 0.059243 | 0.805019 | 0.135738 | 0 | 0.094313 |
| 20.07 | 0.026162 | 0.710156 | 0.240745 | 0.022937 | 0.031545 |
| 19.51 | 0.058289 | 0.8764 | 0.065311 | 0 | 0.202343 |
| 18.85 | 0.134499 | 0.702936 | 0.162566 | 0 | 0.002641 |
| 18.72 | 0.054088 | 0.749397 | 0.163075 | 0.03344 | 0.116868 |
| 18.49 | 0.034773 | 0.709889 | 0.247028 | 0.008309 | 0.059819 |
| 18.35 | 0.03717 | 0.760903 | 0.201927 | 0 | 0.006919 |
| 18.29 | 0.029013 | 0.883584 | 0.082962 | 0.004441 | 0.000183 |
| 18.22 | 0.086936 | 0.888209 | 0.024855 | 0 | 0.080122 |
| 18.18 | 0.046194 | 0.608006 | 0.345294 | 0.000506 | 0.002681 |
| 17.92 | 0.046144 | 0.680903 | 0.254461 | 0.018493 | 0.000879 |
| 17.81 | 0.041876 | 0.634991 | 0.323132 | 0 | 0.019803 |
| 17.52 | 0.018482 | 0.571366 | 0.403494 | 0.006658 | 0.08103 |
| 17.3 | 0.028701 | 0.880606 | 0.090693 | 0 | 0.1192 |
| 17.16 | 0.056263 | 0.943737 | 0 | 0 | 0.41748 |
| 17.04 | 0.037227 | 0.774049 | 0.188724 | 0 | 0.136711 |
| 16.74 | 0.033275 | 0.900866 | 0.060343 | 0.005515 | 0.091831 |
| 16.73 | 0.048241 | 0.890315 | 0.061444 | 0 | 0.004877 |
| 16.63 | 0.067705 | 0.60951 | 0.322784 | 0 | 0.031049 |
| 16.47 | 0.122952 | 0.730519 | 0.14653 | 0 | 0.089381 |
| 16.44 | 0.09225 | 0.835858 | 0.071892 | 0 | 0.064066 |
| 16.27 | 0.032721 | 0.728697 | 0.238522 | 5.97E-05 | 0.018052 |
| 16.27 | 0.083189 | 0.898119 | 0.012762 | 0.00593 | 0.16835 |
| 16.15 | 0.029573 | 0.84438 | 0.121215 | 0.004832 | 0.031662 |
| 16.14 | 0.081238 | 0.718524 | 0.200238 | 0 | 0.013759 |
| 16.05 | 0.04601 | 0.785236 | 0.14245 | 0.026304 | 0.131842 |
| 15.79 | 0.034684 | 0.90422 | 0.061095 | 0 | 0.079926 |
| 15.74 | 0.028556 | 0.832744 | 0.1387 | 0 | 0.027699 |
| 15.54 | 0.063263 | 0.772515 | 0.155506 | 0.008716 | 0.17232 |
| 15.32 | 0.049629 | 0.944396 | 0.005975 | 0 | 0.171729 |
| 15.29 | 0.042009 | 0.716236 | 0.241755 | 0 | 0.25503 |
| 15.28 | 0.031001 | 0.661591 | 0.277932 | 0.029476 | 0.000647 |
| 15.19 | 0.059836 | 0.773228 | 0.059752 | 0.107184 | 0.135669 |
| 15.18 | 0.01221 | 0.707236 | 0.280555 | 0 | 0.010554 |
| 15.13 | 0.08025 | 0.857246 | 0.062497 | 7.34E-06 | 0.108955 |
| 15.07 | 0.003406 | 0.599878 | 0.396716 | 0 | 0.045399 |
| 14.98 | 0.216664 | 0.779225 | 0.004111 | 0 | 0.294511 |
| 14.9 | 0.035057 | 0.565328 | 0.393756 | 0.005859 | 0.098151 |
| 14.73 | 0.065419 | 0.70728 | 0.227301 | 0 | 0.288139 |
| 14.6 | 0.110983 | 0.870325 | 0.018692 | 0 | 0.141334 |
| 14.58 | 0.077148 | 0.72609 | 0.196763 | 0 | 0.022359 |
| 14.47 | 0.027274 | 0.638686 | 0.329299 | 0.004741 | 0.087119 |
| 14.47 | 0.102583 | 0.897417 | 0 | 0 | 0.045341 |
| 14.41 | 0.045934 | 0.924793 | 0.02209 | 0.007184 | 0.19778 |
| 14.36 | 0.049178 | 0.932341 | 0.018482 | 0 | 0.034648 |
| 14.31 | 0.092472 | 0.841024 | 0.066505 | 0 | 0.094696 |
| 14.14 | 0.008032 | 0.962155 | 0.004333 | 0.02548 | 0.026076 |
| 14.13 | 0.042169 | 0.597119 | 0.360712 | 0 | 0.036652 |
| 14.1 | 0.095985 | 0.700639 | 0.198975 | 0.0044 | 0.02055 |
| 13.98 | 0.064382 | 0.647883 | 0.274131 | 0.013604 | 0.054466 |
| 13.83 | 0.033553 | 0.805444 | 0.155166 | 0.005836 | 0.089808 |
| 13.82 | 0.102823 | 0.881072 | 0.016105 | 0 | 0.048898 |
| 13.79 | 0.134972 | 0.724281 | 0.120111 | 0.020636 | 0.008062 |
| 13.78 | 0.050611 | 0.799196 | 0.130295 | 0.019897 | 0.045145 |
| 13.75 | 0.029603 | 0.751571 | 0.218827 | 0 | 0.079736 |
| 13.74 | 0.070667 | 0.90593 | 0.02212 | 0.001283 | 0.083461 |
| 13.64 | 0.032421 | 0.811665 | 0.155915 | 0 | 0.079009 |
| 13.61 | 0.035474 | 0.794716 | 0.16981 | 0 | 0.222865 |
| 13.45 | 0.103313 | 0.732831 | 0.163856 | 0 | 0.045637 |
| 13.3 | 0.012081 | 0.564216 | 0.390193 | 0.03351 | 0.035279 |
| 13.25 | 0.130716 | 0.674756 | 0.194527 | 0 | 0.138547 |
| 13.21 | 0.025498 | 0.828399 | 0.114062 | 0.032041 | 0.078826 |
| 13.16 | 0.025165 | 0.709589 | 0.265246 | 0 | 0.340679 |
| 13.14 | 0.041847 | 0.933975 | 0 | 0.024178 | 0.027727 |
| 13.13 | 0.067136 | 0.715851 | 0.217013 | 0 | 0.03475 |
| 13.1 | 0.066768 | 0.576755 | 0.179528 | 0.176949 | 0.046495 |
| 13.09 | 0.032002 | 0.781137 | 0.152364 | 0.034498 | 0.000807 |
| 13.04 | 0.058187 | 0.528333 | 0.413479 | 0 | 0.002676 |
| 13.03 | 0.013851 | 0.854947 | 0.094772 | 0.036431 | 0.005141 |
| 12.94 | 0.019528 | 0.767714 | 0.212758 | 0 | 0.030766 |
| 12.91 | 0.029683 | 0.738291 | 0.186006 | 0.046019 | 0.017025 |
| 12.89 | 0.028434 | 0.931806 | 0.027306 | 0.012453 | 0.127255 |
| 12.88 | 0.029263 | 0.64246 | 0.263832 | 0.064444 | 0.068015 |
| 12.84 | 0.06371 | 0.687375 | 0.248915 | 0 | 0.024296 |
| 12.81 | 0.124811 | 0.814342 | 0.060847 | 0 | 0.105066 |
| 12.76 | 0.139615 | 0.679458 | 0.17389 | 0.007037 | 0.000136 |
| 12.75 | 0.041854 | 0.859523 | 0.094217 | 0.004407 | 0.152709 |
| 12.73 | 0.018303 | 0.841083 | 0.134267 | 0.006347 | 0.007471 |
| 12.71 | 0.072848 | 0.708754 | 0.212019 | 0.006379 | 0.2346 |
| 12.6 | 0.049416 | 0.637355 | 0.298827 | 0.014403 | 0.051553 |
| 12.54 | 0.108214 | 0.736279 | 0.126343 | 0.029164 | 0.000839 |
| 12.49 | 0.142246 | 0.407022 | 0.377298 | 0.073434 | 0.089493 |
| 12.44 | 0.005309 | 0.943367 | 0.02594 | 0.025384 | 0.000813 |
| 12.27 | 0.046989 | 0.861338 | 0.091672 | 0 | 0.288311 |
| 12.19 | 0.030907 | 0.705282 | 0.26381 | 0 | 0.210419 |
| 12.18 | 0.053764 | 0.773062 | 0.173173 | 0 | 0.151376 |
| 12.12 | 0.029447 | 0.903798 | 0.066755 | 0 | 0.23376 |
| 12.12 | 0.050745 | 0.719471 | 0.229783 | 0 | 0.077093 |
| 12.12 | 0.01082 | 0.979523 | 0 | 0.009656 | 0.210532 |
| 12.03 | 0.011426 | 0.976589 | 0.011985 | 0 | 0.007056 |
| 12.02 | 0.04557 | 0.58709 | 0.36734 | 0 | 0.070527 |
| 12 | 0.051785 | 0.901516 | 0.046699 | 0 | 0.007171 |
| 12 | 0.023232 | 0.748449 | 0.204923 | 0.023397 | 0.029774 |
| 11.99 | 0.007157 | 0.760099 | 0.232744 | 0 | 0.138899 |
| 11.98 | 0.025046 | 0.688707 | 0.286247 | 0 | 0.026522 |
| 11.87 | 0.017755 | 0.694353 | 0.28756 | 0.000331 | 0.007311 |
| 11.86 | 0.03505 | 0.940453 | 0 | 0.024497 | 0.000582 |
| 11.86 | 0.020053 | 0.595764 | 0.384183 | 0 | 0.022035 |
| 11.85 | 0.137764 | 0.692755 | 0.169481 | 0 | 0.01181 |
| 11.8 | 0.015311 | 0.850012 | 0.134677 | 0 | 0.043252 |
| 11.78 | 0.003095 | 0.950925 | 0.015182 | 0.030799 | 0.103433 |
| 11.75 | 0.043989 | 0.711189 | 0.244822 | 0 | 0.171178 |
| 11.72 | 0.060899 | 0.775398 | 0.163702 | 0 | 0.022238 |
| 11.71 | 0.02159 | 0.743128 | 0.235281 | 0 | 0.058284 |
| 11.69 | 0.037281 | 0.903436 | 0.042729 | 0.016554 | 0.047312 |
| 11.68 | 0.021317 | 0.804667 | 0.174016 | 0 | 0.278978 |
| 11.57 | 0.12639 | 0.853682 | 0.019928 | 0 | 0.018693 |
| 11.57 | 0.050548 | 0.895549 | 0.053477 | 0.000426 | 0.040513 |
| 11.51 | 0.070103 | 0.80001 | 0.098141 | 0.031745 | 0.019618 |
| 11.5 | 0.011447 | 0.645638 | 0.323308 | 0.019607 | 0.001809 |
| 11.47 | 0.014883 | 0.952852 | 0.026053 | 0.006211 | 0.039717 |
| 11.41 | 0.027512 | 0.92664 | 0.024607 | 0.02124 | 0.166807 |
| 11.41 | 0.078413 | 0.768356 | 0.153232 | 0 | 0.086105 |
| 11.34 | 0.028999 | 0.817861 | 0.15314 | 0 | 0.078066 |
| 11.32 | 0.100035 | 0.878576 | 0 | 0.021389 | 0.004507 |
| 11.32 | 0.01712 | 0.800569 | 0.132823 | 0.049489 | 0.06699 |
| 11.15 | 0.012828 | 0.801466 | 0.169907 | 0.015799 | 0.035618 |
| 11.13 | 0.017442 | 0.520835 | 0.389462 | 0.072261 | 0.001443 |
| 11.11 | 0.098187 | 0.786894 | 0.114919 | 0 | 0.033695 |
| 11.07 | 0.043952 | 0.822823 | 0.084006 | 0.049219 | 0.044707 |
| 11 | 0.035435 | 0.823689 | 0.105185 | 0.035691 | 0.047584 |
| 10.97 | 0.017027 | 0.946215 | 0.036758 | 0 | 0.005504 |
| 10.96 | 0.007743 | 0.882418 | 0.104562 | 0.005277 | 0.030295 |
| 10.92 | 0.02038 | 0.918323 | 0.036001 | 0.025295 | 0.032543 |
| 10.91 | 0.049931 | 0.628573 | 0.321228 | 0.000269 | 0.036331 |
| 10.89 | 0.03637 | 0.8723 | 0.091329 | 0 | 0.000179 |
| 10.86 | 0.105751 | 0.72069 | 0.166077 | 0.007481 | 0.034231 |
| 10.86 | 0.040823 | 0.959177 | 0 | 0 | 0.004214 |
| 10.85 | 0.036829 | 0.673528 | 0.283352 | 0.006291 | 0.031069 |
| 10.79 | 0.078715 | 0.778306 | 0.133458 | 0.009521 | 0.022315 |
| 10.72 | 0.042068 | 0.913719 | 0.035636 | 0.008577 | 0.070134 |
| 10.7 | 0.016972 | 0.707628 | 0.225538 | 0.049862 | 0.111867 |
| 10.67 | 0.026381 | 0.950107 | 0 | 0.023512 | 0.343647 |
| 10.66 | 0.142331 | 0.782279 | 0.058797 | 0.016592 | 0.041235 |
| 10.66 | 0.022977 | 0.95925 | 0.006649 | 0.011124 | 0.025333 |
| 10.63 | 0.096224 | 0.839476 | 0.0643 | 0 | 0.055361 |
| 10.59 | 0.059068 | 0.905269 | 0.035663 | 0 | 0.061176 |
| 10.58 | 0.030864 | 0.930063 | 0 | 0.039073 | 0.003036 |
| 10.55 | 0.039739 | 0.691329 | 0.268932 | 0 | 0.195716 |
| 10.48 | 0.033849 | 0.683169 | 0.28066 | 0.002321 | 0.049347 |
| 10.48 | 0.073033 | 0.813903 | 0.097747 | 0.015317 | 0.19531 |
| 10.47 | 0.014978 | 0.876519 | 0.099945 | 0.008559 | 0.01608 |
| 10.44 | 0.035743 | 0.671162 | 0.284171 | 0.008924 | 0.015164 |
| 10.41 | 0.077519 | 0.685448 | 0.237032 | 0 | 0.018202 |
| 10.4 | 0.039287 | 0.659343 | 0.239964 | 0.061407 | 0.021402 |
| 10.39 | 0.143477 | 0.843699 | 0.006261 | 0.006563 | 0.063003 |
| 10.38 | 0.112847 | 0.540884 | 0.2825 | 0.063769 | 0.234756 |
| 10.34 | 0.03309 | 0.827144 | 0.139765 | 0 | 0.030663 |
| 10.31 | 0.072977 | 0.856731 | 0.070292 | 0 | 0.074098 |
| 10.16 | 0.040323 | 0.76684 | 0.192836 | 0 | 0.016819 |
| 10.15 | 0.069296 | 0.774471 | 0.070706 | 0.085528 | 0.031938 |
| 10.13 | 0.080504 | 0.831155 | 0.005685 | 0.082657 | 0.041065 |
| 10.12 | 0.018063 | 0.899386 | 0.044394 | 0.038157 | 0.085235 |
| 10.09 | 0.030872 | 0.686417 | 0.272097 | 0.010614 | 0.010509 |
| 10.07 | 0.065773 | 0.829725 | 0.103836 | 0.000666 | 0.181817 |
| 10.05 | 0.072147 | 0.370505 | 0.269155 | 0.273316 | 0.041596 |
| 10.03 | 0.001377 | 0.682013 | 0.307943 | 0.008666 | 0.024811 |
| 10 | 0.01264 | 0.937165 | 0.043062 | 0.007133 | 0.185625 |
| 9.92 | 0.033665 | 0.827557 | 0.095811 | 0.042966 | 0.085835 |
| 9.91 | 0.026258 | 0.530627 | 0.436197 | 0.006918 | 0.028396 |
| 9.9 | 0.046884 | 0.923363 | 0.029753 | 0 | 0.00521 |
| 9.89 | 0.055185 | 0.873829 | 0.070986 | 0 | 0.060068 |
| 9.86 | 0.058012 | 0.561444 | 0.380544 | 0 | 0.028502 |
| 9.81 | 0.02225 | 0.900537 | 0.067932 | 0.00928 | 0.000577 |
| 9.76 | 0.016859 | 0.887013 | 0.096128 | 0 | 0.07925 |
| 9.73 | 0.021006 | 0.91466 | 0.064334 | 0 | 0.031401 |
| 9.67 | 0.048814 | 0.912767 | 0.038419 | 0 | 0.001488 |
| 9.67 | 0.020504 | 0.893617 | 0.085879 | 0 | 0.028256 |
| 9.65 | 0.181455 | 0.617007 | 0.187585 | 0.013953 | 0.12495 |
| 9.57 | 0.025413 | 0.787728 | 0.186858 | 0 | 0.021162 |
| 9.56 | 0.02837 | 0.674304 | 0.282227 | 0.015099 | 0.162311 |
| 9.52 | 0.077374 | 0.85315 | 0.00706 | 0.062416 | 0.027737 |
| 9.49 | 0.02551 | 0.914172 | 0.045998 | 0.01432 | 0.02754 |
| 9.45 | 0.023883 | 0.761086 | 0.215031 | 0 | 0.033082 |
| 9.4 | 0.063612 | 0.891342 | 0.045046 | 0 | 0.107764 |
| 9.4 | 0.020569 | 0.771368 | 0.155748 | 0.052315 | 0.015713 |
| 9.39 | 0.07946 | 0.663096 | 0.257444 | 0 | 0.119837 |
| 9.36 | 0.074589 | 0.640797 | 0.26404 | 0.020574 | 0.021459 |
| 9.35 | 0.033544 | 0.839022 | 0.127434 | 0 | 0.062529 |
| 9.34 | 0.008526 | 0.940563 | 0.050911 | 0 | 0.124726 |
| 9.33 | 0.011822 | 0.922221 | 0.0577 | 0.008256 | 0.027789 |
| 9.3 | 0.037255 | 0.909058 | 0.053687 | 0 | 0.110157 |
| 9.26 | 0.036312 | 0.904341 | 0.024842 | 0.034504 | 0.160208 |
| 9.25 | 0.031111 | 0.552667 | 0.394879 | 0.021342 | 0.026238 |
| 9.2 | 0.04742 | 0.747043 | 0.205537 | 0 | 0.027979 |
| 9.19 | 0.009555 | 0.979002 | 0.000198 | 0.011245 | 0.076538 |
| 9.18 | 0.168176 | 0.81787 | 0.013954 | 0 | 0.049296 |
| 9.17 | 0.04838 | 0.768229 | 0.137646 | 0.045745 | 0.000874 |
| 9.16 | 0.005345 | 0.589222 | 0.392616 | 0.012817 | 0.022783 |
| 9.16 | 0.022281 | 0.817366 | 0.144456 | 0.015897 | 0.002968 |
| 9.15 | 0.059954 | 0.694479 | 0.245567 | 0 | 0.029688 |
| 9.15 | 0.08957 | 0.6941 | 0.21633 | 0 | 0.055495 |
| 9.13 | 0.028579 | 0.779842 | 0.184295 | 0.007283 | 0.00428 |
| 9.13 | 0.026939 | 0.973061 | 0 | 0 | 0.021787 |
| 9.1 | 0.071109 | 0.886488 | 0.042403 | 0 | 0.027537 |
| 9.07 | 0.070053 | 0.928604 | 0.001343 | 0 | 0.237388 |
| 9.06 | 0.029025 | 0.753063 | 0.182242 | 0.03567 | 0.000693 |
| 9.05 | 0.054568 | 0.700053 | 0.236636 | 0.008742 | 0.051978 |
| 9.04 | 0.062328 | 0.768301 | 0.146098 | 0.023273 | 0.041255 |
| 9.02 | 0.032579 | 0.759046 | 0.200145 | 0.008229 | 0.014697 |
| 8.98 | 0.004923 | 0.757164 | 0.144813 | 0.0931 | 0.011441 |
| 8.96 | 0.060047 | 0.690705 | 0.249248 | 0 | 0.327264 |
| 8.92 | 0.039 | 0.770268 | 0.167469 | 0.023263 | 0.142616 |
| 8.91 | 0.010139 | 0.929534 | 0.039062 | 0.021265 | 0.02452 |
| 8.89 | 0.029925 | 0.788213 | 0.181862 | 0 | 0.020835 |
| 8.85 | 0.024747 | 0.964457 | 0.010796 | 0 | 0.256071 |
| 8.85 | 0.092208 | 0.70089 | 0.190913 | 0.015988 | 0.053543 |
| 8.84 | 0.055552 | 0.69975 | 0.239609 | 0.005088 | 0.003034 |
| 8.8 | 0.03957 | 0.723527 | 0.232789 | 0.004115 | 0.000244 |
| 8.77 | 0.024205 | 0.53313 | 0.202177 | 0.240487 | 0.002058 |
| 8.76 | 0.045044 | 0.886899 | 0.026934 | 0.041122 | 0.00106 |
| 8.72 | 0.182413 | 0.806462 | 0.011125 | 0 | 0.037714 |
| 8.72 | 0.048955 | 0.647511 | 0.303534 | 0 | 0.045488 |
| 8.63 | 0.086482 | 0.913518 | 0 | 0 | 0.005667 |
| 8.63 | 0.074807 | 0.798245 | 0.126948 | 0 | 0.052297 |
| 8.61 | 0.020858 | 0.994368 | 0.0019 | 0 | 0.003386 |
| 8.6 | 0.101092 | 0.659995 | 0.223647 | 0.015266 | 0.004566 |
| 8.58 | 0.014212 | 0.940359 | 0.02911 | 0.01632 | 0.085242 |
| 8.57 | 0.069932 | 0.770161 | 0.159907 | 0 | 0.300129 |
| 8.55 | 0.075708 | 0.831813 | 0.092479 | 0 | 0.125231 |
| 8.53 | 0.01543 | 0.653371 | 0.299888 | 0.031312 | 0.052174 |
| 8.53 | 0.050315 | 0.598024 | 0.351513 | 0.000147 | 0.038121 |
| 8.53 | 0.039445 | 0.880548 | 0.078509 | 0.001499 | 0.00146 |
| 8.52 | 0.070047 | 0.841396 | 0.083412 | 0.005146 | 0.038956 |
| 8.52 | 0.022756 | 0.827933 | 0.149312 | 0 | 0.036017 |
| 8.52 | 0.023363 | 0.846999 | 0.129638 | 0 | 0.078641 |
| 8.5 | 0.036346 | 0.809759 | 0.128229 | 0.025666 | 0.037496 |
| 8.49 | 0.050363 | 0.949637 | 0 | 0 | 0.000103 |
| 8.45 | 0.074518 | 0.828824 | 0.096658 | 0 | 0.049843 |
| 8.45 | 0.042539 | 0.616348 | 0.341113 | 0 | 0.050441 |
| 8.44 | 0.012225 | 0.952622 | 0.035153 | 0 | 0.01266 |
| 8.41 | 0.077189 | 0.793972 | 0.122556 | 0.006282 | 0.067732 |
| 8.4 | 0.097723 | 0.899411 | 0.002866 | 0 | 0.018141 |
| 8.4 | 0.041745 | 0.712863 | 0.243395 | 0.001997 | 0.02306 |
| 8.37 | 0.038 | 0.931754 | 0 | 0.030246 | 7.47E-05 |
| 8.36 | 0.022698 | 0.676984 | 0.300318 | 0 | 0.086161 |
| 8.34 | 0.046235 | 0.768727 | 0.185038 | 0 | 0.003722 |
| 8.34 | 0.08742 | 0.91258 | 0 | 0 | 0.040064 |
| 8.34 | 0.072839 | 0.741172 | 0.185989 | 0 | 0.02913 |
| 8.29 | 0.028501 | 0.93417 | 0.037329 | 0 | 7.53E-06 |
| 8.29 | 0.039022 | 0.789893 | 0.171085 | 0 | 0.011916 |
| 8.27 | 0.096072 | 0.863331 | 0.040596 | 0 | 0.08757 |
| 8.26 | 0.060716 | 0.682299 | 0.256985 | 0 | 0.053094 |
| 8.25 | 0.04306 | 0.876667 | 0.080273 | 0 | 0.034083 |
| 8.19 | 0.012158 | 0.877799 | 0.104953 | 0.00509 | 0.119091 |
| 8.14 | 0.130492 | 0.869508 | 0 | 0 | 0.036249 |
| 8.14 | 0.023913 | 0.904383 | 0.050222 | 0.021483 | 0.365279 |
| 8.11 | 0.037805 | 0.73086 | 0.231335 | 0 | 0.156062 |
| 8.1 | 0.099347 | 0.900653 | 0 | 0 | 0.147427 |
| 8.09 | 0.18621 | 0.781401 | 0.032389 | 0 | 0.147147 |
| 8.01 | 0.502659 | 0.453471 | 0.003471 | 0.040398 | 0.154066 |
| 8 | 0.029173 | 0.847523 | 0.123304 | 0 | 0.02368 |
| 8 | 0.045414 | 0.745517 | 0.195632 | 0.013437 | 0.031831 |
| 7.97 | 0.010053 | 0.727504 | 0.207121 | 0.055322 | 0.048494 |
| 7.94 | 0 | 0.891838 | 0.108162 | 0 | 0.002346 |
| 7.93 | 0.039215 | 0.907213 | 0.053573 | 0 | 0.070329 |
| 7.93 | 0.073048 | 0.7756 | 0.151352 | 0 | 0.061654 |
| 7.82 | 0.010679 | 0.952 | 0.001661 | 0.035661 | 0.004639 |
| 7.77 | 0.085555 | 0.904837 | 0.009608 | 0 | 0.034194 |
| 7.76 | 0.032947 | 0.773658 | 0.145192 | 0.048202 | 0.049896 |
| 7.75 | 0.21499 | 0.702711 | 0.080053 | 0.002246 | 0.007246 |
| 7.7 | 0.020372 | 0.836787 | 0.071528 | 0.071313 | 0.01532 |
| 7.69 | 0.037419 | 0.923312 | 0.010583 | 0.028685 | 0.061092 |
| 7.67 | 0.11188 | 0.782154 | 0.105966 | 0 | 0.001959 |
| 7.66 | 0.055696 | 0.879582 | 0.060887 | 0.003835 | 0.021538 |
| 7.66 | 0.01216 | 0.758907 | 0.228932 | 0 | 0.023113 |
| 7.65 | 0.030802 | 0.910639 | 0.052474 | 0.006085 | 0.34264 |
| 7.63 | 0.027304 | 0.917848 | 0.04837 | 0.006477 | 0.349861 |
| 7.63 | 0.041595 | 0.871269 | 0.087137 | 0 | 0.085193 |
| 7.62 | 0.030385 | 0.902018 | 0.026566 | 0.041031 | 0.017606 |
| 7.61 | 0.029175 | 0.877794 | 0.093031 | 0 | 0.015471 |
| 7.55 | 0.069289 | 0.841813 | 0.088899 | 0 | 0.074837 |
| 7.54 | 0.05676 | 0.713201 | 0.23004 | 0 | 0.025905 |
| 7.52 | 0.015126 | 0.759174 | 0.204304 | 0.021397 | 0.097869 |
| 7.51 | 0.02289 | 0.955775 | 0.021335 | 0 | 0.120855 |
| 7.48 | 0.0432 | 0.9568 | 0 | 0 | 0.013619 |
| 7.47 | 0.026149 | 0.925556 | 0.048294 | 0 | 0.048499 |
| 7.46 | 0.040363 | 0.948775 | 0.010863 | 0 | 0.06336 |
| 7.46 | 0.069142 | 0.772286 | 0.14675 | 0.011822 | 0.039618 |
| 7.45 | 0.071363 | 0.916463 | 0.012174 | 0 | 0.181614 |
| 7.44 | 0.012941 | 0.611311 | 0.36866 | 0.007087 | 0.050402 |
| 7.43 | 0.141596 | 0.638012 | 0.156134 | 0.064258 | 0.108228 |
| 7.4 | 0.038604 | 0.954512 | 0.006884 | 0 | 0.02608 |
| 7.38 | 0.051831 | 0.828658 | 0.11951 | 0 | 0.107904 |
| 7.36 | 0.041801 | 0.944368 | 0 | 0.013831 | 0.010372 |
| 7.34 | 0.180694 | 0.813429 | 0 | 0.005877 | 0.020059 |
| 7.3 | 0.012366 | 0.881566 | 0.053689 | 0.052379 | 0.104641 |
| 7.29 | 0.020885 | 0.97362 | 0.005495 | 0 | 0.031392 |
| 7.25 | 0.007404 | 0.887462 | 0.105134 | 0 | 0.179264 |
| 7.24 | 0.021546 | 0.785222 | 0.093462 | 0.09977 | 0.001934 |
| 7.24 | 0.041867 | 0.696324 | 0.261809 | 0 | 0.009857 |
| 7.2 | 0.177178 | 0.698147 | 0.059042 | 0.065633 | 0.169988 |
| 7.19 | 0.032119 | 0.666449 | 0.28536 | 0.016073 | 0.089523 |
| 7.19 | 0.045204 | 0.596497 | 0.333565 | 0.01242 | 0.118649 |
| 7.17 | 0.139056 | 0.729618 | 0.11691 | 0.014416 | 2.54E-05 |
| 7.16 | 0.017507 | 0.974532 | 0.00609 | 0.001872 | 0.18464 |
| 7.14 | 0.056828 | 0.90978 | 0.014798 | 0.018594 | 0.01458 |
| 7.08 | 0.151948 | 0.530499 | 0.120228 | 0.197325 | 0.019356 |
| 7.03 | 0.022113 | 0.740767 | 0.237121 | 0 | 0.271627 |
| 7.03 | 0.034103 | 0.818442 | 0.147454 | 0 | 0.087954 |
| 7.02 | 0.064454 | 0.807537 | 0.118761 | 0.009248 | 0.039809 |
| 7.02 | 0.020796 | 0.921761 | 0.057443 | 0 | 0.011385 |
| 7.01 | 0.049055 | 0.900592 | 0.050353 | 0 | 0.099 |
| 6.98 | 0.007534 | 0.991543 | 0 | 0.000922 | 0.020689 |
| 6.96 | 0.030193 | 0.919865 | 0.019158 | 0.030784 | 0.093644 |
| 6.95 | 0.017478 | 0.896971 | 0.020908 | 0.064643 | 0.124726 |
| 6.92 | 0.019597 | 0.789053 | 0.19135 | 0 | 0.007049 |
| 6.91 | 0.092467 | 0.907533 | 0 | 0 | 0.110143 |
| 6.91 | 0.035536 | 0.632685 | 0.323556 | 0.008224 | 0.021802 |
| 6.85 | 0.279126 | 0.620004 | 0.100869 | 0 | 0.032547 |
| 6.85 | 0.137039 | 0.844241 | 0.01872 | 0 | 0.054031 |
| 6.79 | 0.052708 | 0.717515 | 0.2169 | 0.012876 | 0.308219 |
| 6.79 | 0.006056 | 0.778152 | 0.174763 | 0.041029 | 0.032651 |
| 6.74 | 0.034493 | 0.812457 | 0.153051 | 0 | 0.073334 |
| 6.7 | 0.028499 | 0.562559 | 0.360137 | 0.048805 | 0.001166 |
| 6.69 | 0.05913 | 0.575212 | 0.365601 | 5.72E-05 | 0.03847 |
| 6.68 | 0.009447 | 0.761519 | 0.21221 | 0.016824 | 0.01016 |
| 6.61 | 0.058021 | 0.662699 | 0.258411 | 0.020869 | 0.038288 |
| 6.58 | 0.021748 | 0.928259 | 0.049992 | 7.31E-07 | 0.043612 |
| 6.57 | 0.033119 | 0.862447 | 0.104434 | 0 | 0.024761 |
| 6.52 | 0.018223 | 0.50482 | 0.425159 | 0 | 0.016896 |
| 6.51 | 0.012878 | 0.947298 | 0.03675 | 0.003074 | 0.028568 |
| 6.5 | 0.111642 | 0.775675 | 0.112682 | 0 | 0.047805 |
| 6.41 | 0.077048 | 0.723402 | 0.19955 | 0 | 0.077261 |
| 6.38 | 0.046998 | 0.826689 | 0.126312 | 0 | 0.087959 |
| 6.35 | 0.012289 | 0.967888 | 0.019822 | 0 | 0.169411 |
| 6.32 | 0.028708 | 0.609643 | 0.361648 | 0 | 0.082055 |
| 6.31 | 0.046888 | 0.885726 | 0.036393 | 0.030993 | 0.012365 |
| 6.28 | 0.017962 | 0.804942 | 0.163669 | 0.013427 | 0.006701 |
| 6.25 | 0.039363 | 0.856503 | 0.095384 | 0.00875 | 0.023128 |
| 6.24 | 0.028831 | 0.777557 | 0.193612 | 0 | 0.01115 |
| 6.22 | 0.041708 | 0.943745 | 0 | 0.014547 | 0.008971 |
| 6.13 | 0.008343 | 0.991657 | 0 | 0 | 0.039714 |
| 6.13 | 0.036323 | 0.95832 | 0.005356 | 0 | 0.348035 |
| 6.12 | 0.052284 | 0.743996 | 0.193735 | 0.009985 | 0.045387 |
| 6.05 | 0.034088 | 0.734203 | 0.231709 | 0 | 0.003313 |
| 6.04 | 0.036485 | 0.81515 | 0.148365 | 0 | 0.19155 |
| 6.03 | 0.021547 | 0.897054 | 0.081399 | 0 | 0.003304 |
| 6.03 | 0.035595 | 0.757742 | 0.206663 | 0 | 0.026593 |
| 6.01 | 0.019964 | 0.944058 | 0.034331 | 0.001647 | 0.054066 |
| 6.01 | 0.013546 | 0.804015 | 0.125482 | 0.056958 | 0.043361 |
| 5.97 | 0.042971 | 0.949884 | 0.007145 | 0 | 0.291543 |
| 5.93 | 0.04474 | 0.643511 | 0.311749 | 0 | 0.033318 |
| 5.89 | 0.04377 | 0.927657 | 0.028573 | 0 | 0.115429 |
| 5.83 | 0.021323 | 0.624195 | 0.354482 | 0 | 0.002746 |
| 5.82 | 0.153329 | 0.620561 | 0.18755 | 0.031629 | 0.091145 |
| 5.8 | 0.032367 | 0.938775 | 0.019579 | 0.009278 | 0.008665 |
| 5.8 | 0.041354 | 0.793022 | 0.109663 | 0.055961 | 0.156689 |
| 5.8 | 0.01756 | 0.936756 | 0.045684 | 0 | 0.587545 |
| 5.73 | 0.058124 | 0.890746 | 0.007969 | 0.043162 | 0.066551 |
| 5.72 | 0.01512 | 0.858559 | 0.07309 | 0.053231 | 0.037677 |
| 5.7 | 0.061675 | 0.89241 | 0.045559 | 0.000356 | 0.165958 |
| 5.7 | 0.059338 | 0.879605 | 0.056493 | 0 | 0.222732 |
| 5.66 | 0.044084 | 0.893109 | 0.062806 | 0 | 0.010772 |
| 5.66 | 0.025682 | 0.856233 | 0.069107 | 0.048978 | 0.562058 |
| 5.64 | 0.059391 | 0.887374 | 0.052975 | 0.00026 | 0.002722 |
| 5.62 | 0.028722 | 0.608216 | 0.319598 | 0.043464 | 0.002594 |
| 5.61 | 0.021306 | 0.96547 | 0.000434 | 0.01279 | 0.093825 |
| 5.55 | 0.076638 | 0.893076 | 0.030286 | 0 | 0.003956 |
| 5.52 | 0.02416 | 0.973681 | 0.00216 | 0 | 0.016484 |
| 5.5 | 0.025726 | 0.568486 | 0.358865 | 0.046922 | 0.138587 |
| 5.41 | 0.222442 | 0.74335 | 0.034208 | 0 | 0.006338 |
| 5.36 | 0.016397 | 0.951369 | 0.032233 | 0 | 0.022951 |
| 5.34 | 0.032845 | 0.784505 | 0.155122 | 0.027527 | 0.09242 |
| 5.34 | 0.061571 | 0.728748 | 0.209681 | 0 | 0.041562 |
| 5.24 | 0.066093 | 0.856123 | 0.077784 | 0 | 0.032206 |
| 5.22 | 0.036113 | 0.84386 | 0.028627 | 0.0914 | 0.01802 |
| 5.21 | 0.035856 | 0.89092 | 0.073224 | 0 | 0.152419 |
| 5.21 | 0.053152 | 0.914463 | 0.025729 | 0.006655 | 0.029585 |
| 5.19 | 0.054189 | 0.877665 | 0.068146 | 0 | 0.141787 |
| 5.17 | 0.079122 | 0.728452 | 0.189573 | 0.002853 | 0.013886 |
| 5.16 | 0.086829 | 0.552521 | 0.36065 | 0 | 0.123484 |
| 5.13 | 0.167614 | 0.459503 | 0.216179 | 0.156704 | 0.034594 |
| 5.13 | 0.04376 | 0.936753 | 0.019487 | 0 | 0.061764 |
| 5.1 | 0.114896 | 0.770582 | 0.111487 | 0.003035 | 0.057439 |
| 5.08 | 0.059469 | 0.940531 | 0 | 0 | 0.036402 |
| 5.07 | 0.015465 | 0.974209 | 0.010326 | 0 | 0.001597 |
| 5.07 | 0.009221 | 0.887556 | 0.073236 | 0.029987 | 0.245076 |
| 5.07 | 0.06005 | 0.93995 | 0 | 0 | 0.023202 |
| 5.06 | 0.043407 | 0.941455 | 0.015138 | 0 | 0.002294 |
| 5.02 | 0.072476 | 0.909047 | 0.018477 | 0 | 0.060021 |
| 4.99 | 0.014656 | 0.937846 | 0.041141 | 0.006357 | 0.017017 |
| 4.96 | 0.053113 | 0.649051 | 0.261498 | 0.036338 | 0.042373 |
| 4.91 | 0.045738 | 0.828349 | 0.125913 | 0 | 0.022493 |
| 4.85 | 0.051852 | 0.630668 | 0.304929 | 0.01255 | 0.049537 |
| 4.84 | 0.052255 | 0.898211 | 0.046067 | 0.003468 | 0.000502 |
| 4.83 | 0.043956 | 0.854424 | 0.090371 | 0.011248 | 0.042874 |
| 4.82 | 0.025069 | 0.954991 | 0.01994 | 0 | 0.029211 |
| 4.81 | 0.032369 | 0.54685 | 0.415187 | 0.005593 | 0.023739 |
| 4.8 | 0.096755 | 0.818844 | 0.082158 | 0.002244 | 0.031972 |
| 4.79 | 0.048003 | 0.729414 | 0.209387 | 0.013197 | 0.033985 |
| 4.79 | 0.064139 | 0.813701 | 0.122161 | 0 | 0.111806 |
| 4.74 | 0.101064 | 0.634403 | 0.264178 | 0.000356 | 0.002776 |
| 4.74 | 0.019281 | 0.635579 | 0.264739 | 0.080401 | 0.007959 |
| 4.72 | 0.065527 | 0.728519 | 0.126969 | 0.078986 | 0.047136 |
| 4.69 | 0.004494 | 0.931113 | 0.025152 | 0.03924 | 0.009948 |
| 4.68 | 0.016053 | 0.877654 | 0.080995 | 0.025299 | 0.022378 |
| 4.64 | 0.03398 | 0.965065 | 0.000956 | 0 | 0.457655 |
| 4.64 | 0.031613 | 0.961818 | 0.003158 | 0.003411 | 0.046069 |
| 4.63 | 0.04551 | 0.731711 | 0.222779 | 0 | 0.007162 |
| 4.63 | 0.018798 | 0.873342 | 0.096714 | 0.011147 | 0.000615 |
| 4.49 | 0.049996 | 0.803418 | 0.146586 | 0 | 0.137218 |
| 4.45 | 0.072737 | 0.703643 | 0.172948 | 0.050672 | 0.003121 |
| 4.44 | 0.023466 | 0.646274 | 0.253975 | 0.066486 | 0.024883 |
| 4.42 | 0.043339 | 0.663185 | 0.278789 | 0.014687 | 0.102922 |
| 4.41 | 0.110639 | 0.740989 | 0.108626 | 0.039746 | 0.079146 |
| 4.41 | 0.077451 | 0.901627 | 0.020923 | 0 | 0.037246 |
| 4.38 | 0.063452 | 0.859274 | 0.077275 | 0 | 0.167713 |
| 4.35 | 0.045885 | 0.95365 | 0.000465 | 0 | 0.003323 |
| 4.34 | 0.224025 | 0.744055 | 0.03192 | 0 | 0.080825 |
| 4.24 | 0.091309 | 0.876741 | 0.03195 | 0 | 0.02242 |
| 4.2 | 0.041492 | 0.83554 | 0.122968 | 0 | 0.236861 |
| 4.12 | 0.034667 | 0.836308 | 0.12772 | 0.001305 | 0.026518 |
| 4.11 | 0.080689 | 0.873851 | 0.041035 | 0.004425 | 0.180591 |
| 3.96 | 0.031482 | 0.918601 | 0 | 0.049917 | 0.016405 |
| 3.96 | 0.059471 | 0.75569 | 0.181554 | 0.003285 | 0.032832 |
| 3.95 | 0.068853 | 0.901758 | 0.029389 | 0 | 0.153666 |
| 3.94 | 0.032047 | 0.620483 | 0.34747 | 0 | 0.032438 |
| 3.91 | 0.009634 | 0.907754 | 0.082612 | 0 | 0.016301 |
| 3.9 | 0.027717 | 0.596513 | 0.370443 | 0.005327 | 0.035165 |
| 3.88 | 0.032386 | 0.701748 | 0.255853 | 0.010013 | 0.027642 |
| 3.87 | 0.072101 | 0.86385 | 0.054567 | 0.009482 | 0.064817 |
| 3.8 | 0.12475 | 0.506296 | 0.368954 | 0 | 0.018843 |
| 3.76 | 0.053881 | 0.915746 | 0.006846 | 0.023526 | 0.034166 |
| 3.75 | 0.016337 | 0.866699 | 0.111594 | 0.00537 | 0.013295 |
| 3.71 | 0.025748 | 0.720481 | 0.253771 | 0 | 0.036214 |
| 3.7 | 0.01259 | 0.695308 | 0.274294 | 0.017807 | 0.002173 |
| 3.7 | 0.105629 | 0.788264 | 0.072893 | 0.033215 | 0.044414 |
| 3.67 | 0.019231 | 0.918595 | 0.062174 | 0 | 0.02441 |
| 3.63 | 0.030852 | 0.937414 | 0.022909 | 0.008825 | 0.063511 |
| 3.54 | 0.067538 | 0.901478 | 0.024953 | 0.00603 | 0.018914 |
| 3.46 | 0.030356 | 0.662937 | 0.306707 | 0 | 0.021427 |
| 3.45 | 0.157547 | 0.729757 | 0.10907 | 0.003625 | 0.027969 |
| 3.44 | 0.030564 | 0.913953 | 0.055484 | 0 | 0.087083 |
| 3.37 | 0.03372 | 0.954883 | 0.011397 | 0 | 0.059214 |
| 3.32 | 0.135385 | 0.837915 | 0.0267 | 0 | 0.059579 |
| 3.27 | 0.048432 | 0.813801 | 0.137767 | 0 | 0.016043 |
| 3.25 | 0.027087 | 0.892398 | 0.06958 | 0.010935 | 0.015594 |
| 3.21 | 0.07399 | 0.807539 | 0.022472 | 0.096 | 0.028334 |
| 3.19 | 0.026963 | 0.570467 | 0.183307 | 0.219263 | 0.0174 |
| 3.16 | 0.203607 | 0.526209 | 0.270184 | 0 | 0.050672 |
| 3.16 | 0.039863 | 0.711922 | 0.248216 | 0 | 0.020078 |
| 3.11 | 0.023832 | 0.952769 | 0.012307 | 0.011092 | 0.083228 |
| 2.98 | 0.056379 | 0.943621 | 0 | 0 | 0.123161 |
| 2.98 | 0.031151 | 0.746825 | 0.203347 | 0.018677 | 0.101497 |
| 2.83 | 0.043831 | 0.54447 | 0.298957 | 0.112741 | 0.002629 |
| 2.82 | 0.108022 | 0.691244 | 0.180332 | 0.020402 | 0.004315 |
| 2.7 | 0.027806 | 0.664197 | 0.305735 | 0.002262 | 0.027812 |
| 2.67 | 0.068708 | 0.849552 | 0.042768 | 0.038973 | 0.074069 |
| 2.63 | 0.074414 | 0.869734 | 0.019153 | 0.036699 | 0.003266 |
| 2.55 | 0.036539 | 0.951535 | 0.011926 | 0 | 0.025928 |
| 2.44 | 0.040779 | 0.952662 | 0 | 0.006559 | 0.231493 |
| 2.44 | 0.04777 | 0.769868 | 0.182362 | 0 | 0.025439 |
| 2.15 | 0.050098 | 0.942054 | 0.007554 | 0.000294 | 0.032512 |
| 2.04 | 0.044407 | 0.778417 | 0.176709 | 0.000467 | 0.032712 |
| 2.02 | 0.011622 | 0.828127 | 0.160251 | 0 | 0.020163 |
| 1.88 | 0.0599 | 0.70447 | 0.203372 | 0.032257 | 0.035467 |
| 1.87 | 0.038505 | 0.828164 | 0.133331 | 0 | 0.114387 |
| 1.75 | 0.039787 | 0.928108 | 0.016795 | 0.015311 | 0.04215 |
| 1.66 | 0.03865 | 0.910378 | 0.050972 | 0 | 0.003528 |
| 1.57 | 0.02633 | 0.705616 | 0.268053 | 0 | 3.04E-06 |
| 1.44 | 0.008458 | 0.766388 | 0.13723 | 0.087924 | 0.073515 |
| 1.34 | 0.052039 | 0.626445 | 0.321516 | 0 | 0.038994 |
| 1.1 | 0.012567 | 0.970659 | 0.016774 | 0 | 0.011678 |
| 1.04 | 0.046376 | 0.702486 | 0.251138 | 0 | 0.003461 |
| 1.04 | 0.002975 | 0.947564 | 0.037218 | 0.012244 | 0.071134 |
| 0.96 | 0.027594 | 0.755788 | 0.216618 | 0 | 0.07257 |
| 0.94 | 0.11287 | 0.864337 | 0.022793 | 0 | 0.094936 |
| 0.64 | 0.006401 | 0.762863 | 0.215353 | 0.015384 | 0.120429 |

*Wild boar*

| Harvest  (ind./100ha) | SETTLEMENTS  (%) | CROPS  (%) | FORESTS  (%) | WATER  (%) | WIND FARM  (%) |
| --- | --- | --- | --- | --- | --- |
| 43.52 | 0.134499 | 0.702936 | 0.162566 | 0 | 0.002641 |
| 40.79 | 0.130716 | 0.674756 | 0.194527 | 0 | 0.138547 |
| 37.3 | 0.139615 | 0.679458 | 0.17389 | 0.007037 | 0.000136 |
| 34.65 | 0.042009 | 0.716236 | 0.241755 | 0 | 0.25503 |
| 34.05 | 0.021317 | 0.804667 | 0.174016 | 0 | 0.278978 |
| 33.61 | 0.032579 | 0.759046 | 0.200145 | 0.008229 | 0.014697 |
| 31.37 | 0.029013 | 0.883584 | 0.082962 | 0.004441 | 0.000183 |
| 31.34 | 0.095858 | 0.675914 | 0.223198 | 0.005031 | 0.017825 |
| 31.06 | 0.034773 | 0.709889 | 0.247028 | 0.008309 | 0.059819 |
| 30.98 | 0.112847 | 0.540884 | 0.2825 | 0.063769 | 0.234756 |
| 30.41 | 0.049178 | 0.932341 | 0.018482 | 0 | 0.034648 |
| 30.05 | 0.098187 | 0.786894 | 0.114919 | 0 | 0.033695 |
| 29.2 | 0.025726 | 0.568486 | 0.358865 | 0.046922 | 0.138587 |
| 28.95 | 0.101064 | 0.634403 | 0.264178 | 0.000356 | 0.002776 |
| 28.13 | 0.012828 | 0.801466 | 0.169907 | 0.015799 | 0.035618 |
| 25.63 | 0.124811 | 0.814342 | 0.060847 | 0 | 0.105066 |
| 25.33 | 0.035057 | 0.565328 | 0.393756 | 0.005859 | 0.098151 |
| 24.48 | 0.029447 | 0.903798 | 0.066755 | 0 | 0.23376 |
| 24.3 | 0.012081 | 0.564216 | 0.390193 | 0.03351 | 0.035279 |
| 23.76 | 0.070667 | 0.90593 | 0.02212 | 0.001283 | 0.083461 |
| 23.59 | 0.086829 | 0.552521 | 0.36065 | 0 | 0.123484 |
| 23.36 | 0.081238 | 0.718524 | 0.200238 | 0 | 0.013759 |
| 23.34 | 0.04376 | 0.936753 | 0.019487 | 0 | 0.061764 |
| 22.27 | 0.017442 | 0.520835 | 0.389462 | 0.072261 | 0.001443 |
| 22.22 | 0.077148 | 0.72609 | 0.196763 | 0 | 0.022359 |
| 22.04 | 0.025165 | 0.709589 | 0.265246 | 0 | 0.340679 |
| 21.41 | 0.052708 | 0.717515 | 0.2169 | 0.012876 | 0.308219 |
| 21.06 | 0.0274 | 0.680913 | 0.280471 | 0.011216 | 0.088547 |
| 20.82 | 0.067136 | 0.715851 | 0.217013 | 0 | 0.03475 |
| 20.8 | 0.02159 | 0.743128 | 0.235281 | 0 | 0.058284 |
| 20.62 | 0.077519 | 0.685448 | 0.237032 | 0 | 0.018202 |
| 20.18 | 0.028701 | 0.880606 | 0.090693 | 0 | 0.1192 |
| 20.02 | 0.137764 | 0.692755 | 0.169481 | 0 | 0.01181 |
| 19.72 | 0.035474 | 0.794716 | 0.16981 | 0 | 0.222865 |
| 19.5 | 0.075708 | 0.831813 | 0.092479 | 0 | 0.125231 |
| 19.4 | 0.022756 | 0.827933 | 0.149312 | 0 | 0.036017 |
| 19.32 | 0.030907 | 0.705282 | 0.26381 | 0 | 0.210419 |
| 19.04 | 0.032721 | 0.728697 | 0.238522 | 5.97E-05 | 0.018052 |
| 18.79 | 0.07946 | 0.663096 | 0.257444 | 0 | 0.119837 |
| 18.74 | 0.066768 | 0.576755 | 0.179528 | 0.176949 | 0.046495 |
| 18.56 | 0.098285 | 0.880774 | 0.020941 | 0 | 0.173938 |
| 18.55 | 0.033665 | 0.827557 | 0.095811 | 0.042966 | 0.085835 |
| 18.49 | 0.046989 | 0.861338 | 0.091672 | 0 | 0.288311 |
| 18.3 | 0.142246 | 0.407022 | 0.377298 | 0.073434 | 0.089493 |
| 17.88 | 0.032981 | 0.830037 | 0.110184 | 0.026798 | 0.145802 |
| 17.74 | 0.041239 | 0.678391 | 0.280371 | 0 | 0.166558 |
| 17.57 | 0.003095 | 0.950925 | 0.015182 | 0.030799 | 0.103433 |
| 17.44 | 0.032845 | 0.784505 | 0.155122 | 0.027527 | 0.09242 |
| 17.42 | 0.022113 | 0.740767 | 0.237121 | 0 | 0.271627 |
| 17.35 | 0.048241 | 0.890315 | 0.061444 | 0 | 0.004877 |
| 17.24 | 0.103313 | 0.732831 | 0.163856 | 0 | 0.045637 |
| 17.12 | 0.018482 | 0.571366 | 0.403494 | 0.006658 | 0.08103 |
| 17.1 | 0.011447 | 0.645638 | 0.323308 | 0.019607 | 0.001809 |
| 17.02 | 0.072147 | 0.370505 | 0.269155 | 0.273316 | 0.041596 |
| 16.92 | 0.033849 | 0.683169 | 0.28066 | 0.002321 | 0.049347 |
| 16.66 | 0.039215 | 0.907213 | 0.053573 | 0 | 0.070329 |
| 16.47 | 0.100035 | 0.878576 | 0 | 0.021389 | 0.004507 |
| 16.25 | 0.071109 | 0.886488 | 0.042403 | 0 | 0.027537 |
| 16.2 | 0.026162 | 0.710156 | 0.240745 | 0.022937 | 0.031545 |
| 16.08 | 0.058012 | 0.561444 | 0.380544 | 0 | 0.028502 |
| 16 | 0.040076 | 0.833117 | 0.087333 | 0.039474 | 0.241711 |
| 15.79 | 0.12475 | 0.506296 | 0.368954 | 0 | 0.018843 |
| 15.58 | 0.048955 | 0.647511 | 0.303534 | 0 | 0.045488 |
| 15.43 | 0.017962 | 0.804942 | 0.163669 | 0.013427 | 0.006701 |
| 15.2 | 0.032367 | 0.938775 | 0.019579 | 0.009278 | 0.008665 |
| 15.03 | 0.033553 | 0.805444 | 0.155166 | 0.005836 | 0.089808 |
| 14.94 | 0.018303 | 0.841083 | 0.134267 | 0.006347 | 0.007471 |
| 14.91 | 0.052284 | 0.743996 | 0.193735 | 0.009985 | 0.045387 |
| 14.88 | 0.043952 | 0.822823 | 0.084006 | 0.049219 | 0.044707 |
| 14.62 | 0.031001 | 0.661591 | 0.277932 | 0.029476 | 0.000647 |
| 14.6 | 0.054088 | 0.749397 | 0.163075 | 0.03344 | 0.116868 |
| 14.12 | 0.021323 | 0.624195 | 0.354482 | 0 | 0.002746 |
| 13.98 | 0.045204 | 0.596497 | 0.333565 | 0.01242 | 0.118649 |
| 13.89 | 0.028499 | 0.562559 | 0.360137 | 0.048805 | 0.001166 |
| 13.83 | 0.072848 | 0.708754 | 0.212019 | 0.006379 | 0.2346 |
| 13.69 | 0.028708 | 0.609643 | 0.361648 | 0 | 0.082055 |
| 13.67 | 0.037805 | 0.73086 | 0.231335 | 0 | 0.156062 |
| 13.44 | 0.119335 | 0.541145 | 0.336507 | 0.003013 | 0.018434 |
| 13.42 | 0.032002 | 0.781137 | 0.152364 | 0.034498 | 0.000807 |
| 13.09 | 0.078413 | 0.768356 | 0.153232 | 0 | 0.086105 |
| 12.88 | 0.017755 | 0.694353 | 0.28756 | 0.000331 | 0.007311 |
| 12.79 | 0.034684 | 0.90422 | 0.061095 | 0 | 0.079926 |
| 12.73 | 0.04601 | 0.785236 | 0.14245 | 0.026304 | 0.131842 |
| 12.35 | 0.043989 | 0.711189 | 0.244822 | 0 | 0.171178 |
| 12.25 | 0.041492 | 0.83554 | 0.122968 | 0 | 0.236861 |
| 12.1 | 0.04838 | 0.768229 | 0.137646 | 0.045745 | 0.000874 |
| 11.94 | 0.086482 | 0.913518 | 0 | 0 | 0.005667 |
| 11.84 | 0.016859 | 0.887013 | 0.096128 | 0 | 0.07925 |
| 11.82 | 0.036625 | 0.770464 | 0.167258 | 0.025652 | 0.178928 |
| 11.74 | 0.02633 | 0.705616 | 0.268053 | 0 | 3.04E-06 |
| 11.72 | 0.077189 | 0.793972 | 0.122556 | 0.006282 | 0.067732 |
| 11.67 | 0.039 | 0.770268 | 0.167469 | 0.023263 | 0.142616 |
| 11.57 | 0.12639 | 0.853682 | 0.019928 | 0 | 0.018693 |
| 11.57 | 0.059836 | 0.773228 | 0.059752 | 0.107184 | 0.135669 |
| 11.55 | 0.049416 | 0.637355 | 0.298827 | 0.014403 | 0.051553 |
| 11.47 | 0.010053 | 0.727504 | 0.207121 | 0.055322 | 0.048494 |
| 11.39 | 0.502659 | 0.453471 | 0.003471 | 0.040398 | 0.154066 |
| 11.38 | 0.039841 | 0.816561 | 0.143598 | 0 | 0.101836 |
| 11.38 | 0.01221 | 0.707236 | 0.280555 | 0 | 0.010554 |
| 11.37 | 0.024747 | 0.964457 | 0.010796 | 0 | 0.256071 |
| 11.28 | 0.029925 | 0.788213 | 0.181862 | 0 | 0.020835 |
| 11.27 | 0.09225 | 0.835858 | 0.071892 | 0 | 0.064066 |
| 11.24 | 0.046144 | 0.680903 | 0.254461 | 0.018493 | 0.000879 |
| 11.2 | 0.034088 | 0.734203 | 0.231709 | 0 | 0.003313 |
| 11.04 | 0.026258 | 0.530627 | 0.436197 | 0.006918 | 0.028396 |
| 10.98 | 0.040323 | 0.76684 | 0.192836 | 0 | 0.016819 |
| 10.96 | 0.032369 | 0.54685 | 0.415187 | 0.005593 | 0.023739 |
| 10.7 | 0.032119 | 0.666449 | 0.28536 | 0.016073 | 0.089523 |
| 10.56 | 0.029683 | 0.738291 | 0.186006 | 0.046019 | 0.017025 |
| 10.48 | 0.030385 | 0.902018 | 0.026566 | 0.041031 | 0.017606 |
| 10.37 | 0.151948 | 0.530499 | 0.120228 | 0.197325 | 0.019356 |
| 10.33 | 0.069932 | 0.770161 | 0.159907 | 0 | 0.300129 |
| 10.32 | 0.012225 | 0.952622 | 0.035153 | 0 | 0.01266 |
| 10.27 | 0.028579 | 0.779842 | 0.184295 | 0.007283 | 0.00428 |
| 10.03 | 0.08025 | 0.857246 | 0.062497 | 7.34E-06 | 0.108955 |
| 10 | 0.03957 | 0.723527 | 0.232789 | 0.004115 | 0.000244 |
| 9.96 | 0.027594 | 0.755788 | 0.216618 | 0 | 0.07257 |
| 9.91 | 0.011822 | 0.922221 | 0.0577 | 0.008256 | 0.027789 |
| 9.91 | 0.025498 | 0.828399 | 0.114062 | 0.032041 | 0.078826 |
| 9.87 | 0.024205 | 0.53313 | 0.202177 | 0.240487 | 0.002058 |
| 9.79 | 0.045934 | 0.924793 | 0.02209 | 0.007184 | 0.19778 |
| 9.79 | 0.21499 | 0.702711 | 0.080053 | 0.002246 | 0.007246 |
| 9.75 | 0.177178 | 0.698147 | 0.059042 | 0.065633 | 0.169988 |
| 9.73 | 0.060716 | 0.682299 | 0.256985 | 0 | 0.053094 |
| 9.64 | 0.019528 | 0.767714 | 0.212758 | 0 | 0.030766 |
| 9.48 | 0.137039 | 0.844241 | 0.01872 | 0 | 0.054031 |
| 9.42 | 0.033275 | 0.900866 | 0.060343 | 0.005515 | 0.091831 |
| 9.4 | 0.027087 | 0.892398 | 0.06958 | 0.010935 | 0.015594 |
| 9.32 | 0.064382 | 0.647883 | 0.274131 | 0.013604 | 0.054466 |
| 9.21 | 0.01543 | 0.653371 | 0.299888 | 0.031312 | 0.052174 |
| 9.2 | 0.036346 | 0.809759 | 0.128229 | 0.025666 | 0.037496 |
| 9.14 | 0.053113 | 0.649051 | 0.261498 | 0.036338 | 0.042373 |
| 9.08 | 0.041854 | 0.859523 | 0.094217 | 0.004407 | 0.152709 |
| 9.07 | 0.028999 | 0.817861 | 0.15314 | 0 | 0.078066 |
| 9 | 0.051785 | 0.901516 | 0.046699 | 0 | 0.007171 |
| 8.93 | 0.012941 | 0.611311 | 0.36866 | 0.007087 | 0.050402 |
| 8.91 | 0.012158 | 0.877799 | 0.104953 | 0.00509 | 0.119091 |
| 8.87 | 0.092472 | 0.841024 | 0.066505 | 0 | 0.094696 |
| 8.77 | 0.065776 | 0.916671 | 0.017553 | 0 | 0.021232 |
| 8.76 | 0.043831 | 0.54447 | 0.298957 | 0.112741 | 0.002629 |
| 8.72 | 0.065773 | 0.829725 | 0.103836 | 0.000666 | 0.181817 |
| 8.72 | 0.01259 | 0.695308 | 0.274294 | 0.017807 | 0.002173 |
| 8.69 | 0.072476 | 0.909047 | 0.018477 | 0 | 0.060021 |
| 8.57 | 0.003406 | 0.599878 | 0.396716 | 0 | 0.045399 |
| 8.53 | 0.059243 | 0.805019 | 0.135738 | 0 | 0.094313 |
| 8.53 | 0.042169 | 0.597119 | 0.360712 | 0 | 0.036652 |
| 8.49 | 0.041354 | 0.793022 | 0.109663 | 0.055961 | 0.156689 |
| 8.4 | 0.02038 | 0.918323 | 0.036001 | 0.025295 | 0.032543 |
| 8.36 | 0.029175 | 0.877794 | 0.093031 | 0 | 0.015471 |
| 8.2 | 0.050548 | 0.895549 | 0.053477 | 0.000426 | 0.040513 |
| 8.18 | 0.04557 | 0.58709 | 0.36734 | 0 | 0.070527 |
| 8.14 | 0.096755 | 0.818844 | 0.082158 | 0.002244 | 0.031972 |
| 8.07 | 0.01756 | 0.936756 | 0.045684 | 0 | 0.587545 |
| 8.03 | 0.001377 | 0.682013 | 0.307943 | 0.008666 | 0.024811 |
| 7.97 | 0.06371 | 0.687375 | 0.248915 | 0 | 0.024296 |
| 7.96 | 0.077906 | 0.776797 | 0.145296 | 0 | 0.026626 |
| 7.95 | 0.049931 | 0.628573 | 0.321228 | 0.000269 | 0.036331 |
| 7.93 | 0.077374 | 0.85315 | 0.00706 | 0.062416 | 0.027737 |
| 7.89 | 0.03717 | 0.760903 | 0.201927 | 0 | 0.006919 |
| 7.88 | 0.034667 | 0.836308 | 0.12772 | 0.001305 | 0.026518 |
| 7.85 | 0.058187 | 0.528333 | 0.413479 | 0 | 0.002676 |
| 7.81 | 0.051831 | 0.828658 | 0.11951 | 0 | 0.107904 |
| 7.76 | 0.030872 | 0.686417 | 0.272097 | 0.010614 | 0.010509 |
| 7.57 | 0.092208 | 0.70089 | 0.190913 | 0.015988 | 0.053543 |
| 7.55 | 0.01712 | 0.800569 | 0.132823 | 0.049489 | 0.06699 |
| 7.54 | 0.035595 | 0.757742 | 0.206663 | 0 | 0.026593 |
| 7.53 | 0.062328 | 0.768301 | 0.146098 | 0.023273 | 0.041255 |
| 7.42 | 0.041745 | 0.712863 | 0.243395 | 0.001997 | 0.02306 |
| 7.42 | 0.105751 | 0.72069 | 0.166077 | 0.007481 | 0.034231 |
| 7.34 | 0.013851 | 0.854947 | 0.094772 | 0.036431 | 0.005141 |
| 7.31 | 0.049629 | 0.944396 | 0.005975 | 0 | 0.171729 |
| 7.26 | 0.037255 | 0.909058 | 0.053687 | 0 | 0.110157 |
| 7.17 | 0.102823 | 0.881072 | 0.016105 | 0 | 0.048898 |
| 7.16 | 0.053764 | 0.773062 | 0.173173 | 0 | 0.151376 |
| 7.14 | 0.031111 | 0.552667 | 0.394879 | 0.021342 | 0.026238 |
| 7.09 | 0.041595 | 0.871269 | 0.087137 | 0 | 0.085193 |
| 7.06 | 0.038604 | 0.954512 | 0.006884 | 0 | 0.02608 |
| 7.05 | 0.029025 | 0.753063 | 0.182242 | 0.03567 | 0.000693 |
| 7.02 | 0.203607 | 0.526209 | 0.270184 | 0 | 0.050672 |
| 7.01 | 0.029603 | 0.751571 | 0.218827 | 0 | 0.079736 |
| 6.95 | 0.036312 | 0.904341 | 0.024842 | 0.034504 | 0.160208 |
| 6.92 | 0.025682 | 0.856233 | 0.069107 | 0.048978 | 0.562058 |
| 6.88 | 0.030802 | 0.910639 | 0.052474 | 0.006085 | 0.34264 |
| 6.87 | 0.105629 | 0.788264 | 0.072893 | 0.033215 | 0.044414 |
| 6.86 | 0.015465 | 0.974209 | 0.010326 | 0 | 0.001597 |
| 6.85 | 0.007743 | 0.882418 | 0.104562 | 0.005277 | 0.030295 |
| 6.84 | 0.102583 | 0.897417 | 0 | 0 | 0.045341 |
| 6.72 | 0.059391 | 0.887374 | 0.052975 | 0.00026 | 0.002722 |
| 6.68 | 0.073048 | 0.7756 | 0.151352 | 0 | 0.061654 |
| 6.56 | 0.065419 | 0.70728 | 0.227301 | 0 | 0.288139 |
| 6.55 | 0.074589 | 0.640797 | 0.26404 | 0.020574 | 0.021459 |
| 6.47 | 0.182413 | 0.806462 | 0.011125 | 0 | 0.037714 |
| 6.47 | 0.039022 | 0.789893 | 0.171085 | 0 | 0.011916 |
| 6.46 | 0.072737 | 0.703643 | 0.172948 | 0.050672 | 0.003121 |
| 6.44 | 0 | 0.891838 | 0.108162 | 0 | 0.002346 |
| 6.42 | 0.042539 | 0.616348 | 0.341113 | 0 | 0.050441 |
| 6.41 | 0.039287 | 0.659343 | 0.239964 | 0.061407 | 0.021402 |
| 6.27 | 0.095985 | 0.700639 | 0.198975 | 0.0044 | 0.02055 |
| 6.19 | 0.108214 | 0.736279 | 0.126343 | 0.029164 | 0.000839 |
| 6.17 | 0.080689 | 0.873851 | 0.041035 | 0.004425 | 0.180591 |
| 6.14 | 0.046888 | 0.885726 | 0.036393 | 0.030993 | 0.012365 |
| 6.14 | 0.039787 | 0.928108 | 0.016795 | 0.015311 | 0.04215 |
| 6.11 | 0.041847 | 0.933975 | 0 | 0.024178 | 0.027727 |
| 6.1 | 0.040779 | 0.952662 | 0 | 0.006559 | 0.231493 |
| 6.07 | 0.065527 | 0.728519 | 0.126969 | 0.078986 | 0.047136 |
| 6.02 | 0.016972 | 0.707628 | 0.225538 | 0.049862 | 0.111867 |
| 5.9 | 0.064454 | 0.807537 | 0.118761 | 0.009248 | 0.039809 |
| 5.9 | 0.096224 | 0.839476 | 0.0643 | 0 | 0.055361 |
| 5.85 | 0.111642 | 0.775675 | 0.112682 | 0 | 0.047805 |
| 5.71 | 0.043339 | 0.663185 | 0.278789 | 0.014687 | 0.102922 |
| 5.68 | 0.010139 | 0.929534 | 0.039062 | 0.021265 | 0.02452 |
| 5.64 | 0.046194 | 0.608006 | 0.345294 | 0.000506 | 0.002681 |
| 5.64 | 0.072977 | 0.856731 | 0.070292 | 0 | 0.074098 |
| 5.62 | 0.038505 | 0.828164 | 0.133331 | 0 | 0.114387 |
| 5.62 | 0.023913 | 0.904383 | 0.050222 | 0.021483 | 0.365279 |
| 5.57 | 0.080504 | 0.831155 | 0.005685 | 0.082657 | 0.041065 |
| 5.49 | 0.029573 | 0.84438 | 0.121215 | 0.004832 | 0.031662 |
| 5.49 | 0.072839 | 0.741172 | 0.185989 | 0 | 0.02913 |
| 5.44 | 0.008032 | 0.962155 | 0.004333 | 0.02548 | 0.026076 |
| 5.43 | 0.070103 | 0.80001 | 0.098141 | 0.031745 | 0.019618 |
| 5.41 | 0.076638 | 0.893076 | 0.030286 | 0 | 0.003956 |
| 5.35 | 0.020858 | 0.994368 | 0.0019 | 0 | 0.003386 |
| 5.31 | 0.055185 | 0.873829 | 0.070986 | 0 | 0.060068 |
| 5.27 | 0.039739 | 0.691329 | 0.268932 | 0 | 0.195716 |
| 5.21 | 0.035856 | 0.89092 | 0.073224 | 0 | 0.152419 |
| 5.19 | 0.141596 | 0.638012 | 0.156134 | 0.064258 | 0.108228 |
| 5.17 | 0.023363 | 0.846999 | 0.129638 | 0 | 0.078641 |
| 5.16 | 0.108022 | 0.691244 | 0.180332 | 0.020402 | 0.004315 |
| 5.14 | 0.009447 | 0.761519 | 0.21221 | 0.016824 | 0.01016 |
| 5.1 | 0.055696 | 0.879582 | 0.060887 | 0.003835 | 0.021538 |
| 5.07 | 0.053881 | 0.915746 | 0.006846 | 0.023526 | 0.034166 |
| 5.06 | 0.043407 | 0.941455 | 0.015138 | 0 | 0.002294 |
| 5.05 | 0.091309 | 0.876741 | 0.03195 | 0 | 0.02242 |
| 5.03 | 0.021547 | 0.897054 | 0.081399 | 0 | 0.003304 |
| 5.02 | 0.023883 | 0.761086 | 0.215031 | 0 | 0.033082 |
| 5.02 | 0.142331 | 0.782279 | 0.058797 | 0.016592 | 0.041235 |
| 4.99 | 0.016053 | 0.877654 | 0.080995 | 0.025299 | 0.022378 |
| 4.89 | 0.01512 | 0.858559 | 0.07309 | 0.053231 | 0.037677 |
| 4.88 | 0.08957 | 0.6941 | 0.21633 | 0 | 0.055495 |
| 4.88 | 0.0432 | 0.9568 | 0 | 0 | 0.013619 |
| 4.87 | 0.036829 | 0.673528 | 0.283352 | 0.006291 | 0.031069 |
| 4.81 | 0.063612 | 0.891342 | 0.045046 | 0 | 0.107764 |
| 4.8 | 0.279126 | 0.620004 | 0.100869 | 0 | 0.032547 |
| 4.8 | 0.037227 | 0.774049 | 0.188724 | 0 | 0.136711 |
| 4.79 | 0.134972 | 0.724281 | 0.120111 | 0.020636 | 0.008062 |
| 4.74 | 0.020053 | 0.595764 | 0.384183 | 0 | 0.022035 |
| 4.71 | 0.110639 | 0.740989 | 0.108626 | 0.039746 | 0.079146 |
| 4.66 | 0.073033 | 0.813903 | 0.097747 | 0.015317 | 0.19531 |
| 4.63 | 0.037281 | 0.903436 | 0.042729 | 0.016554 | 0.047312 |
| 4.63 | 0.011622 | 0.828127 | 0.160251 | 0 | 0.020163 |
| 4.6 | 0.077451 | 0.901627 | 0.020923 | 0 | 0.037246 |
| 4.57 | 0.058021 | 0.662699 | 0.258411 | 0.020869 | 0.038288 |
| 4.57 | 0.045885 | 0.95365 | 0.000465 | 0 | 0.003323 |
| 4.55 | 0.020372 | 0.836787 | 0.071528 | 0.071313 | 0.01532 |
| 4.53 | 0.054568 | 0.700053 | 0.236636 | 0.008742 | 0.051978 |
| 4.52 | 0.02837 | 0.674304 | 0.282227 | 0.015099 | 0.162311 |
| 4.52 | 0.027274 | 0.638686 | 0.329299 | 0.004741 | 0.087119 |
| 4.5 | 0.059068 | 0.905269 | 0.035663 | 0 | 0.061176 |
| 4.47 | 0.02551 | 0.914172 | 0.045998 | 0.01432 | 0.02754 |
| 4.46 | 0.05913 | 0.575212 | 0.365601 | 5.72E-05 | 0.03847 |
| 4.45 | 0.02289 | 0.955775 | 0.021335 | 0 | 0.120855 |
| 4.44 | 0.068708 | 0.849552 | 0.042768 | 0.038973 | 0.074069 |
| 4.42 | 0.067538 | 0.901478 | 0.024953 | 0.00603 | 0.018914 |
| 4.39 | 0.074807 | 0.798245 | 0.126948 | 0 | 0.052297 |
| 4.35 | 0.086936 | 0.888209 | 0.024855 | 0 | 0.080122 |
| 4.33 | 0.05676 | 0.713201 | 0.23004 | 0 | 0.025905 |
| 4.3 | 0.180694 | 0.813429 | 0 | 0.005877 | 0.020059 |
| 4.29 | 0.04306 | 0.876667 | 0.080273 | 0 | 0.034083 |
| 4.28 | 0.060899 | 0.775398 | 0.163702 | 0 | 0.022238 |
| 4.25 | 0.048003 | 0.729414 | 0.209387 | 0.013197 | 0.033985 |
| 4.22 | 0.016337 | 0.866699 | 0.111594 | 0.00537 | 0.013295 |
| 4.2 | 0.043956 | 0.854424 | 0.090371 | 0.011248 | 0.042874 |
| 4.18 | 0.083189 | 0.898119 | 0.012762 | 0.00593 | 0.16835 |
| 4.18 | 0.046884 | 0.923363 | 0.029753 | 0 | 0.00521 |
| 4.17 | 0.04377 | 0.927657 | 0.028573 | 0 | 0.115429 |
| 4.15 | 0.050611 | 0.799196 | 0.130295 | 0.019897 | 0.045145 |
| 4.14 | 0.041867 | 0.696324 | 0.261809 | 0 | 0.009857 |
| 4.11 | 0.070047 | 0.841396 | 0.083412 | 0.005146 | 0.038956 |
| 4.06 | 0.046235 | 0.768727 | 0.185038 | 0 | 0.003722 |
| 4.04 | 0.03637 | 0.8723 | 0.091329 | 0 | 0.000179 |
| 4.01 | 0.046998 | 0.826689 | 0.126312 | 0 | 0.087959 |
| 3.99 | 0.067705 | 0.60951 | 0.322784 | 0 | 0.031049 |
| 3.93 | 0.03372 | 0.954883 | 0.011397 | 0 | 0.059214 |
| 3.91 | 0.167614 | 0.459503 | 0.216179 | 0.156704 | 0.034594 |
| 3.85 | 0.029173 | 0.847523 | 0.123304 | 0 | 0.02368 |
| 3.82 | 0.041801 | 0.944368 | 0 | 0.013831 | 0.010372 |
| 3.73 | 0.023832 | 0.952769 | 0.012307 | 0.011092 | 0.083228 |
| 3.66 | 0.027512 | 0.92664 | 0.024607 | 0.02124 | 0.166807 |
| 3.52 | 0.048432 | 0.813801 | 0.137767 | 0 | 0.016043 |
| 3.51 | 0.029263 | 0.64246 | 0.263832 | 0.064444 | 0.068015 |
| 3.47 | 0.036485 | 0.81515 | 0.148365 | 0 | 0.19155 |
| 3.46 | 0.019597 | 0.789053 | 0.19135 | 0 | 0.007049 |
| 3.45 | 0.015311 | 0.850012 | 0.134677 | 0 | 0.043252 |
| 3.41 | 0.019231 | 0.918595 | 0.062174 | 0 | 0.02441 |
| 3.38 | 0.01082 | 0.979523 | 0 | 0.009656 | 0.210532 |
| 3.37 | 0.078715 | 0.778306 | 0.133458 | 0.009521 | 0.022315 |
| 3.37 | 0.079122 | 0.728452 | 0.189573 | 0.002853 | 0.013886 |
| 3.37 | 0.030193 | 0.919865 | 0.019158 | 0.030784 | 0.093644 |
| 3.34 | 0.08742 | 0.91258 | 0 | 0 | 0.040064 |
| 3.27 | 0.044407 | 0.778417 | 0.176709 | 0.000467 | 0.032712 |
| 3.19 | 0.11188 | 0.782154 | 0.105966 | 0 | 0.001959 |
| 3.13 | 0.010679 | 0.952 | 0.001661 | 0.035661 | 0.004639 |
| 3.12 | 0.027717 | 0.596513 | 0.370443 | 0.005327 | 0.035165 |
| 3.12 | 0.042068 | 0.913719 | 0.035636 | 0.008577 | 0.070134 |
| 3.11 | 0.071363 | 0.916463 | 0.012174 | 0 | 0.181614 |
| 3.05 | 0.130492 | 0.869508 | 0 | 0 | 0.036249 |
| 3.05 | 0.034103 | 0.818442 | 0.147454 | 0 | 0.087954 |
| 3.03 | 0.028556 | 0.832744 | 0.1387 | 0 | 0.027699 |
| 2.97 | 0.061675 | 0.89241 | 0.045559 | 0.000356 | 0.165958 |
| 2.96 | 0.018798 | 0.873342 | 0.096714 | 0.011147 | 0.000615 |
| 2.96 | 0.063263 | 0.772515 | 0.155506 | 0.008716 | 0.17232 |
| 2.95 | 0.069296 | 0.774471 | 0.070706 | 0.085528 | 0.031938 |
| 2.95 | 0.017478 | 0.896971 | 0.020908 | 0.064643 | 0.124726 |
| 2.95 | 0.012878 | 0.947298 | 0.03675 | 0.003074 | 0.028568 |
| 2.95 | 0.03309 | 0.827144 | 0.139765 | 0 | 0.030663 |
| 2.91 | 0.060047 | 0.690705 | 0.249248 | 0 | 0.327264 |
| 2.87 | 0.036113 | 0.84386 | 0.028627 | 0.0914 | 0.01802 |
| 2.87 | 0.07399 | 0.807539 | 0.022472 | 0.096 | 0.028334 |
| 2.85 | 0.04474 | 0.643511 | 0.311749 | 0 | 0.033318 |
| 2.84 | 0.032047 | 0.620483 | 0.34747 | 0 | 0.032438 |
| 2.84 | 0.045414 | 0.745517 | 0.195632 | 0.013437 | 0.031831 |
| 2.82 | 0.0599 | 0.70447 | 0.203372 | 0.032257 | 0.035467 |
| 2.81 | 0.020796 | 0.921761 | 0.057443 | 0 | 0.011385 |
| 2.79 | 0.009634 | 0.907754 | 0.082612 | 0 | 0.016301 |
| 2.78 | 0.025748 | 0.720481 | 0.253771 | 0 | 0.036214 |
| 2.78 | 0.040823 | 0.959177 | 0 | 0 | 0.004214 |
| 2.77 | 0.069289 | 0.841813 | 0.088899 | 0 | 0.074837 |
| 2.76 | 0.085555 | 0.904837 | 0.009608 | 0 | 0.034194 |
| 2.74 | 0.041876 | 0.634991 | 0.323132 | 0 | 0.019803 |
| 2.71 | 0.224025 | 0.744055 | 0.03192 | 0 | 0.080825 |
| 2.68 | 0.021306 | 0.96547 | 0.000434 | 0.01279 | 0.093825 |
| 2.68 | 0.114896 | 0.770582 | 0.111487 | 0.003035 | 0.057439 |
| 2.67 | 0.031613 | 0.961818 | 0.003158 | 0.003411 | 0.046069 |
| 2.67 | 0.035743 | 0.671162 | 0.284171 | 0.008924 | 0.015164 |
| 2.67 | 0.025413 | 0.787728 | 0.186858 | 0 | 0.021162 |
| 2.66 | 0.005309 | 0.943367 | 0.02594 | 0.025384 | 0.000813 |
| 2.65 | 0.036323 | 0.95832 | 0.005356 | 0 | 0.348035 |
| 2.64 | 0.030564 | 0.913953 | 0.055484 | 0 | 0.087083 |
| 2.64 | 0.01264 | 0.937165 | 0.043062 | 0.007133 | 0.185625 |
| 2.59 | 0.014978 | 0.876519 | 0.099945 | 0.008559 | 0.01608 |
| 2.59 | 0.157547 | 0.729757 | 0.10907 | 0.003625 | 0.027969 |
| 2.58 | 0.034493 | 0.812457 | 0.153051 | 0 | 0.073334 |
| 2.57 | 0.051852 | 0.630668 | 0.304929 | 0.01255 | 0.049537 |
| 2.54 | 0.061571 | 0.728748 | 0.209681 | 0 | 0.041562 |
| 2.51 | 0.020569 | 0.771368 | 0.155748 | 0.052315 | 0.015713 |
| 2.49 | 0.009555 | 0.979002 | 0.000198 | 0.011245 | 0.076538 |
| 2.49 | 0.014883 | 0.952852 | 0.026053 | 0.006211 | 0.039717 |
| 2.45 | 0.02225 | 0.900537 | 0.067932 | 0.00928 | 0.000577 |
| 2.45 | 0.007157 | 0.760099 | 0.232744 | 0 | 0.138899 |
| 2.45 | 0.139056 | 0.729618 | 0.11691 | 0.014416 | 2.54E-05 |
| 2.41 | 0.049055 | 0.900592 | 0.050353 | 0 | 0.099 |
| 2.4 | 0.077048 | 0.723402 | 0.19955 | 0 | 0.077261 |
| 2.31 | 0.018063 | 0.899386 | 0.044394 | 0.038157 | 0.085235 |
| 2.3 | 0.06005 | 0.93995 | 0 | 0 | 0.023202 |
| 2.29 | 0.033544 | 0.839022 | 0.127434 | 0 | 0.062529 |
| 2.28 | 0.032947 | 0.773658 | 0.145192 | 0.048202 | 0.049896 |
| 2.25 | 0.004494 | 0.931113 | 0.025152 | 0.03924 | 0.009948 |
| 2.25 | 0.066093 | 0.856123 | 0.077784 | 0 | 0.032206 |
| 2.25 | 0.026381 | 0.950107 | 0 | 0.023512 | 0.343647 |
| 2.24 | 0.039445 | 0.880548 | 0.078509 | 0.001499 | 0.00146 |
| 2.23 | 0.064139 | 0.813701 | 0.122161 | 0 | 0.111806 |
| 2.23 | 0.020504 | 0.893617 | 0.085879 | 0 | 0.028256 |
| 2.16 | 0.019281 | 0.635579 | 0.264739 | 0.080401 | 0.007959 |
| 2.13 | 0.012366 | 0.881566 | 0.053689 | 0.052379 | 0.104641 |
| 2.11 | 0.045738 | 0.828349 | 0.125913 | 0 | 0.022493 |
| 2.09 | 0.053152 | 0.914463 | 0.025729 | 0.006655 | 0.029585 |
| 2.08 | 0.058289 | 0.8764 | 0.065311 | 0 | 0.202343 |
| 2.07 | 0.101092 | 0.659995 | 0.223647 | 0.015266 | 0.004566 |
| 2.01 | 0.02416 | 0.973681 | 0.00216 | 0 | 0.016484 |
| 1.97 | 0.063452 | 0.859274 | 0.077275 | 0 | 0.167713 |
| 1.95 | 0.021546 | 0.785222 | 0.093462 | 0.09977 | 0.001934 |
| 1.95 | 0.050315 | 0.598024 | 0.351513 | 0.000147 | 0.038121 |
| 1.94 | 0.069142 | 0.772286 | 0.14675 | 0.011822 | 0.039618 |
| 1.92 | 0.030356 | 0.662937 | 0.306707 | 0 | 0.021427 |
| 1.91 | 0.008343 | 0.991657 | 0 | 0 | 0.039714 |
| 1.91 | 0.017507 | 0.974532 | 0.00609 | 0.001872 | 0.18464 |
| 1.88 | 0.021748 | 0.928259 | 0.049992 | 7.31E-07 | 0.043612 |
| 1.88 | 0.025046 | 0.688707 | 0.286247 | 0 | 0.026522 |
| 1.88 | 0.074518 | 0.828824 | 0.096658 | 0 | 0.049843 |
| 1.81 | 0.030852 | 0.937414 | 0.022909 | 0.008825 | 0.063511 |
| 1.74 | 0.041708 | 0.943745 | 0 | 0.014547 | 0.008971 |
| 1.72 | 0.058124 | 0.890746 | 0.007969 | 0.043162 | 0.066551 |
| 1.72 | 0.122952 | 0.730519 | 0.14653 | 0 | 0.089381 |
| 1.66 | 0.135385 | 0.837915 | 0.0267 | 0 | 0.059579 |
| 1.66 | 0.110983 | 0.870325 | 0.018692 | 0 | 0.141334 |
| 1.65 | 0.006401 | 0.762863 | 0.215353 | 0.015384 | 0.120429 |
| 1.64 | 0.032421 | 0.811665 | 0.155915 | 0 | 0.079009 |
| 1.62 | 0.008458 | 0.766388 | 0.13723 | 0.087924 | 0.073515 |
| 1.61 | 0.048814 | 0.912767 | 0.038419 | 0 | 0.001488 |
| 1.59 | 0.049856 | 0.810036 | 0.140108 | 0 | 0.019612 |
| 1.56 | 0.046376 | 0.702486 | 0.251138 | 0 | 0.003461 |
| 1.55 | 0.055552 | 0.69975 | 0.239609 | 0.005088 | 0.003034 |
| 1.53 | 0.04742 | 0.747043 | 0.205537 | 0 | 0.027979 |
| 1.49 | 0.026939 | 0.973061 | 0 | 0 | 0.021787 |
| 1.48 | 0.096072 | 0.863331 | 0.040596 | 0 | 0.08757 |
| 1.46 | 0.028501 | 0.93417 | 0.037329 | 0 | 7.53E-06 |
| 1.44 | 0.222442 | 0.74335 | 0.034208 | 0 | 0.006338 |
| 1.42 | 0.023232 | 0.748449 | 0.204923 | 0.023397 | 0.029774 |
| 1.41 | 0.033119 | 0.862447 | 0.104434 | 0 | 0.024761 |
| 1.38 | 0.012567 | 0.970659 | 0.016774 | 0 | 0.011678 |
| 1.35 | 0.027806 | 0.664197 | 0.305735 | 0.002262 | 0.027812 |
| 1.33 | 0.022977 | 0.95925 | 0.006649 | 0.011124 | 0.025333 |
| 1.32 | 0.017027 | 0.946215 | 0.036758 | 0 | 0.005504 |
| 1.32 | 0.039363 | 0.856503 | 0.095384 | 0.00875 | 0.023128 |
| 1.32 | 0.068853 | 0.901758 | 0.029389 | 0 | 0.153666 |
| 1.3 | 0.052255 | 0.898211 | 0.046067 | 0.003468 | 0.000502 |
| 1.28 | 0.050745 | 0.719471 | 0.229783 | 0 | 0.077093 |
| 1.27 | 0.012289 | 0.967888 | 0.019822 | 0 | 0.169411 |
| 1.25 | 0.014656 | 0.937846 | 0.041141 | 0.006357 | 0.017017 |
| 1.23 | 0.056263 | 0.943737 | 0 | 0 | 0.41748 |
| 1.22 | 0.092467 | 0.907533 | 0 | 0 | 0.110143 |
| 1.22 | 0.035536 | 0.632685 | 0.323556 | 0.008224 | 0.021802 |
| 1.21 | 0.026149 | 0.925556 | 0.048294 | 0 | 0.048499 |
| 1.18 | 0.015126 | 0.759174 | 0.204304 | 0.021397 | 0.097869 |
| 1.17 | 0.031482 | 0.918601 | 0 | 0.049917 | 0.016405 |
| 1.17 | 0.042971 | 0.949884 | 0.007145 | 0 | 0.291543 |
| 1.16 | 0.04551 | 0.731711 | 0.222779 | 0 | 0.007162 |
| 1.15 | 0.070053 | 0.928604 | 0.001343 | 0 | 0.237388 |
| 1.14 | 0.014212 | 0.940359 | 0.02911 | 0.01632 | 0.085242 |
| 1.13 | 0.027328 | 0.959282 | 0.005139 | 0.008252 | 0.023328 |
| 1.13 | 0.074414 | 0.869734 | 0.019153 | 0.036699 | 0.003266 |
| 1.12 | 0.027304 | 0.917848 | 0.04837 | 0.006477 | 0.349861 |
| 1.1 | 0.008526 | 0.940563 | 0.050911 | 0 | 0.124726 |
| 1.07 | 0.016397 | 0.951369 | 0.032233 | 0 | 0.022951 |
| 1.04 | 0.007404 | 0.887462 | 0.105134 | 0 | 0.179264 |
| 1.01 | 0.059338 | 0.879605 | 0.056493 | 0 | 0.222732 |
| 0.95 | 0.006056 | 0.778152 | 0.174763 | 0.041029 | 0.032651 |
| 0.91 | 0.036539 | 0.951535 | 0.011926 | 0 | 0.025928 |
| 0.9 | 0.002975 | 0.947564 | 0.037218 | 0.012244 | 0.071134 |
| 0.9 | 0.099347 | 0.900653 | 0 | 0 | 0.147427 |
| 0.89 | 0.028831 | 0.777557 | 0.193612 | 0 | 0.01115 |
| 0.89 | 0.023466 | 0.646274 | 0.253975 | 0.066486 | 0.024883 |
| 0.84 | 0.007534 | 0.991543 | 0 | 0.000922 | 0.020689 |
| 0.84 | 0.072101 | 0.86385 | 0.054567 | 0.009482 | 0.064817 |
| 0.83 | 0.059954 | 0.694479 | 0.245567 | 0 | 0.029688 |
| 0.83 | 0.050363 | 0.949637 | 0 | 0 | 0.000103 |
| 0.8 | 0.01216 | 0.758907 | 0.228932 | 0 | 0.023113 |
| 0.79 | 0.168176 | 0.81787 | 0.013954 | 0 | 0.049296 |
| 0.78 | 0.18621 | 0.781401 | 0.032389 | 0 | 0.147147 |
| 0.77 | 0.059471 | 0.75569 | 0.181554 | 0.003285 | 0.032832 |
| 0.76 | 0.059469 | 0.940531 | 0 | 0 | 0.036402 |
| 0.76 | 0.022281 | 0.817366 | 0.144456 | 0.015897 | 0.002968 |
| 0.75 | 0.021006 | 0.91466 | 0.064334 | 0 | 0.031401 |
| 0.73 | 0.040363 | 0.948775 | 0.010863 | 0 | 0.06336 |
| 0.72 | 0.009221 | 0.887556 | 0.073236 | 0.029987 | 0.245076 |
| 0.71 | 0.004923 | 0.757164 | 0.144813 | 0.0931 | 0.011441 |
| 0.67 | 0.052039 | 0.626445 | 0.321516 | 0 | 0.038994 |
| 0.67 | 0.035435 | 0.823689 | 0.105185 | 0.035691 | 0.047584 |
| 0.66 | 0.022698 | 0.676984 | 0.300318 | 0 | 0.086161 |
| 0.64 | 0.050098 | 0.942054 | 0.007554 | 0.000294 | 0.032512 |
| 0.64 | 0.049996 | 0.803418 | 0.146586 | 0 | 0.137218 |
| 0.58 | 0.018223 | 0.50482 | 0.425159 | 0 | 0.016896 |
| 0.55 | 0.011426 | 0.976589 | 0.011985 | 0 | 0.007056 |
| 0.52 | 0.216664 | 0.779225 | 0.004111 | 0 | 0.294511 |
| 0.48 | 0.153329 | 0.620561 | 0.18755 | 0.031629 | 0.091145 |
| 0.45 | 0.056379 | 0.943621 | 0 | 0 | 0.123161 |
| 0.43 | 0.037419 | 0.923312 | 0.010583 | 0.028685 | 0.061092 |
| 0.41 | 0.056828 | 0.90978 | 0.014798 | 0.018594 | 0.01458 |
| 0.41 | 0.020885 | 0.97362 | 0.005495 | 0 | 0.031392 |
| 0.39 | 0.031151 | 0.746825 | 0.203347 | 0.018677 | 0.101497 |
| 0.37 | 0.097723 | 0.899411 | 0.002866 | 0 | 0.018141 |
| 0.33 | 0.03398 | 0.965065 | 0.000956 | 0 | 0.457655 |
| 0.33 | 0.010983 | 0.903515 | 0.085502 | 0 | 0.036375 |
| 0.24 | 0.11287 | 0.864337 | 0.022793 | 0 | 0.094936 |
| 0.24 | 0.03865 | 0.910378 | 0.050972 | 0 | 0.003528 |
| 0.2 | 0.04777 | 0.769868 | 0.182362 | 0 | 0.025439 |
| 0.2 | 0.039863 | 0.711922 | 0.248216 | 0 | 0.020078 |
| 0.19 | 0.054189 | 0.877665 | 0.068146 | 0 | 0.141787 |
| 0.13 | 0.026963 | 0.570467 | 0.183307 | 0.219263 | 0.0174 |

*Red fox*

| Harvest  (ind./100ha) | SETTLEMENTS  (%) | CROPS  (%) | FORESTS  (%) | WATER  (%) | WIND FARM  (%) |
| --- | --- | --- | --- | --- | --- |
| 60.26 | 0.130492 | 0.869508 | 0 | 0 | 0.036249 |
| 52.77 | 0.034493 | 0.812457 | 0.153051 | 0 | 0.073334 |
| 38.28 | 0.096072 | 0.863331 | 0.040596 | 0 | 0.08757 |
| 33.8 | 0.027328 | 0.959282 | 0.005139 | 0.008252 | 0.023328 |
| 31.31 | 0.18621 | 0.781401 | 0.032389 | 0 | 0.147147 |
| 29.8 | 0.086482 | 0.913518 | 0 | 0 | 0.005667 |
| 29.78 | 0.037227 | 0.774049 | 0.188724 | 0 | 0.136711 |
| 27.4 | 0.025046 | 0.688707 | 0.286247 | 0 | 0.026522 |
| 27.14 | 0.007743 | 0.882418 | 0.104562 | 0.005277 | 0.030295 |
| 26.94 | 0.039445 | 0.880548 | 0.078509 | 0.001499 | 0.00146 |
| 26.75 | 0.012081 | 0.564216 | 0.390193 | 0.03351 | 0.035279 |
| 26.33 | 0.017027 | 0.946215 | 0.036758 | 0 | 0.005504 |
| 25.9 | 0.069142 | 0.772286 | 0.14675 | 0.011822 | 0.039618 |
| 25.21 | 0.055185 | 0.873829 | 0.070986 | 0 | 0.060068 |
| 25.08 | 0.070047 | 0.841396 | 0.083412 | 0.005146 | 0.038956 |
| 24.38 | 0.003095 | 0.950925 | 0.015182 | 0.030799 | 0.103433 |
| 23.96 | 0.078715 | 0.778306 | 0.133458 | 0.009521 | 0.022315 |
| 23.82 | 0.050363 | 0.949637 | 0 | 0 | 0.000103 |
| 23.52 | 0.043407 | 0.941455 | 0.015138 | 0 | 0.002294 |
| 23.21 | 0.032386 | 0.701748 | 0.255853 | 0.010013 | 0.027642 |
| 21.99 | 0.034684 | 0.90422 | 0.061095 | 0 | 0.079926 |
| 21.63 | 0.049629 | 0.944396 | 0.005975 | 0 | 0.171729 |
| 21.48 | 0.069289 | 0.841813 | 0.088899 | 0 | 0.074837 |
| 21.47 | 0.065776 | 0.916671 | 0.017553 | 0 | 0.021232 |
| 21.44 | 0.058012 | 0.561444 | 0.380544 | 0 | 0.028502 |
| 21.36 | 0.045885 | 0.95365 | 0.000465 | 0 | 0.003323 |
| 21.03 | 0.06371 | 0.687375 | 0.248915 | 0 | 0.024296 |
| 20.92 | 0.033275 | 0.900866 | 0.060343 | 0.005515 | 0.091831 |
| 20.89 | 0.03309 | 0.827144 | 0.139765 | 0 | 0.030663 |
| 20.78 | 0.070103 | 0.80001 | 0.098141 | 0.031745 | 0.019618 |
| 20.55 | 0.039363 | 0.856503 | 0.095384 | 0.00875 | 0.023128 |
| 20.46 | 0.046888 | 0.885726 | 0.036393 | 0.030993 | 0.012365 |
| 20.35 | 0.092472 | 0.841024 | 0.066505 | 0 | 0.094696 |
| 20.3 | 0.035435 | 0.823689 | 0.105185 | 0.035691 | 0.047584 |
| 20.13 | 0.029025 | 0.753063 | 0.182242 | 0.03567 | 0.000693 |
| 20 | 0.102583 | 0.897417 | 0 | 0 | 0.045341 |
| 19.9 | 0.025498 | 0.828399 | 0.114062 | 0.032041 | 0.078826 |
| 19.86 | 0.134972 | 0.724281 | 0.120111 | 0.020636 | 0.008062 |
| 19.64 | 0.050548 | 0.895549 | 0.053477 | 0.000426 | 0.040513 |
| 19.64 | 0.01756 | 0.936756 | 0.045684 | 0 | 0.587545 |
| 19.39 | 0.029447 | 0.903798 | 0.066755 | 0 | 0.23376 |
| 19.2 | 0.037255 | 0.909058 | 0.053687 | 0 | 0.110157 |
| 19.13 | 0.011426 | 0.976589 | 0.011985 | 0 | 0.007056 |
| 19.1 | 0.074807 | 0.798245 | 0.126948 | 0 | 0.052297 |
| 19.06 | 0.059469 | 0.940531 | 0 | 0 | 0.036402 |
| 19.05 | 0.052039 | 0.626445 | 0.321516 | 0 | 0.038994 |
| 19.03 | 0.019528 | 0.767714 | 0.212758 | 0 | 0.030766 |
| 18.89 | 0.046884 | 0.923363 | 0.029753 | 0 | 0.00521 |
| 18.42 | 0.052708 | 0.717515 | 0.2169 | 0.012876 | 0.308219 |
| 18.34 | 0.054568 | 0.700053 | 0.236636 | 0.008742 | 0.051978 |
| 17.93 | 0.017507 | 0.974532 | 0.00609 | 0.001872 | 0.18464 |
| 17.91 | 0.060047 | 0.690705 | 0.249248 | 0 | 0.327264 |
| 17.86 | 0.040323 | 0.76684 | 0.192836 | 0 | 0.016819 |
| 17.84 | 0.137039 | 0.844241 | 0.01872 | 0 | 0.054031 |
| 17.82 | 0.036539 | 0.951535 | 0.011926 | 0 | 0.025928 |
| 17.48 | 0.030872 | 0.686417 | 0.272097 | 0.010614 | 0.010509 |
| 17.47 | 0.203607 | 0.526209 | 0.270184 | 0 | 0.050672 |
| 17.44 | 0.114896 | 0.770582 | 0.111487 | 0.003035 | 0.057439 |
| 17.43 | 0.062328 | 0.768301 | 0.146098 | 0.023273 | 0.041255 |
| 17.24 | 0.061571 | 0.728748 | 0.209681 | 0 | 0.041562 |
| 17.21 | 0.065527 | 0.728519 | 0.126969 | 0.078986 | 0.047136 |
| 17.14 | 0.043339 | 0.663185 | 0.278789 | 0.014687 | 0.102922 |
| 17.13 | 0.21499 | 0.702711 | 0.080053 | 0.002246 | 0.007246 |
| 16.91 | 0.01082 | 0.979523 | 0 | 0.009656 | 0.210532 |
| 16.84 | 0.033119 | 0.862447 | 0.104434 | 0 | 0.024761 |
| 16.81 | 0.077374 | 0.85315 | 0.00706 | 0.062416 | 0.027737 |
| 16.77 | 0.05913 | 0.575212 | 0.365601 | 5.72E-05 | 0.03847 |
| 16.76 | 0.012289 | 0.967888 | 0.019822 | 0 | 0.169411 |
| 16.71 | 0.028501 | 0.93417 | 0.037329 | 0 | 7.53E-06 |
| 16.67 | 0.015311 | 0.850012 | 0.134677 | 0 | 0.043252 |
| 16.57 | 0.03398 | 0.965065 | 0.000956 | 0 | 0.457655 |
| 16.49 | 0.039215 | 0.907213 | 0.053573 | 0 | 0.070329 |
| 16.44 | 0.021317 | 0.804667 | 0.174016 | 0 | 0.278978 |
| 16.39 | 0.060716 | 0.682299 | 0.256985 | 0 | 0.053094 |
| 16.28 | 0.028579 | 0.779842 | 0.184295 | 0.007283 | 0.00428 |
| 16.27 | 0.041239 | 0.678391 | 0.280371 | 0 | 0.166558 |
| 16.24 | 0.066093 | 0.856123 | 0.077784 | 0 | 0.032206 |
| 16.22 | 0.222442 | 0.74335 | 0.034208 | 0 | 0.006338 |
| 16.11 | 0.028434 | 0.931806 | 0.027306 | 0.012453 | 0.127255 |
| 16.05 | 0.11188 | 0.782154 | 0.105966 | 0 | 0.001959 |
| 15.92 | 0.045044 | 0.886899 | 0.026934 | 0.041122 | 0.00106 |
| 15.85 | 0.110983 | 0.870325 | 0.018692 | 0 | 0.141334 |
| 15.67 | 0.046194 | 0.608006 | 0.345294 | 0.000506 | 0.002681 |
| 15.64 | 0.040363 | 0.948775 | 0.010863 | 0 | 0.06336 |
| 15.57 | 0.022698 | 0.676984 | 0.300318 | 0 | 0.086161 |
| 15.51 | 0.048955 | 0.647511 | 0.303534 | 0 | 0.045488 |
| 15.5 | 0.168176 | 0.81787 | 0.013954 | 0 | 0.049296 |
| 15.44 | 0.072839 | 0.741172 | 0.185989 | 0 | 0.02913 |
| 15.31 | 0.001377 | 0.682013 | 0.307943 | 0.008666 | 0.024811 |
| 15.29 | 0.03957 | 0.723527 | 0.232789 | 0.004115 | 0.000244 |
| 15.26 | 0.025069 | 0.954991 | 0.01994 | 0 | 0.029211 |
| 15.21 | 0.051852 | 0.630668 | 0.304929 | 0.01255 | 0.049537 |
| 15.14 | 0.009555 | 0.979002 | 0.000198 | 0.011245 | 0.076538 |
| 15 | 0.05676 | 0.713201 | 0.23004 | 0 | 0.025905 |
| 15 | 0.055696 | 0.879582 | 0.060887 | 0.003835 | 0.021538 |
| 14.93 | 0.039739 | 0.691329 | 0.268932 | 0 | 0.195716 |
| 14.84 | 0.026939 | 0.973061 | 0 | 0 | 0.021787 |
| 14.77 | 0.053764 | 0.773062 | 0.173173 | 0 | 0.151376 |
| 14.71 | 0.110639 | 0.740989 | 0.108626 | 0.039746 | 0.079146 |
| 14.64 | 0.108231 | 0.891769 | 0 | 0 | 0.081012 |
| 14.61 | 0.032947 | 0.773658 | 0.145192 | 0.048202 | 0.049896 |
| 14.61 | 0.077451 | 0.901627 | 0.020923 | 0 | 0.037246 |
| 14.55 | 0.073033 | 0.813903 | 0.097747 | 0.015317 | 0.19531 |
| 14.54 | 0.139056 | 0.729618 | 0.11691 | 0.014416 | 2.54E-05 |
| 14.53 | 0.181455 | 0.617007 | 0.187585 | 0.013953 | 0.12495 |
| 14.49 | 0.045738 | 0.828349 | 0.125913 | 0 | 0.022493 |
| 14.43 | 0.035595 | 0.757742 | 0.206663 | 0 | 0.026593 |
| 14.4 | 0.030193 | 0.919865 | 0.019158 | 0.030784 | 0.093644 |
| 14.34 | 0.02551 | 0.914172 | 0.045998 | 0.01432 | 0.02754 |
| 14.32 | 0.108022 | 0.691244 | 0.180332 | 0.020402 | 0.004315 |
| 14.28 | 0.054088 | 0.749397 | 0.163075 | 0.03344 | 0.116868 |
| 14.25 | 0.130716 | 0.674756 | 0.194527 | 0 | 0.138547 |
| 14.16 | 0.141596 | 0.638012 | 0.156134 | 0.064258 | 0.108228 |
| 14.14 | 0.02289 | 0.955775 | 0.021335 | 0 | 0.120855 |
| 14.13 | 0.043831 | 0.54447 | 0.298957 | 0.112741 | 0.002629 |
| 14.12 | 0.052284 | 0.743996 | 0.193735 | 0.009985 | 0.045387 |
| 14.08 | 0.074589 | 0.640797 | 0.26404 | 0.020574 | 0.021459 |
| 14.07 | 0.025413 | 0.787728 | 0.186858 | 0 | 0.021162 |
| 14.04 | 0.064454 | 0.807537 | 0.118761 | 0.009248 | 0.039809 |
| 13.98 | 0.095858 | 0.675914 | 0.223198 | 0.005031 | 0.017825 |
| 13.73 | 0.027806 | 0.664197 | 0.305735 | 0.002262 | 0.027812 |
| 13.72 | 0.043952 | 0.822823 | 0.084006 | 0.049219 | 0.044707 |
| 13.59 | 0.108214 | 0.736279 | 0.126343 | 0.029164 | 0.000839 |
| 13.59 | 0.025165 | 0.709589 | 0.265246 | 0 | 0.340679 |
| 13.57 | 0.03637 | 0.8723 | 0.091329 | 0 | 0.000179 |
| 13.55 | 0.059338 | 0.879605 | 0.056493 | 0 | 0.222732 |
| 13.54 | 0.032369 | 0.54685 | 0.415187 | 0.005593 | 0.023739 |
| 13.51 | 0.036312 | 0.904341 | 0.024842 | 0.034504 | 0.160208 |
| 13.44 | 0.142331 | 0.782279 | 0.058797 | 0.016592 | 0.041235 |
| 13.41 | 0.122952 | 0.730519 | 0.14653 | 0 | 0.089381 |
| 13.34 | 0.029263 | 0.64246 | 0.263832 | 0.064444 | 0.068015 |
| 13.33 | 0.022977 | 0.95925 | 0.006649 | 0.011124 | 0.025333 |
| 13.27 | 0.012828 | 0.801466 | 0.169907 | 0.015799 | 0.035618 |
| 13.22 | 0.080689 | 0.873851 | 0.041035 | 0.004425 | 0.180591 |
| 13.14 | 0.004494 | 0.931113 | 0.025152 | 0.03924 | 0.009948 |
| 13.1 | 0.019231 | 0.918595 | 0.062174 | 0 | 0.02441 |
| 13.09 | 0.023232 | 0.748449 | 0.204923 | 0.023397 | 0.029774 |
| 13.08 | 0.02225 | 0.900537 | 0.067932 | 0.00928 | 0.000577 |
| 12.9 | 0.056379 | 0.943621 | 0 | 0 | 0.123161 |
| 12.84 | 0.009447 | 0.761519 | 0.21221 | 0.016824 | 0.01016 |
| 12.78 | 0.012366 | 0.881566 | 0.053689 | 0.052379 | 0.104641 |
| 12.78 | 0.049055 | 0.900592 | 0.050353 | 0 | 0.099 |
| 12.76 | 0.033544 | 0.839022 | 0.127434 | 0 | 0.062529 |
| 12.75 | 0.049931 | 0.628573 | 0.321228 | 0.000269 | 0.036331 |
| 12.66 | 0.027717 | 0.596513 | 0.370443 | 0.005327 | 0.035165 |
| 12.63 | 0.017478 | 0.896971 | 0.020908 | 0.064643 | 0.124726 |
| 12.6 | 0.096224 | 0.839476 | 0.0643 | 0 | 0.055361 |
| 12.59 | 0.182413 | 0.806462 | 0.011125 | 0 | 0.037714 |
| 12.57 | 0.048432 | 0.813801 | 0.137767 | 0 | 0.016043 |
| 12.52 | 0.051785 | 0.901516 | 0.046699 | 0 | 0.007171 |
| 12.46 | 0.024205 | 0.53313 | 0.202177 | 0.240487 | 0.002058 |
| 12.41 | 0.026258 | 0.530627 | 0.436197 | 0.006918 | 0.028396 |
| 12.29 | 0.010139 | 0.929534 | 0.039062 | 0.021265 | 0.02452 |
| 12.29 | 0.005309 | 0.943367 | 0.02594 | 0.025384 | 0.000813 |
| 12.2 | 0.01216 | 0.758907 | 0.228932 | 0 | 0.023113 |
| 12.18 | 0.042169 | 0.597119 | 0.360712 | 0 | 0.036652 |
| 12.15 | 0.080504 | 0.831155 | 0.005685 | 0.082657 | 0.041065 |
| 12.15 | 0.058187 | 0.528333 | 0.413479 | 0 | 0.002676 |
| 12.08 | 0.048814 | 0.912767 | 0.038419 | 0 | 0.001488 |
| 12.06 | 0.009221 | 0.887556 | 0.073236 | 0.029987 | 0.245076 |
| 12.05 | 0.040076 | 0.833117 | 0.087333 | 0.039474 | 0.241711 |
| 12.03 | 0.058021 | 0.662699 | 0.258411 | 0.020869 | 0.038288 |
| 11.99 | 0.008526 | 0.940563 | 0.050911 | 0 | 0.124726 |
| 11.86 | 0.077906 | 0.776797 | 0.145296 | 0 | 0.026626 |
| 11.84 | 0.063263 | 0.772515 | 0.155506 | 0.008716 | 0.17232 |
| 11.81 | 0.021546 | 0.785222 | 0.093462 | 0.09977 | 0.001934 |
| 11.74 | 0.029683 | 0.738291 | 0.186006 | 0.046019 | 0.017025 |
| 11.72 | 0.105751 | 0.72069 | 0.166077 | 0.007481 | 0.034231 |
| 11.71 | 0.050611 | 0.799196 | 0.130295 | 0.019897 | 0.045145 |
| 11.64 | 0.036485 | 0.81515 | 0.148365 | 0 | 0.19155 |
| 11.62 | 0.023363 | 0.846999 | 0.129638 | 0 | 0.078641 |
| 11.51 | 0.097723 | 0.899411 | 0.002866 | 0 | 0.018141 |
| 11.48 | 0.08025 | 0.857246 | 0.062497 | 7.34E-06 | 0.108955 |
| 11.47 | 0.01712 | 0.800569 | 0.132823 | 0.049489 | 0.06699 |
| 11.41 | 0.032367 | 0.938775 | 0.019579 | 0.009278 | 0.008665 |
| 11.38 | 0.111642 | 0.775675 | 0.112682 | 0 | 0.047805 |
| 11.38 | 0.072977 | 0.856731 | 0.070292 | 0 | 0.074098 |
| 11.29 | 0.055552 | 0.69975 | 0.239609 | 0.005088 | 0.003034 |
| 11.25 | 0.028499 | 0.562559 | 0.360137 | 0.048805 | 0.001166 |
| 11.24 | 0.034773 | 0.709889 | 0.247028 | 0.008309 | 0.059819 |
| 11.2 | 0.041708 | 0.943745 | 0 | 0.014547 | 0.008971 |
| 11.15 | 0 | 0.891838 | 0.108162 | 0 | 0.002346 |
| 11.03 | 0.060899 | 0.775398 | 0.163702 | 0 | 0.022238 |
| 11 | 0.04838 | 0.768229 | 0.137646 | 0.045745 | 0.000874 |
| 10.98 | 0.157547 | 0.729757 | 0.10907 | 0.003625 | 0.027969 |
| 10.97 | 0.021748 | 0.928259 | 0.049992 | 7.31E-07 | 0.043612 |
| 10.96 | 0.068853 | 0.901758 | 0.029389 | 0 | 0.153666 |
| 10.92 | 0.046235 | 0.768727 | 0.185038 | 0 | 0.003722 |
| 10.85 | 0.030864 | 0.930063 | 0 | 0.039073 | 0.003036 |
| 10.83 | 0.010053 | 0.727504 | 0.207121 | 0.055322 | 0.048494 |
| 10.73 | 0.043956 | 0.854424 | 0.090371 | 0.011248 | 0.042874 |
| 10.67 | 0.028999 | 0.817861 | 0.15314 | 0 | 0.078066 |
| 10.63 | 0.102823 | 0.881072 | 0.016105 | 0 | 0.048898 |
| 10.62 | 0.014883 | 0.952852 | 0.026053 | 0.006211 | 0.039717 |
| 10.54 | 0.005345 | 0.589222 | 0.392616 | 0.012817 | 0.022783 |
| 10.48 | 0.025748 | 0.720481 | 0.253771 | 0 | 0.036214 |
| 10.44 | 0.071363 | 0.916463 | 0.012174 | 0 | 0.181614 |
| 10.43 | 0.010679 | 0.952 | 0.001661 | 0.035661 | 0.004639 |
| 10.42 | 0.059391 | 0.887374 | 0.052975 | 0.00026 | 0.002722 |
| 10.38 | 0.038604 | 0.954512 | 0.006884 | 0 | 0.02608 |
| 10.35 | 0.036625 | 0.770464 | 0.167258 | 0.025652 | 0.178928 |
| 10.34 | 0.08957 | 0.6941 | 0.21633 | 0 | 0.055495 |
| 10.34 | 0.021547 | 0.897054 | 0.081399 | 0 | 0.003304 |
| 10.21 | 0.044407 | 0.778417 | 0.176709 | 0.000467 | 0.032712 |
| 10.2 | 0.036346 | 0.809759 | 0.128229 | 0.025666 | 0.037496 |
| 10.2 | 0.039022 | 0.789893 | 0.171085 | 0 | 0.011916 |
| 10.19 | 0.021306 | 0.96547 | 0.000434 | 0.01279 | 0.093825 |
| 10.18 | 0.059243 | 0.805019 | 0.135738 | 0 | 0.094313 |
| 10.17 | 0.032721 | 0.728697 | 0.238522 | 5.97E-05 | 0.018052 |
| 10.12 | 0.029013 | 0.883584 | 0.082962 | 0.004441 | 0.000183 |
| 10.05 | 0.072737 | 0.703643 | 0.172948 | 0.050672 | 0.003121 |
| 10.03 | 0.050745 | 0.719471 | 0.229783 | 0 | 0.077093 |
| 10.03 | 0.085555 | 0.904837 | 0.009608 | 0 | 0.034194 |
| 9.97 | 0.063612 | 0.891342 | 0.045046 | 0 | 0.107764 |
| 9.95 | 0.076638 | 0.893076 | 0.030286 | 0 | 0.003956 |
| 9.88 | 0.023832 | 0.952769 | 0.012307 | 0.011092 | 0.083228 |
| 9.72 | 0.011822 | 0.922221 | 0.0577 | 0.008256 | 0.027789 |
| 9.7 | 0.020053 | 0.595764 | 0.384183 | 0 | 0.022035 |
| 9.64 | 0.04551 | 0.731711 | 0.222779 | 0 | 0.007162 |
| 9.62 | 0.119335 | 0.541145 | 0.336507 | 0.003013 | 0.018434 |
| 9.61 | 0.056828 | 0.90978 | 0.014798 | 0.018594 | 0.01458 |
| 9.54 | 0.041801 | 0.944368 | 0 | 0.013831 | 0.010372 |
| 9.53 | 0.020504 | 0.893617 | 0.085879 | 0 | 0.028256 |
| 9.53 | 0.018482 | 0.571366 | 0.403494 | 0.006658 | 0.08103 |
| 9.45 | 0.035856 | 0.89092 | 0.073224 | 0 | 0.152419 |
| 9.35 | 0.036829 | 0.673528 | 0.283352 | 0.006291 | 0.031069 |
| 9.32 | 0.137764 | 0.692755 | 0.169481 | 0 | 0.01181 |
| 9.32 | 0.064382 | 0.647883 | 0.274131 | 0.013604 | 0.054466 |
| 9.32 | 0.031482 | 0.918601 | 0 | 0.049917 | 0.016405 |
| 9.29 | 0.134499 | 0.702936 | 0.162566 | 0 | 0.002641 |
| 9.26 | 0.054189 | 0.877665 | 0.068146 | 0 | 0.141787 |
| 9.25 | 0.03372 | 0.954883 | 0.011397 | 0 | 0.059214 |
| 9.23 | 0.069296 | 0.774471 | 0.070706 | 0.085528 | 0.031938 |
| 9.22 | 0.04601 | 0.785236 | 0.14245 | 0.026304 | 0.131842 |
| 9.19 | 0.023883 | 0.761086 | 0.215031 | 0 | 0.033082 |
| 9.15 | 0.037281 | 0.903436 | 0.042729 | 0.016554 | 0.047312 |
| 9.15 | 0.095985 | 0.700639 | 0.198975 | 0.0044 | 0.02055 |
| 9.13 | 0.068708 | 0.849552 | 0.042768 | 0.038973 | 0.074069 |
| 9.13 | 0.012225 | 0.952622 | 0.035153 | 0 | 0.01266 |
| 9.12 | 0.041745 | 0.712863 | 0.243395 | 0.001997 | 0.02306 |
| 9.1 | 0.028556 | 0.832744 | 0.1387 | 0 | 0.027699 |
| 9.08 | 0.075708 | 0.831813 | 0.092479 | 0 | 0.125231 |
| 9.05 | 0.027274 | 0.638686 | 0.329299 | 0.004741 | 0.087119 |
| 9.03 | 0.035474 | 0.794716 | 0.16981 | 0 | 0.222865 |
| 8.95 | 0.036323 | 0.95832 | 0.005356 | 0 | 0.348035 |
| 8.89 | 0.12475 | 0.506296 | 0.368954 | 0 | 0.018843 |
| 8.88 | 0.004923 | 0.757164 | 0.144813 | 0.0931 | 0.011441 |
| 8.83 | 0.053152 | 0.914463 | 0.025729 | 0.006655 | 0.029585 |
| 8.83 | 0.098187 | 0.786894 | 0.114919 | 0 | 0.033695 |
| 8.78 | 0.020569 | 0.771368 | 0.155748 | 0.052315 | 0.015713 |
| 8.78 | 0.059954 | 0.694479 | 0.245567 | 0 | 0.029688 |
| 8.76 | 0.06005 | 0.93995 | 0 | 0 | 0.023202 |
| 8.75 | 0.018303 | 0.841083 | 0.134267 | 0.006347 | 0.007471 |
| 8.74 | 0.067705 | 0.60951 | 0.322784 | 0 | 0.031049 |
| 8.72 | 0.041876 | 0.634991 | 0.323132 | 0 | 0.019803 |
| 8.68 | 0.059836 | 0.773228 | 0.059752 | 0.107184 | 0.135669 |
| 8.67 | 0.051831 | 0.828658 | 0.11951 | 0 | 0.107904 |
| 8.63 | 0.007404 | 0.887462 | 0.105134 | 0 | 0.179264 |
| 8.59 | 0.04377 | 0.927657 | 0.028573 | 0 | 0.115429 |
| 8.55 | 0.039287 | 0.659343 | 0.239964 | 0.061407 | 0.021402 |
| 8.41 | 0.086936 | 0.888209 | 0.024855 | 0 | 0.080122 |
| 8.34 | 0.046998 | 0.826689 | 0.126312 | 0 | 0.087959 |
| 8.32 | 0.153329 | 0.620561 | 0.18755 | 0.031629 | 0.091145 |
| 8.28 | 0.012878 | 0.947298 | 0.03675 | 0.003074 | 0.028568 |
| 8.25 | 0.032421 | 0.811665 | 0.155915 | 0 | 0.079009 |
| 8.25 | 0.074518 | 0.828824 | 0.096658 | 0 | 0.049843 |
| 8.21 | 0.030907 | 0.705282 | 0.26381 | 0 | 0.210419 |
| 8.2 | 0.014212 | 0.940359 | 0.02911 | 0.01632 | 0.085242 |
| 8.16 | 0.07946 | 0.663096 | 0.257444 | 0 | 0.119837 |
| 8.16 | 0.012941 | 0.611311 | 0.36866 | 0.007087 | 0.050402 |
| 8.15 | 0.053113 | 0.649051 | 0.261498 | 0.036338 | 0.042373 |
| 8.14 | 0.045934 | 0.924793 | 0.02209 | 0.007184 | 0.19778 |
| 8.13 | 0.048241 | 0.890315 | 0.061444 | 0 | 0.004877 |
| 8.13 | 0.033665 | 0.827557 | 0.095811 | 0.042966 | 0.085835 |
| 8.1 | 0.020885 | 0.97362 | 0.005495 | 0 | 0.031392 |
| 8.08 | 0.041354 | 0.793022 | 0.109663 | 0.055961 | 0.156689 |
| 8.05 | 0.022756 | 0.827933 | 0.149312 | 0 | 0.036017 |
| 8.04 | 0.053881 | 0.915746 | 0.006846 | 0.023526 | 0.034166 |
| 8.01 | 0.059068 | 0.905269 | 0.035663 | 0 | 0.061176 |
| 8 | 0.04376 | 0.936753 | 0.019487 | 0 | 0.061764 |
| 7.96 | 0.029145 | 0.731754 | 0.289111 | 0 | 0.037475 |
| 7.95 | 0.018063 | 0.899386 | 0.044394 | 0.038157 | 0.085235 |
| 7.93 | 0.008032 | 0.962155 | 0.004333 | 0.02548 | 0.026076 |
| 7.92 | 0.039841 | 0.816561 | 0.143598 | 0 | 0.101836 |
| 7.9 | 0.015465 | 0.974209 | 0.010326 | 0 | 0.001597 |
| 7.89 | 0.065419 | 0.70728 | 0.227301 | 0 | 0.288139 |
| 7.88 | 0.014978 | 0.876519 | 0.099945 | 0.008559 | 0.01608 |
| 7.87 | 0.01543 | 0.653371 | 0.299888 | 0.031312 | 0.052174 |
| 7.87 | 0.072476 | 0.909047 | 0.018477 | 0 | 0.060021 |
| 7.86 | 0.031111 | 0.552667 | 0.394879 | 0.021342 | 0.026238 |
| 7.8 | 0.059471 | 0.75569 | 0.181554 | 0.003285 | 0.032832 |
| 7.8 | 0.016053 | 0.877654 | 0.080995 | 0.025299 | 0.022378 |
| 7.78 | 0.042068 | 0.913719 | 0.035636 | 0.008577 | 0.070134 |
| 7.75 | 0.046989 | 0.861338 | 0.091672 | 0 | 0.288311 |
| 7.72 | 0.100035 | 0.878576 | 0 | 0.021389 | 0.004507 |
| 7.71 | 0.04306 | 0.876667 | 0.080273 | 0 | 0.034083 |
| 7.69 | 0.124811 | 0.814342 | 0.060847 | 0 | 0.105066 |
| 7.65 | 0.016397 | 0.951369 | 0.032233 | 0 | 0.022951 |
| 7.64 | 0.015126 | 0.759174 | 0.204304 | 0.021397 | 0.097869 |
| 7.59 | 0.021237 | 0.959433 | 0.019331 | 0 | 0.256702 |
| 7.57 | 0.011447 | 0.645638 | 0.323308 | 0.019607 | 0.001809 |
| 7.57 | 0.034088 | 0.734203 | 0.231709 | 0 | 0.003313 |
| 7.57 | 0.040823 | 0.959177 | 0 | 0 | 0.004214 |
| 7.5 | 0.013851 | 0.854947 | 0.094772 | 0.036431 | 0.005141 |
| 7.49 | 0.033849 | 0.683169 | 0.28066 | 0.002321 | 0.049347 |
| 7.46 | 0.07399 | 0.807539 | 0.022472 | 0.096 | 0.028334 |
| 7.46 | 0.066768 | 0.576755 | 0.179528 | 0.176949 | 0.046495 |
| 7.37 | 0.092208 | 0.70089 | 0.190913 | 0.015988 | 0.053543 |
| 7.33 | 0.135385 | 0.837915 | 0.0267 | 0 | 0.059579 |
| 7.32 | 0.02038 | 0.918323 | 0.036001 | 0.025295 | 0.032543 |
| 7.32 | 0.142246 | 0.407022 | 0.377298 | 0.073434 | 0.089493 |
| 7.31 | 0.036113 | 0.84386 | 0.028627 | 0.0914 | 0.01802 |
| 7.29 | 0.01512 | 0.858559 | 0.07309 | 0.053231 | 0.037677 |
| 7.27 | 0.050315 | 0.598024 | 0.351513 | 0.000147 | 0.038121 |
| 7.16 | 0.02416 | 0.973681 | 0.00216 | 0 | 0.016484 |
| 7.16 | 0.092467 | 0.907533 | 0 | 0 | 0.110143 |
| 7.16 | 0.216664 | 0.779225 | 0.004111 | 0 | 0.294511 |
| 7.14 | 0.058124 | 0.890746 | 0.007969 | 0.043162 | 0.066551 |
| 7.11 | 0.112847 | 0.540884 | 0.2825 | 0.063769 | 0.234756 |
| 7.05 | 0.0599 | 0.70447 | 0.203372 | 0.032257 | 0.035467 |
| 7.04 | 0.101092 | 0.659995 | 0.223647 | 0.015266 | 0.004566 |
| 6.98 | 0.007534 | 0.991543 | 0 | 0.000922 | 0.020689 |
| 6.96 | 0.031613 | 0.961818 | 0.003158 | 0.003411 | 0.046069 |
| 6.9 | 0.041867 | 0.696324 | 0.261809 | 0 | 0.009857 |
| 6.88 | 0.026162 | 0.710156 | 0.240745 | 0.022937 | 0.031545 |
| 6.8 | 0.034103 | 0.818442 | 0.147454 | 0 | 0.087954 |
| 6.75 | 0.003406 | 0.599878 | 0.396716 | 0 | 0.045399 |
| 6.73 | 0.027304 | 0.917848 | 0.04837 | 0.006477 | 0.349861 |
| 6.72 | 0.017755 | 0.694353 | 0.28756 | 0.000331 | 0.007311 |
| 6.71 | 0.065773 | 0.829725 | 0.103836 | 0.000666 | 0.181817 |
| 6.69 | 0.028831 | 0.777557 | 0.193612 | 0 | 0.01115 |
| 6.69 | 0.016972 | 0.707628 | 0.225538 | 0.049862 | 0.111867 |
| 6.62 | 0.04557 | 0.58709 | 0.36734 | 0 | 0.070527 |
| 6.61 | 0.11287 | 0.864337 | 0.022793 | 0 | 0.094936 |
| 6.57 | 0.030385 | 0.902018 | 0.026566 | 0.041031 | 0.017606 |
| 6.53 | 0.039787 | 0.928108 | 0.016795 | 0.015311 | 0.04215 |
| 6.51 | 0.046144 | 0.680903 | 0.254461 | 0.018493 | 0.000879 |
| 6.5 | 0.029175 | 0.877794 | 0.093031 | 0 | 0.015471 |
| 6.48 | 0.143477 | 0.843699 | 0.006261 | 0.006563 | 0.063003 |
| 6.47 | 0.02159 | 0.743128 | 0.235281 | 0 | 0.058284 |
| 6.43 | 0.027087 | 0.892398 | 0.06958 | 0.010935 | 0.015594 |
| 6.4 | 0.151948 | 0.530499 | 0.120228 | 0.197325 | 0.019356 |
| 6.38 | 0.042539 | 0.616348 | 0.341113 | 0 | 0.050441 |
| 6.36 | 0.006401 | 0.762863 | 0.215353 | 0.015384 | 0.120429 |
| 6.34 | 0.012567 | 0.970659 | 0.016774 | 0 | 0.011678 |
| 6.27 | 0.025726 | 0.568486 | 0.358865 | 0.046922 | 0.138587 |
| 6.27 | 0.032981 | 0.830037 | 0.110184 | 0.026798 | 0.145802 |
| 6.25 | 0.077519 | 0.685448 | 0.237032 | 0 | 0.018202 |
| 6.22 | 0.045414 | 0.745517 | 0.195632 | 0.013437 | 0.031831 |
| 6.2 | 0.071109 | 0.886488 | 0.042403 | 0 | 0.027537 |
| 6.19 | 0.040779 | 0.952662 | 0 | 0.006559 | 0.231493 |
| 6.19 | 0.067538 | 0.901478 | 0.024953 | 0.00603 | 0.018914 |
| 6.16 | 0.083189 | 0.898119 | 0.012762 | 0.00593 | 0.16835 |
| 6.15 | 0.029173 | 0.847523 | 0.123304 | 0 | 0.02368 |
| 6.08 | 0.058289 | 0.8764 | 0.065311 | 0 | 0.202343 |
| 6.07 | 0.039863 | 0.711922 | 0.248216 | 0 | 0.020078 |
| 6.07 | 0.03505 | 0.940453 | 0 | 0.024497 | 0.000582 |
| 6.03 | 0.043989 | 0.711189 | 0.244822 | 0 | 0.171178 |
| 6.01 | 0.019964 | 0.944058 | 0.034331 | 0.001647 | 0.054066 |
| 6.01 | 0.046376 | 0.702486 | 0.251138 | 0 | 0.003461 |
| 6.01 | 0.033553 | 0.805444 | 0.155166 | 0.005836 | 0.089808 |
| 6 | 0.063452 | 0.859274 | 0.077275 | 0 | 0.167713 |
| 5.95 | 0.224025 | 0.744055 | 0.03192 | 0 | 0.080825 |
| 5.95 | 0.039 | 0.770268 | 0.167469 | 0.023263 | 0.142616 |
| 5.9 | 0.035057 | 0.565328 | 0.393756 | 0.005859 | 0.098151 |
| 5.88 | 0.072101 | 0.86385 | 0.054567 | 0.009482 | 0.064817 |
| 5.85 | 0.016337 | 0.866699 | 0.111594 | 0.00537 | 0.013295 |
| 5.84 | 0.035743 | 0.671162 | 0.284171 | 0.008924 | 0.015164 |
| 5.84 | 0.019597 | 0.789053 | 0.19135 | 0 | 0.007049 |
| 5.84 | 0.067136 | 0.715851 | 0.217013 | 0 | 0.03475 |
| 5.83 | 0.032002 | 0.781137 | 0.152364 | 0.034498 | 0.000807 |
| 5.8 | 0.041854 | 0.859523 | 0.094217 | 0.004407 | 0.152709 |
| 5.79 | 0.091309 | 0.876741 | 0.03195 | 0 | 0.02242 |
| 5.76 | 0.008343 | 0.991657 | 0 | 0 | 0.039714 |
| 5.74 | 0.098285 | 0.880774 | 0.020941 | 0 | 0.173938 |
| 5.73 | 0.049996 | 0.803418 | 0.146586 | 0 | 0.137218 |
| 5.73 | 0.034667 | 0.836308 | 0.12772 | 0.001305 | 0.026518 |
| 5.67 | 0.052255 | 0.898211 | 0.046067 | 0.003468 | 0.000502 |
| 5.66 | 0.01264 | 0.937165 | 0.043062 | 0.007133 | 0.185625 |
| 5.61 | 0.072848 | 0.708754 | 0.212019 | 0.006379 | 0.2346 |
| 5.59 | 0.030356 | 0.662937 | 0.306707 | 0 | 0.021427 |
| 5.55 | 0.041847 | 0.933975 | 0 | 0.024178 | 0.027727 |
| 5.49 | 0.078413 | 0.768356 | 0.153232 | 0 | 0.086105 |
| 5.47 | 0.032047 | 0.620483 | 0.34747 | 0 | 0.032438 |
| 5.43 | 0.031001 | 0.661591 | 0.277932 | 0.029476 | 0.000647 |
| 5.41 | 0.008458 | 0.766388 | 0.13723 | 0.087924 | 0.073515 |
| 5.32 | 0.064139 | 0.813701 | 0.122161 | 0 | 0.111806 |
| 5.29 | 0.018223 | 0.50482 | 0.425159 | 0 | 0.016896 |
| 5.27 | 0.105629 | 0.788264 | 0.072893 | 0.033215 | 0.044414 |
| 5.25 | 0.049416 | 0.637355 | 0.298827 | 0.014403 | 0.051553 |
| 5.25 | 0.022113 | 0.740767 | 0.237121 | 0 | 0.271627 |
| 5.24 | 0.014656 | 0.937846 | 0.041141 | 0.006357 | 0.017017 |
| 5.18 | 0.023913 | 0.904383 | 0.050222 | 0.021483 | 0.365279 |
| 5.07 | 0.032845 | 0.784505 | 0.155122 | 0.027527 | 0.09242 |
| 5.06 | 0.180694 | 0.813429 | 0 | 0.005877 | 0.020059 |
| 5.02 | 0.009634 | 0.907754 | 0.082612 | 0 | 0.016301 |
| 5.01 | 0.077048 | 0.723402 | 0.19955 | 0 | 0.077261 |
| 5 | 0.010983 | 0.903515 | 0.085502 | 0 | 0.036375 |
| 4.99 | 0.101064 | 0.634403 | 0.264178 | 0.000356 | 0.002776 |
| 4.96 | 0.04777 | 0.769868 | 0.182362 | 0 | 0.025439 |
| 4.9 | 0.056263 | 0.943737 | 0 | 0 | 0.41748 |
| 4.89 | 0.139615 | 0.679458 | 0.17389 | 0.007037 | 0.000136 |
| 4.88 | 0.029573 | 0.84438 | 0.121215 | 0.004832 | 0.031662 |
| 4.88 | 0.03865 | 0.910378 | 0.050972 | 0 | 0.003528 |
| 4.84 | 0.028722 | 0.608216 | 0.319598 | 0.043464 | 0.002594 |
| 4.84 | 0.028701 | 0.880606 | 0.090693 | 0 | 0.1192 |
| 4.76 | 0.01259 | 0.695308 | 0.274294 | 0.017807 | 0.002173 |
| 4.72 | 0.044084 | 0.893109 | 0.062806 | 0 | 0.010772 |
| 4.72 | 0.01221 | 0.707236 | 0.280555 | 0 | 0.010554 |
| 4.69 | 0.077148 | 0.72609 | 0.196763 | 0 | 0.022359 |
| 4.64 | 0.042009 | 0.716236 | 0.241755 | 0 | 0.25503 |
| 4.6 | 0.021323 | 0.624195 | 0.354482 | 0 | 0.002746 |
| 4.59 | 0.077189 | 0.793972 | 0.122556 | 0.006282 | 0.067732 |
| 4.55 | 0.012158 | 0.877799 | 0.104953 | 0.00509 | 0.119091 |
| 4.53 | 0.279126 | 0.620004 | 0.100869 | 0 | 0.032547 |
| 4.49 | 0.021006 | 0.91466 | 0.064334 | 0 | 0.031401 |
| 4.44 | 0.031151 | 0.746825 | 0.203347 | 0.018677 | 0.101497 |
| 4.42 | 0.026963 | 0.570467 | 0.183307 | 0.219263 | 0.0174 |
| 4.4 | 0.167614 | 0.459503 | 0.216179 | 0.156704 | 0.034594 |
| 4.34 | 0.03717 | 0.760903 | 0.201927 | 0 | 0.006919 |
| 4.3 | 0.027512 | 0.92664 | 0.024607 | 0.02124 | 0.166807 |
| 4.27 | 0.037419 | 0.923312 | 0.010583 | 0.028685 | 0.061092 |
| 4.22 | 0.030852 | 0.937414 | 0.022909 | 0.008825 | 0.063511 |
| 4.17 | 0.020372 | 0.836787 | 0.071528 | 0.071313 | 0.01532 |
| 4.17 | 0.037805 | 0.73086 | 0.231335 | 0 | 0.156062 |
| 4.17 | 0.0274 | 0.680913 | 0.280471 | 0.011216 | 0.088547 |
| 4.12 | 0.029603 | 0.751571 | 0.218827 | 0 | 0.079736 |
| 4.12 | 0.025682 | 0.856233 | 0.069107 | 0.048978 | 0.562058 |
| 4.11 | 0.041595 | 0.871269 | 0.087137 | 0 | 0.085193 |
| 4.1 | 0.042971 | 0.949884 | 0.007145 | 0 | 0.291543 |
| 4.1 | 0.072147 | 0.370505 | 0.269155 | 0.273316 | 0.041596 |
| 4.09 | 0.081238 | 0.718524 | 0.200238 | 0 | 0.013759 |
| 4.08 | 0.011622 | 0.828127 | 0.160251 | 0 | 0.020163 |
| 4.07 | 0.049856 | 0.810036 | 0.140108 | 0 | 0.019612 |
| 4.05 | 0.045204 | 0.596497 | 0.333565 | 0.01242 | 0.118649 |
| 4 | 0.049178 | 0.932341 | 0.018482 | 0 | 0.034648 |
| 3.94 | 0.074414 | 0.869734 | 0.019153 | 0.036699 | 0.003266 |
| 3.94 | 0.017442 | 0.520835 | 0.389462 | 0.072261 | 0.001443 |
| 3.9 | 0.069932 | 0.770161 | 0.159907 | 0 | 0.300129 |
| 3.9 | 0.099347 | 0.900653 | 0 | 0 | 0.147427 |
| 3.89 | 0.061675 | 0.89241 | 0.045559 | 0.000356 | 0.165958 |
| 3.88 | 0.013546 | 0.804015 | 0.125482 | 0.056958 | 0.043361 |
| 3.78 | 0.032579 | 0.759046 | 0.200145 | 0.008229 | 0.014697 |
| 3.75 | 0.022281 | 0.817366 | 0.144456 | 0.015897 | 0.002968 |
| 3.74 | 0.073048 | 0.7756 | 0.151352 | 0 | 0.061654 |
| 3.74 | 0.023466 | 0.646274 | 0.253975 | 0.066486 | 0.024883 |
| 3.74 | 0.08742 | 0.91258 | 0 | 0 | 0.040064 |
| 3.73 | 0.002975 | 0.947564 | 0.037218 | 0.012244 | 0.071134 |
| 3.71 | 0.030564 | 0.913953 | 0.055484 | 0 | 0.087083 |
| 3.69 | 0.038 | 0.931754 | 0 | 0.030246 | 7.47E-05 |
| 3.67 | 0.103313 | 0.732831 | 0.163856 | 0 | 0.045637 |
| 3.62 | 0.502659 | 0.453471 | 0.003471 | 0.040398 | 0.154066 |
| 3.48 | 0.12639 | 0.853682 | 0.019928 | 0 | 0.018693 |
| 3.42 | 0.029925 | 0.788213 | 0.181862 | 0 | 0.020835 |
| 3.29 | 0.04474 | 0.643511 | 0.311749 | 0 | 0.033318 |
| 3.23 | 0.070667 | 0.90593 | 0.02212 | 0.001283 | 0.083461 |
| 3.16 | 0.024747 | 0.964457 | 0.010796 | 0 | 0.256071 |
| 3.15 | 0.018798 | 0.873342 | 0.096714 | 0.011147 | 0.000615 |
| 3.12 | 0.017962 | 0.804942 | 0.163669 | 0.013427 | 0.006701 |
| 3.12 | 0.038505 | 0.828164 | 0.133331 | 0 | 0.114387 |
| 3.06 | 0.007157 | 0.760099 | 0.232744 | 0 | 0.138899 |
| 2.91 | 0.026149 | 0.925556 | 0.048294 | 0 | 0.048499 |
| 2.89 | 0.035536 | 0.632685 | 0.323556 | 0.008224 | 0.021802 |
| 2.8 | 0.086829 | 0.552521 | 0.36065 | 0 | 0.123484 |
| 2.66 | 0.032119 | 0.666449 | 0.28536 | 0.016073 | 0.089523 |
| 2.62 | 0.096755 | 0.818844 | 0.082158 | 0.002244 | 0.031972 |
| 2.48 | 0.079122 | 0.728452 | 0.189573 | 0.002853 | 0.013886 |
| 2.4 | 0.016859 | 0.887013 | 0.096128 | 0 | 0.07925 |
| 2.32 | 0.177178 | 0.698147 | 0.059042 | 0.065633 | 0.169988 |
| 2.17 | 0.028708 | 0.609643 | 0.361648 | 0 | 0.082055 |
| 2.16 | 0.019281 | 0.635579 | 0.264739 | 0.080401 | 0.007959 |
| 2.13 | 0.09225 | 0.835858 | 0.071892 | 0 | 0.064066 |
| 2.12 | 0.070053 | 0.928604 | 0.001343 | 0 | 0.237388 |
| 2.11 | 0.050098 | 0.942054 | 0.007554 | 0.000294 | 0.032512 |
| 2.04 | 0.030802 | 0.910639 | 0.052474 | 0.006085 | 0.34264 |
| 2.01 | 0.02837 | 0.674304 | 0.282227 | 0.015099 | 0.162311 |
| 1.98 | 0.041492 | 0.83554 | 0.122968 | 0 | 0.236861 |
| 1.91 | 0.027594 | 0.755788 | 0.216618 | 0 | 0.07257 |
| 1.84 | 0.04742 | 0.747043 | 0.205537 | 0 | 0.027979 |
| 1.83 | 0.02633 | 0.705616 | 0.268053 | 0 | 3.04E-06 |
| 1.49 | 0.048003 | 0.729414 | 0.209387 | 0.013197 | 0.033985 |
| 1.18 | 0.020858 | 0.994368 | 0.0019 | 0 | 0.003386 |
| 0.98 | 0.0432 | 0.9568 | 0 | 0 | 0.013619 |

*Racoon dog*

| Harvest  (ind./100ha) | SETTLEMENTS  (%) | CROPS  (%) | FORESTS  (%) | WATER  (%) | WIND FARM  (%) |
| --- | --- | --- | --- | --- | --- |
| 17.88 | 0.086482 | 0.913518 | 0 | 0 | 0.005667 |
| 15.01 | 0.058012 | 0.561444 | 0.380544 | 0 | 0.028502 |
| 12.85 | 0.028499 | 0.562559 | 0.360137 | 0.048805 | 0.001166 |
| 11.58 | 0.032845 | 0.784505 | 0.155122 | 0.027527 | 0.09242 |
| 11.28 | 0.028434 | 0.931806 | 0.027306 | 0.012453 | 0.127255 |
| 10.2 | 0.030872 | 0.686417 | 0.272097 | 0.010614 | 0.010509 |
| 9.87 | 0.021317 | 0.804667 | 0.174016 | 0 | 0.278978 |
| 9.83 | 0.060716 | 0.682299 | 0.256985 | 0 | 0.053094 |
| 9.69 | 0.029447 | 0.903798 | 0.066755 | 0 | 0.23376 |
| 9.55 | 0.025498 | 0.828399 | 0.114062 | 0.032041 | 0.078826 |
| 8.73 | 0.073033 | 0.813903 | 0.097747 | 0.015317 | 0.19531 |
| 8.14 | 0.130716 | 0.674756 | 0.194527 | 0 | 0.138547 |
| 7.99 | 0.095858 | 0.675914 | 0.223198 | 0.005031 | 0.017825 |
| 7.86 | 0.050548 | 0.895549 | 0.053477 | 0.000426 | 0.040513 |
| 7.68 | 0.037255 | 0.909058 | 0.053687 | 0 | 0.110157 |
| 7.64 | 0.012081 | 0.564216 | 0.390193 | 0.03351 | 0.035279 |
| 7.48 | 0.024205 | 0.53313 | 0.202177 | 0.240487 | 0.002058 |
| 6.81 | 0.108022 | 0.691244 | 0.180332 | 0.020402 | 0.004315 |
| 6.79 | 0.025165 | 0.709589 | 0.265246 | 0 | 0.340679 |
| 6.75 | 0.092208 | 0.70089 | 0.190913 | 0.015988 | 0.053543 |
| 6.67 | 0.102583 | 0.897417 | 0 | 0 | 0.045341 |
| 6.62 | 0.134972 | 0.724281 | 0.120111 | 0.020636 | 0.008062 |
| 6.51 | 0.059836 | 0.773228 | 0.059752 | 0.107184 | 0.135669 |
| 6.33 | 0.026258 | 0.530627 | 0.436197 | 0.006918 | 0.028396 |
| 6.18 | 0.052284 | 0.743996 | 0.193735 | 0.009985 | 0.045387 |
| 6.09 | 0.035435 | 0.823689 | 0.105185 | 0.035691 | 0.047584 |
| 6.05 | 0.034088 | 0.734203 | 0.231709 | 0 | 0.003313 |
| 5.96 | 0.037227 | 0.774049 | 0.188724 | 0 | 0.136711 |
| 5.83 | 0.011822 | 0.922221 | 0.0577 | 0.008256 | 0.027789 |
| 5.81 | 0.181455 | 0.617007 | 0.187585 | 0.013953 | 0.12495 |
| 5.78 | 0.012366 | 0.881566 | 0.053689 | 0.052379 | 0.104641 |
| 5.66 | 0.108214 | 0.736279 | 0.126343 | 0.029164 | 0.000839 |
| 5.66 | 0.02159 | 0.743128 | 0.235281 | 0 | 0.058284 |
| 5.62 | 0.064454 | 0.807537 | 0.118761 | 0.009248 | 0.039809 |
| 5.54 | 0.06371 | 0.687375 | 0.248915 | 0 | 0.024296 |
| 5.53 | 0.052708 | 0.717515 | 0.2169 | 0.012876 | 0.308219 |
| 5.35 | 0.054088 | 0.749397 | 0.163075 | 0.03344 | 0.116868 |
| 5.1 | 0.03957 | 0.723527 | 0.232789 | 0.004115 | 0.000244 |
| 5.03 | 0.048432 | 0.813801 | 0.137767 | 0 | 0.016043 |
| 5 | 0.031111 | 0.552667 | 0.394879 | 0.021342 | 0.026238 |
| 4.91 | 0.02225 | 0.900537 | 0.067932 | 0.00928 | 0.000577 |
| 4.82 | 0.130492 | 0.869508 | 0 | 0 | 0.036249 |
| 4.67 | 0.07946 | 0.663096 | 0.257444 | 0 | 0.119837 |
| 4.66 | 0.064382 | 0.647883 | 0.274131 | 0.013604 | 0.054466 |
| 4.58 | 0.054568 | 0.700053 | 0.236636 | 0.008742 | 0.051978 |
| 4.48 | 0.041708 | 0.943745 | 0 | 0.014547 | 0.008971 |
| 4.28 | 0.039287 | 0.659343 | 0.239964 | 0.061407 | 0.021402 |
| 4.24 | 0.043831 | 0.54447 | 0.298957 | 0.112741 | 0.002629 |
| 4.23 | 0.01082 | 0.979523 | 0 | 0.009656 | 0.210532 |
| 4.22 | 0.025413 | 0.787728 | 0.186858 | 0 | 0.021162 |
| 4.17 | 0.08025 | 0.857246 | 0.062497 | 7.34E-06 | 0.108955 |
| 4.17 | 0.051785 | 0.901516 | 0.046699 | 0 | 0.007171 |
| 4.17 | 0.18621 | 0.781401 | 0.032389 | 0 | 0.147147 |
| 4.16 | 0.077519 | 0.685448 | 0.237032 | 0 | 0.018202 |
| 4.15 | 0.066768 | 0.576755 | 0.179528 | 0.176949 | 0.046495 |
| 4.14 | 0.137764 | 0.692755 | 0.169481 | 0 | 0.01181 |
| 3.98 | 0.014883 | 0.952852 | 0.026053 | 0.006211 | 0.039717 |
| 3.86 | 0.036312 | 0.904341 | 0.024842 | 0.034504 | 0.160208 |
| 3.8 | 0.001377 | 0.682013 | 0.307943 | 0.008666 | 0.024811 |
| 3.76 | 0.085555 | 0.904837 | 0.009608 | 0 | 0.034194 |
| 3.74 | 0.073048 | 0.7756 | 0.151352 | 0 | 0.061654 |
| 3.73 | 0.020053 | 0.595764 | 0.384183 | 0 | 0.022035 |
| 3.73 | 0.041354 | 0.793022 | 0.109663 | 0.055961 | 0.156689 |
| 3.7 | 0.041239 | 0.678391 | 0.280371 | 0 | 0.166558 |
| 3.68 | 0.081238 | 0.718524 | 0.200238 | 0 | 0.013759 |
| 3.62 | 0.009221 | 0.887556 | 0.073236 | 0.029987 | 0.245076 |
| 3.56 | 0.077906 | 0.776797 | 0.145296 | 0 | 0.026626 |
| 3.53 | 0.043407 | 0.941455 | 0.015138 | 0 | 0.002294 |
| 3.53 | 0.098187 | 0.786894 | 0.114919 | 0 | 0.033695 |
| 3.51 | 0.017027 | 0.946215 | 0.036758 | 0 | 0.005504 |
| 3.51 | 0.019597 | 0.789053 | 0.19135 | 0 | 0.007049 |
| 3.39 | 0.027274 | 0.638686 | 0.329299 | 0.004741 | 0.087119 |
| 3.38 | 0.043339 | 0.663185 | 0.278789 | 0.014687 | 0.102922 |
| 3.37 | 0.039445 | 0.880548 | 0.078509 | 0.001499 | 0.00146 |
| 3.37 | 0.027304 | 0.917848 | 0.04837 | 0.006477 | 0.349861 |
| 3.35 | 0.012289 | 0.967888 | 0.019822 | 0 | 0.169411 |
| 3.31 | 0.03398 | 0.965065 | 0.000956 | 0 | 0.457655 |
| 3.31 | 0.04557 | 0.58709 | 0.36734 | 0 | 0.070527 |
| 3.29 | 0.097723 | 0.899411 | 0.002866 | 0 | 0.018141 |
| 3.25 | 0.033665 | 0.827557 | 0.095811 | 0.042966 | 0.085835 |
| 3.22 | 0.022756 | 0.827933 | 0.149312 | 0 | 0.036017 |
| 3.19 | 0.096072 | 0.863331 | 0.040596 | 0 | 0.08757 |
| 3.19 | 0.036625 | 0.770464 | 0.167258 | 0.025652 | 0.178928 |
| 3.18 | 0.018482 | 0.571366 | 0.403494 | 0.006658 | 0.08103 |
| 3.16 | 0.027717 | 0.596513 | 0.370443 | 0.005327 | 0.035165 |
| 3.15 | 0.014978 | 0.876519 | 0.099945 | 0.008559 | 0.01608 |
| 3.15 | 0.022113 | 0.740767 | 0.237121 | 0 | 0.271627 |
| 3.13 | 0.036113 | 0.84386 | 0.028627 | 0.0914 | 0.01802 |
| 3.13 | 0.03309 | 0.827144 | 0.139765 | 0 | 0.030663 |
| 3.12 | 0.072848 | 0.708754 | 0.212019 | 0.006379 | 0.2346 |
| 3.09 | 0.042009 | 0.716236 | 0.241755 | 0 | 0.25503 |
| 3.08 | 0.072147 | 0.370505 | 0.269155 | 0.273316 | 0.041596 |
| 3.02 | 0.029025 | 0.753063 | 0.182242 | 0.03567 | 0.000693 |
| 3 | 0.049178 | 0.932341 | 0.018482 | 0 | 0.034648 |
| 3 | 0.063452 | 0.859274 | 0.077275 | 0 | 0.167713 |
| 3 | 0.008526 | 0.940563 | 0.050911 | 0 | 0.124726 |
| 2.99 | 0.033849 | 0.683169 | 0.28066 | 0.002321 | 0.049347 |
| 2.98 | 0.050363 | 0.949637 | 0 | 0 | 0.000103 |
| 2.97 | 0.137039 | 0.844241 | 0.01872 | 0 | 0.054031 |
| 2.96 | 0.053113 | 0.649051 | 0.261498 | 0.036338 | 0.042373 |
| 2.95 | 0.017478 | 0.896971 | 0.020908 | 0.064643 | 0.124726 |
| 2.94 | 0.110639 | 0.740989 | 0.108626 | 0.039746 | 0.079146 |
| 2.93 | 0.04838 | 0.768229 | 0.137646 | 0.045745 | 0.000874 |
| 2.92 | 0.058187 | 0.528333 | 0.413479 | 0 | 0.002676 |
| 2.9 | 0.041854 | 0.859523 | 0.094217 | 0.004407 | 0.152709 |
| 2.9 | 0.032721 | 0.728697 | 0.238522 | 5.97E-05 | 0.018052 |
| 2.89 | 0.018063 | 0.899386 | 0.044394 | 0.038157 | 0.085235 |
| 2.89 | 0.04551 | 0.731711 | 0.222779 | 0 | 0.007162 |
| 2.87 | 0.053881 | 0.915746 | 0.006846 | 0.023526 | 0.034166 |
| 2.87 | 0.010053 | 0.727504 | 0.207121 | 0.055322 | 0.048494 |
| 2.87 | 0.026162 | 0.710156 | 0.240745 | 0.022937 | 0.031545 |
| 2.77 | 0.049931 | 0.628573 | 0.321228 | 0.000269 | 0.036331 |
| 2.76 | 0.03505 | 0.940453 | 0 | 0.024497 | 0.000582 |
| 2.75 | 0.095985 | 0.700639 | 0.198975 | 0.0044 | 0.02055 |
| 2.75 | 0.119335 | 0.541145 | 0.336507 | 0.003013 | 0.018434 |
| 2.75 | 0.029603 | 0.751571 | 0.218827 | 0 | 0.079736 |
| 2.74 | 0.025046 | 0.688707 | 0.286247 | 0 | 0.026522 |
| 2.74 | 0.066093 | 0.856123 | 0.077784 | 0 | 0.032206 |
| 2.74 | 0.049055 | 0.900592 | 0.050353 | 0 | 0.099 |
| 2.73 | 0.029925 | 0.788213 | 0.181862 | 0 | 0.020835 |
| 2.72 | 0.019528 | 0.767714 | 0.212758 | 0 | 0.030766 |
| 2.7 | 0.030193 | 0.919865 | 0.019158 | 0.030784 | 0.093644 |
| 2.65 | 0.036323 | 0.95832 | 0.005356 | 0 | 0.348035 |
| 2.65 | 0.055185 | 0.873829 | 0.070986 | 0 | 0.060068 |
| 2.62 | 0.046998 | 0.826689 | 0.126312 | 0 | 0.087959 |
| 2.6 | 0.111642 | 0.775675 | 0.112682 | 0 | 0.047805 |
| 2.57 | 0.100035 | 0.878576 | 0 | 0.021389 | 0.004507 |
| 2.55 | 0.059243 | 0.805019 | 0.135738 | 0 | 0.094313 |
| 2.54 | 0.059469 | 0.940531 | 0 | 0 | 0.036402 |
| 2.5 | 0.101064 | 0.634403 | 0.264178 | 0.000356 | 0.002776 |
| 2.5 | 0.034773 | 0.709889 | 0.247028 | 0.008309 | 0.059819 |
| 2.49 | 0.041745 | 0.712863 | 0.243395 | 0.001997 | 0.02306 |
| 2.48 | 0.134499 | 0.702936 | 0.162566 | 0 | 0.002641 |
| 2.47 | 0.025682 | 0.856233 | 0.069107 | 0.048978 | 0.562058 |
| 2.45 | 0.01756 | 0.936756 | 0.045684 | 0 | 0.587545 |
| 2.44 | 0.045934 | 0.924793 | 0.02209 | 0.007184 | 0.19778 |
| 2.44 | 0.042169 | 0.597119 | 0.360712 | 0 | 0.036652 |
| 2.44 | 0.028579 | 0.779842 | 0.184295 | 0.007283 | 0.00428 |
| 2.43 | 0.051852 | 0.630668 | 0.304929 | 0.01255 | 0.049537 |
| 2.43 | 0.035856 | 0.89092 | 0.073224 | 0 | 0.152419 |
| 2.4 | 0.078715 | 0.778306 | 0.133458 | 0.009521 | 0.022315 |
| 2.4 | 0.016859 | 0.887013 | 0.096128 | 0 | 0.07925 |
| 2.36 | 0.141596 | 0.638012 | 0.156134 | 0.064258 | 0.108228 |
| 2.35 | 0.015126 | 0.759174 | 0.204304 | 0.021397 | 0.097869 |
| 2.35 | 0.034684 | 0.90422 | 0.061095 | 0 | 0.079926 |
| 2.33 | 0.031482 | 0.918601 | 0 | 0.049917 | 0.016405 |
| 2.32 | 0.046989 | 0.861338 | 0.091672 | 0 | 0.288311 |
| 2.29 | 0.03717 | 0.760903 | 0.201927 | 0 | 0.006919 |
| 2.28 | 0.046144 | 0.680903 | 0.254461 | 0.018493 | 0.000879 |
| 2.28 | 0.068708 | 0.849552 | 0.042768 | 0.038973 | 0.074069 |
| 2.24 | 0.065773 | 0.829725 | 0.103836 | 0.000666 | 0.181817 |
| 2.24 | 0.139056 | 0.729618 | 0.11691 | 0.014416 | 2.54E-05 |
| 2.22 | 0.12475 | 0.506296 | 0.368954 | 0 | 0.018843 |
| 2.19 | 0.011426 | 0.976589 | 0.011985 | 0 | 0.007056 |
| 2.19 | 0.068853 | 0.901758 | 0.029389 | 0 | 0.153666 |
| 2.19 | 0.040076 | 0.833117 | 0.087333 | 0.039474 | 0.241711 |
| 2.16 | 0.069142 | 0.772286 | 0.14675 | 0.011822 | 0.039618 |
| 2.15 | 0.027512 | 0.92664 | 0.024607 | 0.02124 | 0.166807 |
| 2.15 | 0.034667 | 0.836308 | 0.12772 | 0.001305 | 0.026518 |
| 2.15 | 0.069289 | 0.841813 | 0.088899 | 0 | 0.074837 |
| 2.13 | 0.112847 | 0.540884 | 0.2825 | 0.063769 | 0.234756 |
| 2.11 | 0.034493 | 0.812457 | 0.153051 | 0 | 0.073334 |
| 2.1 | 0.049416 | 0.637355 | 0.298827 | 0.014403 | 0.051553 |
| 2.1 | 0.017442 | 0.520835 | 0.389462 | 0.072261 | 0.001443 |
| 2.09 | 0.033275 | 0.900866 | 0.060343 | 0.005515 | 0.091831 |
| 2.09 | 0.031613 | 0.961818 | 0.003158 | 0.003411 | 0.046069 |
| 2.09 | 0.070047 | 0.841396 | 0.083412 | 0.005146 | 0.038956 |
| 2.09 | 0.077451 | 0.901627 | 0.020923 | 0 | 0.037246 |
| 2.09 | 0.025726 | 0.568486 | 0.358865 | 0.046922 | 0.138587 |
| 2.08 | 0.015311 | 0.850012 | 0.134677 | 0 | 0.043252 |
| 2.06 | 0.035595 | 0.757742 | 0.206663 | 0 | 0.026593 |
| 2.04 | 0.039022 | 0.789893 | 0.171085 | 0 | 0.011916 |
| 2.03 | 0.048241 | 0.890315 | 0.061444 | 0 | 0.004877 |
| 2.03 | 0.058289 | 0.8764 | 0.065311 | 0 | 0.202343 |
| 2.01 | 0.02837 | 0.674304 | 0.282227 | 0.015099 | 0.162311 |
| 2 | 0.013851 | 0.854947 | 0.094772 | 0.036431 | 0.005141 |
| 2 | 0.0274 | 0.680913 | 0.280471 | 0.011216 | 0.088547 |
| 1.97 | 0.039363 | 0.856503 | 0.095384 | 0.00875 | 0.023128 |
| 1.95 | 0.050611 | 0.799196 | 0.130295 | 0.019897 | 0.045145 |
| 1.92 | 0.124811 | 0.814342 | 0.060847 | 0 | 0.105066 |
| 1.91 | 0.006401 | 0.762863 | 0.215353 | 0.015384 | 0.120429 |
| 1.91 | 0.01712 | 0.800569 | 0.132823 | 0.049489 | 0.06699 |
| 1.91 | 0.025069 | 0.954991 | 0.01994 | 0 | 0.029211 |
| 1.9 | 0.021237 | 0.959433 | 0.019331 | 0 | 0.256702 |
| 1.88 | 0.020569 | 0.771368 | 0.155748 | 0.052315 | 0.015713 |
| 1.88 | 0.004494 | 0.931113 | 0.025152 | 0.03924 | 0.009948 |
| 1.88 | 0.030385 | 0.902018 | 0.026566 | 0.041031 | 0.017606 |
| 1.88 | 0.027328 | 0.959282 | 0.005139 | 0.008252 | 0.023328 |
| 1.86 | 0.029175 | 0.877794 | 0.093031 | 0 | 0.015471 |
| 1.85 | 0.069296 | 0.774471 | 0.070706 | 0.085528 | 0.031938 |
| 1.85 | 0.041847 | 0.933975 | 0 | 0.024178 | 0.027727 |
| 1.85 | 0.053764 | 0.773062 | 0.173173 | 0 | 0.151376 |
| 1.84 | 0.04601 | 0.785236 | 0.14245 | 0.026304 | 0.131842 |
| 1.83 | 0.045204 | 0.596497 | 0.333565 | 0.01242 | 0.118649 |
| 1.82 | 0.065419 | 0.70728 | 0.227301 | 0 | 0.288139 |
| 1.81 | 0.007743 | 0.882418 | 0.104562 | 0.005277 | 0.030295 |
| 1.81 | 0.168176 | 0.81787 | 0.013954 | 0 | 0.049296 |
| 1.81 | 0.043989 | 0.711189 | 0.244822 | 0 | 0.171178 |
| 1.81 | 0.030907 | 0.705282 | 0.26381 | 0 | 0.210419 |
| 1.81 | 0.031001 | 0.661591 | 0.277932 | 0.029476 | 0.000647 |
| 1.8 | 0.222442 | 0.74335 | 0.034208 | 0 | 0.006338 |
| 1.79 | 0.043956 | 0.854424 | 0.090371 | 0.011248 | 0.042874 |
| 1.78 | 0.045885 | 0.95365 | 0.000465 | 0 | 0.003323 |
| 1.77 | 0.080504 | 0.831155 | 0.005685 | 0.082657 | 0.041065 |
| 1.76 | 0.083189 | 0.898119 | 0.012762 | 0.00593 | 0.16835 |
| 1.75 | 0.041492 | 0.83554 | 0.122968 | 0 | 0.236861 |
| 1.73 | 0.007404 | 0.887462 | 0.105134 | 0 | 0.179264 |
| 1.73 | 0.038604 | 0.954512 | 0.006884 | 0 | 0.02608 |
| 1.71 | 0.21499 | 0.702711 | 0.080053 | 0.002246 | 0.007246 |
| 1.7 | 0.036485 | 0.81515 | 0.148365 | 0 | 0.19155 |
| 1.68 | 0.035057 | 0.565328 | 0.393756 | 0.005859 | 0.098151 |
| 1.67 | 0.010983 | 0.903515 | 0.085502 | 0 | 0.036375 |
| 1.66 | 0.025748 | 0.720481 | 0.253771 | 0 | 0.036214 |
| 1.66 | 0.153329 | 0.620561 | 0.18755 | 0.031629 | 0.091145 |
| 1.64 | 0.023232 | 0.748449 | 0.204923 | 0.023397 | 0.029774 |
| 1.63 | 0.139615 | 0.679458 | 0.17389 | 0.007037 | 0.000136 |
| 1.63 | 0.080689 | 0.873851 | 0.041035 | 0.004425 | 0.180591 |
| 1.62 | 0.009555 | 0.979002 | 0.000198 | 0.011245 | 0.076538 |
| 1.62 | 0.02289 | 0.955775 | 0.021335 | 0 | 0.120855 |
| 1.62 | 0.039863 | 0.711922 | 0.248216 | 0 | 0.020078 |
| 1.62 | 0.061571 | 0.728748 | 0.209681 | 0 | 0.041562 |
| 1.61 | 0.074589 | 0.640797 | 0.26404 | 0.020574 | 0.021459 |
| 1.61 | 0.203607 | 0.526209 | 0.270184 | 0 | 0.050672 |
| 1.59 | 0.03372 | 0.954883 | 0.011397 | 0 | 0.059214 |
| 1.59 | 0.01259 | 0.695308 | 0.274294 | 0.017807 | 0.002173 |
| 1.58 | 0.024747 | 0.964457 | 0.010796 | 0 | 0.256071 |
| 1.57 | 0.157547 | 0.729757 | 0.10907 | 0.003625 | 0.027969 |
| 1.56 | 0.010679 | 0.952 | 0.001661 | 0.035661 | 0.004639 |
| 1.56 | 0.077148 | 0.72609 | 0.196763 | 0 | 0.022359 |
| 1.53 | 0.044407 | 0.778417 | 0.176709 | 0.000467 | 0.032712 |
| 1.52 | 0.180694 | 0.813429 | 0 | 0.005877 | 0.020059 |
| 1.52 | 0.065776 | 0.916671 | 0.017553 | 0 | 0.021232 |
| 1.51 | 0.040823 | 0.959177 | 0 | 0 | 0.004214 |
| 1.5 | 0.074414 | 0.869734 | 0.019153 | 0.036699 | 0.003266 |
| 1.49 | 0.09225 | 0.835858 | 0.071892 | 0 | 0.064066 |
| 1.49 | 0.102823 | 0.881072 | 0.016105 | 0 | 0.048898 |
| 1.49 | 0.039739 | 0.691329 | 0.268932 | 0 | 0.195716 |
| 1.49 | 0.032367 | 0.938775 | 0.019579 | 0.009278 | 0.008665 |
| 1.48 | 0.063263 | 0.772515 | 0.155506 | 0.008716 | 0.17232 |
| 1.48 | 0.023913 | 0.904383 | 0.050222 | 0.021483 | 0.365279 |
| 1.48 | 0.036346 | 0.809759 | 0.128229 | 0.025666 | 0.037496 |
| 1.47 | 0.074807 | 0.798245 | 0.126948 | 0 | 0.052297 |
| 1.47 | 0.023883 | 0.761086 | 0.215031 | 0 | 0.033082 |
| 1.47 | 0.026963 | 0.570467 | 0.183307 | 0.219263 | 0.0174 |
| 1.47 | 0.086829 | 0.552521 | 0.36065 | 0 | 0.123484 |
| 1.46 | 0.032947 | 0.773658 | 0.145192 | 0.048202 | 0.049896 |
| 1.46 | 0.142246 | 0.407022 | 0.377298 | 0.073434 | 0.089493 |
| 1.46 | 0.067136 | 0.715851 | 0.217013 | 0 | 0.03475 |
| 1.45 | 0.048955 | 0.647511 | 0.303534 | 0 | 0.045488 |
| 1.45 | 0.028708 | 0.609643 | 0.361648 | 0 | 0.082055 |
| 1.44 | 0.072737 | 0.703643 | 0.172948 | 0.050672 | 0.003121 |
| 1.43 | 0.017507 | 0.974532 | 0.00609 | 0.001872 | 0.18464 |
| 1.43 | 0.049996 | 0.803418 | 0.146586 | 0 | 0.137218 |
| 1.42 | 0.029263 | 0.64246 | 0.263832 | 0.064444 | 0.068015 |
| 1.42 | 0.032579 | 0.759046 | 0.200145 | 0.008229 | 0.014697 |
| 1.4 | 0.007534 | 0.991543 | 0 | 0.000922 | 0.020689 |
| 1.39 | 0.035474 | 0.794716 | 0.16981 | 0 | 0.222865 |
| 1.38 | 0.060899 | 0.775398 | 0.163702 | 0 | 0.022238 |
| 1.37 | 0.041595 | 0.871269 | 0.087137 | 0 | 0.085193 |
| 1.37 | 0.072839 | 0.741172 | 0.185989 | 0 | 0.02913 |
| 1.36 | 0.046888 | 0.885726 | 0.036393 | 0.030993 | 0.012365 |
| 1.36 | 0.069932 | 0.770161 | 0.159907 | 0 | 0.300129 |
| 1.36 | 0.041801 | 0.944368 | 0 | 0.013831 | 0.010372 |
| 1.36 | 0.030864 | 0.930063 | 0 | 0.039073 | 0.003036 |
| 1.36 | 0.013546 | 0.804015 | 0.125482 | 0.056958 | 0.043361 |
| 1.35 | 0.003095 | 0.950925 | 0.015182 | 0.030799 | 0.103433 |
| 1.33 | 0.012828 | 0.801466 | 0.169907 | 0.015799 | 0.035618 |
| 1.33 | 0.022977 | 0.95925 | 0.006649 | 0.011124 | 0.025333 |
| 1.33 | 0.028999 | 0.817861 | 0.15314 | 0 | 0.078066 |
| 1.32 | 0.015465 | 0.974209 | 0.010326 | 0 | 0.001597 |
| 1.31 | 0.039787 | 0.928108 | 0.016795 | 0.015311 | 0.04215 |
| 1.28 | 0.009447 | 0.761519 | 0.21221 | 0.016824 | 0.01016 |
| 1.27 | 0.021306 | 0.96547 | 0.000434 | 0.01279 | 0.093825 |
| 1.27 | 0.052039 | 0.626445 | 0.321516 | 0 | 0.038994 |
| 1.27 | 0.030802 | 0.910639 | 0.052474 | 0.006085 | 0.34264 |
| 1.26 | 0.05913 | 0.575212 | 0.365601 | 5.72E-05 | 0.03847 |
| 1.26 | 0.096224 | 0.839476 | 0.0643 | 0 | 0.055361 |
| 1.26 | 0.110983 | 0.870325 | 0.018692 | 0 | 0.141334 |
| 1.26 | 0.029013 | 0.883584 | 0.082962 | 0.004441 | 0.000183 |
| 1.25 | 0.01512 | 0.858559 | 0.07309 | 0.053231 | 0.037677 |
| 1.25 | 0.041876 | 0.634991 | 0.323132 | 0 | 0.019803 |
| 1.25 | 0.077189 | 0.793972 | 0.122556 | 0.006282 | 0.067732 |
| 1.25 | 0.023466 | 0.646274 | 0.253975 | 0.066486 | 0.024883 |
| 1.25 | 0.046194 | 0.608006 | 0.345294 | 0.000506 | 0.002681 |
| 1.24 | 0.030564 | 0.913953 | 0.055484 | 0 | 0.087083 |
| 1.24 | 0.071109 | 0.886488 | 0.042403 | 0 | 0.027537 |
| 1.24 | 0.039215 | 0.907213 | 0.053573 | 0 | 0.070329 |
| 1.23 | 0.055552 | 0.69975 | 0.239609 | 0.005088 | 0.003034 |
| 1.23 | 0.038 | 0.931754 | 0 | 0.030246 | 7.47E-05 |
| 1.23 | 0.04306 | 0.876667 | 0.080273 | 0 | 0.034083 |
| 1.22 | 0.167614 | 0.459503 | 0.216179 | 0.156704 | 0.034594 |
| 1.22 | 0.029573 | 0.84438 | 0.121215 | 0.004832 | 0.031662 |
| 1.22 | 0.03865 | 0.910378 | 0.050972 | 0 | 0.003528 |
| 1.22 | 0.103313 | 0.732831 | 0.163856 | 0 | 0.045637 |
| 1.21 | 0.011447 | 0.645638 | 0.323308 | 0.019607 | 0.001809 |
| 1.21 | 0.03637 | 0.8723 | 0.091329 | 0 | 0.000179 |
| 1.2 | 0.058021 | 0.662699 | 0.258411 | 0.020869 | 0.038288 |
| 1.2 | 0.033553 | 0.805444 | 0.155166 | 0.005836 | 0.089808 |
| 1.19 | 0.216664 | 0.779225 | 0.004111 | 0 | 0.294511 |
| 1.18 | 0.072101 | 0.86385 | 0.054567 | 0.009482 | 0.064817 |
| 1.18 | 0.01221 | 0.707236 | 0.280555 | 0 | 0.010554 |
| 1.18 | 0.053152 | 0.914463 | 0.025729 | 0.006655 | 0.029585 |
| 1.18 | 0.021547 | 0.897054 | 0.081399 | 0 | 0.003304 |
| 1.18 | 0.046884 | 0.923363 | 0.029753 | 0 | 0.00521 |
| 1.17 | 0.029683 | 0.738291 | 0.186006 | 0.046019 | 0.017025 |
| 1.17 | 0.101092 | 0.659995 | 0.223647 | 0.015266 | 0.004566 |
| 1.17 | 0.042971 | 0.949884 | 0.007145 | 0 | 0.291543 |
| 1.17 | 0.016972 | 0.707628 | 0.225538 | 0.049862 | 0.111867 |
| 1.17 | 0.105751 | 0.72069 | 0.166077 | 0.007481 | 0.034231 |
| 1.16 | 0.12639 | 0.853682 | 0.019928 | 0 | 0.018693 |
| 1.14 | 0.063612 | 0.891342 | 0.045046 | 0 | 0.107764 |
| 1.14 | 0.039 | 0.770268 | 0.167469 | 0.023263 | 0.142616 |
| 1.14 | 0.072977 | 0.856731 | 0.070292 | 0 | 0.074098 |
| 1.13 | 0.059338 | 0.879605 | 0.056493 | 0 | 0.222732 |
| 1.13 | 0.008032 | 0.962155 | 0.004333 | 0.02548 | 0.026076 |
| 1.12 | 0.017755 | 0.694353 | 0.28756 | 0.000331 | 0.007311 |
| 1.12 | 0.009634 | 0.907754 | 0.082612 | 0 | 0.016301 |
| 1.12 | 0.08742 | 0.91258 | 0 | 0 | 0.040064 |
| 1.11 | 0.028831 | 0.777557 | 0.193612 | 0 | 0.01115 |
| 1.11 | 0 | 0.891838 | 0.108162 | 0 | 0.002346 |
| 1.1 | 0.032421 | 0.811665 | 0.155915 | 0 | 0.079009 |
| 1.1 | 0.079122 | 0.728452 | 0.189573 | 0.002853 | 0.013886 |
| 1.08 | 0.06005 | 0.93995 | 0 | 0 | 0.023202 |
| 1.08 | 0.02551 | 0.914172 | 0.045998 | 0.01432 | 0.02754 |
| 1.08 | 0.065527 | 0.728519 | 0.126969 | 0.078986 | 0.047136 |
| 1.07 | 0.04742 | 0.747043 | 0.205537 | 0 | 0.027979 |
| 1.07 | 0.059068 | 0.905269 | 0.035663 | 0 | 0.061176 |
| 1.06 | 0.045044 | 0.886899 | 0.026934 | 0.041122 | 0.00106 |
| 1.06 | 0.067538 | 0.901478 | 0.024953 | 0.00603 | 0.018914 |
| 1.04 | 0.098285 | 0.880774 | 0.020941 | 0 | 0.173938 |
| 1.02 | 0.021323 | 0.624195 | 0.354482 | 0 | 0.002746 |
| 1.01 | 0.014212 | 0.940359 | 0.02911 | 0.01632 | 0.085242 |
| 1.01 | 0.075708 | 0.831813 | 0.092479 | 0 | 0.125231 |
| 1 | 0.035743 | 0.671162 | 0.284171 | 0.008924 | 0.015164 |
| 1 | 0.049629 | 0.944396 | 0.005975 | 0 | 0.171729 |
| 0.99 | 0.037281 | 0.903436 | 0.042729 | 0.016554 | 0.047312 |
| 0.99 | 0.11287 | 0.864337 | 0.022793 | 0 | 0.094936 |
| 0.99 | 0.224025 | 0.744055 | 0.03192 | 0 | 0.080825 |
| 0.98 | 0.01216 | 0.758907 | 0.228932 | 0 | 0.023113 |
| 0.98 | 0.056263 | 0.943737 | 0 | 0 | 0.41748 |
| 0.97 | 0.028722 | 0.608216 | 0.319598 | 0.043464 | 0.002594 |
| 0.97 | 0.070667 | 0.90593 | 0.02212 | 0.001283 | 0.083461 |
| 0.96 | 0.086936 | 0.888209 | 0.024855 | 0 | 0.080122 |
| 0.96 | 0.018223 | 0.50482 | 0.425159 | 0 | 0.016896 |
| 0.96 | 0.027594 | 0.755788 | 0.216618 | 0 | 0.07257 |
| 0.96 | 0.003406 | 0.599878 | 0.396716 | 0 | 0.045399 |
| 0.94 | 0.021748 | 0.928259 | 0.049992 | 7.31E-07 | 0.043612 |
| 0.94 | 0.016337 | 0.866699 | 0.111594 | 0.00537 | 0.013295 |
| 0.93 | 0.017962 | 0.804942 | 0.163669 | 0.013427 | 0.006701 |
| 0.93 | 0.037805 | 0.73086 | 0.231335 | 0 | 0.156062 |
| 0.93 | 0.177178 | 0.698147 | 0.059042 | 0.065633 | 0.169988 |
| 0.93 | 0.032386 | 0.701748 | 0.255853 | 0.010013 | 0.027642 |
| 0.92 | 0.027806 | 0.664197 | 0.305735 | 0.002262 | 0.027812 |
| 0.91 | 0.042539 | 0.616348 | 0.341113 | 0 | 0.050441 |
| 0.9 | 0.099347 | 0.900653 | 0 | 0 | 0.147427 |
| 0.9 | 0.032369 | 0.54685 | 0.415187 | 0.005593 | 0.023739 |
| 0.89 | 0.004923 | 0.757164 | 0.144813 | 0.0931 | 0.011441 |
| 0.89 | 0.040323 | 0.76684 | 0.192836 | 0 | 0.016819 |
| 0.88 | 0.059954 | 0.694479 | 0.245567 | 0 | 0.029688 |
| 0.87 | 0.078413 | 0.768356 | 0.153232 | 0 | 0.086105 |
| 0.87 | 0.067705 | 0.60951 | 0.322784 | 0 | 0.031049 |
| 0.87 | 0.040363 | 0.948775 | 0.010863 | 0 | 0.06336 |
| 0.86 | 0.04377 | 0.927657 | 0.028573 | 0 | 0.115429 |
| 0.86 | 0.019281 | 0.635579 | 0.264739 | 0.080401 | 0.007959 |
| 0.85 | 0.108231 | 0.891769 | 0 | 0 | 0.081012 |
| 0.85 | 0.092472 | 0.841024 | 0.066505 | 0 | 0.094696 |
| 0.84 | 0.043952 | 0.822823 | 0.084006 | 0.049219 | 0.044707 |
| 0.84 | 0.050098 | 0.942054 | 0.007554 | 0.000294 | 0.032512 |
| 0.81 | 0.012225 | 0.952622 | 0.035153 | 0 | 0.01266 |
| 0.8 | 0.029145 | 0.731754 | 0.289111 | 0 | 0.037475 |
| 0.8 | 0.077048 | 0.723402 | 0.19955 | 0 | 0.077261 |
| 0.8 | 0.062328 | 0.768301 | 0.146098 | 0.023273 | 0.041255 |
| 0.79 | 0.021546 | 0.785222 | 0.093462 | 0.09977 | 0.001934 |
| 0.78 | 0.038505 | 0.828164 | 0.133331 | 0 | 0.114387 |
| 0.78 | 0.02633 | 0.705616 | 0.268053 | 0 | 3.04E-06 |
| 0.78 | 0.022698 | 0.676984 | 0.300318 | 0 | 0.086161 |
| 0.77 | 0.010139 | 0.929534 | 0.039062 | 0.021265 | 0.02452 |
| 0.76 | 0.020504 | 0.893617 | 0.085879 | 0 | 0.028256 |
| 0.75 | 0.071363 | 0.916463 | 0.012174 | 0 | 0.181614 |
| 0.75 | 0.077374 | 0.85315 | 0.00706 | 0.062416 | 0.027737 |
| 0.75 | 0.032981 | 0.830037 | 0.110184 | 0.026798 | 0.145802 |
| 0.74 | 0.018798 | 0.873342 | 0.096714 | 0.011147 | 0.000615 |
| 0.74 | 0.048003 | 0.729414 | 0.209387 | 0.013197 | 0.033985 |
| 0.73 | 0.012158 | 0.877799 | 0.104953 | 0.00509 | 0.119091 |
| 0.73 | 0.046235 | 0.768727 | 0.185038 | 0 | 0.003722 |
| 0.73 | 0.012941 | 0.611311 | 0.36866 | 0.007087 | 0.050402 |
| 0.73 | 0.028556 | 0.832744 | 0.1387 | 0 | 0.027699 |
| 0.72 | 0.045738 | 0.828349 | 0.125913 | 0 | 0.022493 |
| 0.71 | 0.036539 | 0.951535 | 0.011926 | 0 | 0.025928 |
| 0.71 | 0.074518 | 0.828824 | 0.096658 | 0 | 0.049843 |
| 0.7 | 0.034103 | 0.818442 | 0.147454 | 0 | 0.087954 |
| 0.7 | 0.028501 | 0.93417 | 0.037329 | 0 | 7.53E-06 |
| 0.69 | 0.07399 | 0.807539 | 0.022472 | 0.096 | 0.028334 |
| 0.69 | 0.041867 | 0.696324 | 0.261809 | 0 | 0.009857 |
| 0.69 | 0.143477 | 0.843699 | 0.006261 | 0.006563 | 0.063003 |
| 0.67 | 0.060047 | 0.690705 | 0.249248 | 0 | 0.327264 |
| 0.66 | 0.01543 | 0.653371 | 0.299888 | 0.031312 | 0.052174 |
| 0.66 | 0.076638 | 0.893076 | 0.030286 | 0 | 0.003956 |
| 0.65 | 0.0432 | 0.9568 | 0 | 0 | 0.013619 |
| 0.64 | 0.070103 | 0.80001 | 0.098141 | 0.031745 | 0.019618 |
| 0.64 | 0.037419 | 0.923312 | 0.010583 | 0.028685 | 0.061092 |
| 0.64 | 0.033544 | 0.839022 | 0.127434 | 0 | 0.062529 |
| 0.63 | 0.182413 | 0.806462 | 0.011125 | 0 | 0.037714 |
| 0.62 | 0.042068 | 0.913719 | 0.035636 | 0.008577 | 0.070134 |
| 0.61 | 0.005309 | 0.943367 | 0.02594 | 0.025384 | 0.000813 |
| 0.61 | 0.028701 | 0.880606 | 0.090693 | 0 | 0.1192 |
| 0.6 | 0.019964 | 0.944058 | 0.034331 | 0.001647 | 0.054066 |
| 0.6 | 0.030852 | 0.937414 | 0.022909 | 0.008825 | 0.063511 |
| 0.6 | 0.04777 | 0.769868 | 0.182362 | 0 | 0.025439 |
| 0.6 | 0.046376 | 0.702486 | 0.251138 | 0 | 0.003461 |
| 0.6 | 0.04376 | 0.936753 | 0.019487 | 0 | 0.061764 |
| 0.59 | 0.023832 | 0.952769 | 0.012307 | 0.011092 | 0.083228 |
| 0.58 | 0.026149 | 0.925556 | 0.048294 | 0 | 0.048499 |
| 0.58 | 0.091309 | 0.876741 | 0.03195 | 0 | 0.02242 |
| 0.58 | 0.032002 | 0.781137 | 0.152364 | 0.034498 | 0.000807 |
| 0.57 | 0.050745 | 0.719471 | 0.229783 | 0 | 0.077093 |
| 0.56 | 0.142331 | 0.782279 | 0.058797 | 0.016592 | 0.041235 |
| 0.55 | 0.059471 | 0.75569 | 0.181554 | 0.003285 | 0.032832 |
| 0.55 | 0.032119 | 0.666449 | 0.28536 | 0.016073 | 0.089523 |
| 0.54 | 0.008458 | 0.766388 | 0.13723 | 0.087924 | 0.073515 |
| 0.53 | 0.064139 | 0.813701 | 0.122161 | 0 | 0.111806 |
| 0.53 | 0.005345 | 0.589222 | 0.392616 | 0.012817 | 0.022783 |
| 0.52 | 0.023363 | 0.846999 | 0.129638 | 0 | 0.078641 |
| 0.51 | 0.020372 | 0.836787 | 0.071528 | 0.071313 | 0.01532 |
| 0.51 | 0.033119 | 0.862447 | 0.104434 | 0 | 0.024761 |
| 0.51 | 0.151948 | 0.530499 | 0.120228 | 0.197325 | 0.019356 |
| 0.5 | 0.008343 | 0.991657 | 0 | 0 | 0.039714 |
| 0.49 | 0.026939 | 0.973061 | 0 | 0 | 0.021787 |
| 0.49 | 0.096755 | 0.818844 | 0.082158 | 0.002244 | 0.031972 |
| 0.49 | 0.007157 | 0.760099 | 0.232744 | 0 | 0.138899 |
| 0.48 | 0.027087 | 0.892398 | 0.06958 | 0.010935 | 0.015594 |
| 0.47 | 0.056379 | 0.943621 | 0 | 0 | 0.123161 |
| 0.47 | 0.0599 | 0.70447 | 0.203372 | 0.032257 | 0.035467 |
| 0.47 | 0.018303 | 0.841083 | 0.134267 | 0.006347 | 0.007471 |
| 0.46 | 0.016397 | 0.951369 | 0.032233 | 0 | 0.022951 |
| 0.45 | 0.02416 | 0.973681 | 0.00216 | 0 | 0.016484 |
| 0.45 | 0.279126 | 0.620004 | 0.100869 | 0 | 0.032547 |
| 0.45 | 0.002975 | 0.947564 | 0.037218 | 0.012244 | 0.071134 |
| 0.45 | 0.072476 | 0.909047 | 0.018477 | 0 | 0.060021 |
| 0.41 | 0.056828 | 0.90978 | 0.014798 | 0.018594 | 0.01458 |
| 0.41 | 0.020885 | 0.97362 | 0.005495 | 0 | 0.031392 |
| 0.38 | 0.01264 | 0.937165 | 0.043062 | 0.007133 | 0.185625 |
| 0.38 | 0.122952 | 0.730519 | 0.14653 | 0 | 0.089381 |
| 0.37 | 0.022281 | 0.817366 | 0.144456 | 0.015897 | 0.002968 |
| 0.37 | 0.054189 | 0.877665 | 0.068146 | 0 | 0.141787 |
| 0.33 | 0.061675 | 0.89241 | 0.045559 | 0.000356 | 0.165958 |
| 0.32 | 0.059391 | 0.887374 | 0.052975 | 0.00026 | 0.002722 |
| 0.31 | 0.016053 | 0.877654 | 0.080995 | 0.025299 | 0.022378 |
| 0.3 | 0.031151 | 0.746825 | 0.203347 | 0.018677 | 0.101497 |
| 0.3 | 0.055696 | 0.879582 | 0.060887 | 0.003835 | 0.021538 |
| 0.27 | 0.05676 | 0.713201 | 0.23004 | 0 | 0.025905 |
| 0.27 | 0.011622 | 0.828127 | 0.160251 | 0 | 0.020163 |
| 0.26 | 0.019231 | 0.918595 | 0.062174 | 0 | 0.02441 |
| 0.26 | 0.039841 | 0.816561 | 0.143598 | 0 | 0.101836 |
| 0.25 | 0.014656 | 0.937846 | 0.041141 | 0.006357 | 0.017017 |
| 0.24 | 0.052255 | 0.898211 | 0.046067 | 0.003468 | 0.000502 |
| 0.23 | 0.11188 | 0.782154 | 0.105966 | 0 | 0.001959 |
| 0.23 | 0.036829 | 0.673528 | 0.283352 | 0.006291 | 0.031069 |
| 0.16 | 0.049856 | 0.810036 | 0.140108 | 0 | 0.019612 |
| 0.15 | 0.029173 | 0.847523 | 0.123304 | 0 | 0.02368 |
| 0.15 | 0.08957 | 0.6941 | 0.21633 | 0 | 0.055495 |
| 0.14 | 0.035536 | 0.632685 | 0.323556 | 0.008224 | 0.021802 |
| 0.14 | 0.030356 | 0.662937 | 0.306707 | 0 | 0.021427 |

*European badger*

| Harvest  (ind./100ha) | SETTLEMENTS  (%) | CROPS  (%) | FORESTS  (%) | WATER  (%) | WIND FARM  (%) |
| --- | --- | --- | --- | --- | --- |
| 5.62 | 0.064454 | 0.807537 | 0.118761 | 0.009248 | 0.039809 |
| 4.97 | 0.03398 | 0.965065 | 0.000956 | 0 | 0.457655 |
| 4.97 | 0.041354 | 0.793022 | 0.109663 | 0.055961 | 0.156689 |
| 4.91 | 0.02225 | 0.900537 | 0.067932 | 0.00928 | 0.000577 |
| 4.79 | 0.078715 | 0.778306 | 0.133458 | 0.009521 | 0.022315 |
| 4.63 | 0.017478 | 0.896971 | 0.020908 | 0.064643 | 0.124726 |
| 4.4 | 0.034684 | 0.90422 | 0.061095 | 0 | 0.079926 |
| 4.37 | 0.030872 | 0.686417 | 0.272097 | 0.010614 | 0.010509 |
| 4.36 | 0.181455 | 0.617007 | 0.187585 | 0.013953 | 0.12495 |
| 4.17 | 0.051785 | 0.901516 | 0.046699 | 0 | 0.007171 |
| 4.07 | 0.130716 | 0.674756 | 0.194527 | 0 | 0.138547 |
| 4.01 | 0.001377 | 0.682013 | 0.307943 | 0.008666 | 0.024811 |
| 3.99 | 0.095858 | 0.675914 | 0.223198 | 0.005031 | 0.017825 |
| 3.65 | 0.012366 | 0.881566 | 0.053689 | 0.052379 | 0.104641 |
| 3.62 | 0.130492 | 0.869508 | 0 | 0 | 0.036249 |
| 3.58 | 0.086482 | 0.913518 | 0 | 0 | 0.005667 |
| 3.51 | 0.016337 | 0.866699 | 0.111594 | 0.00537 | 0.013295 |
| 3.26 | 0.030864 | 0.930063 | 0 | 0.039073 | 0.003036 |
| 3.13 | 0.03309 | 0.827144 | 0.139765 | 0 | 0.030663 |
| 3.11 | 0.137764 | 0.692755 | 0.169481 | 0 | 0.01181 |
| 3.07 | 0.021323 | 0.624195 | 0.354482 | 0 | 0.002746 |
| 2.99 | 0.041708 | 0.943745 | 0 | 0.014547 | 0.008971 |
| 2.98 | 0.036323 | 0.95832 | 0.005356 | 0 | 0.348035 |
| 2.98 | 0.050363 | 0.949637 | 0 | 0 | 0.000103 |
| 2.98 | 0.026258 | 0.530627 | 0.436197 | 0.006918 | 0.028396 |
| 2.95 | 0.050548 | 0.895549 | 0.053477 | 0.000426 | 0.040513 |
| 2.9 | 0.041854 | 0.859523 | 0.094217 | 0.004407 | 0.152709 |
| 2.89 | 0.036312 | 0.904341 | 0.024842 | 0.034504 | 0.160208 |
| 2.89 | 0.059836 | 0.773228 | 0.059752 | 0.107184 | 0.135669 |
| 2.88 | 0.037255 | 0.909058 | 0.053687 | 0 | 0.110157 |
| 2.87 | 0.010053 | 0.727504 | 0.207121 | 0.055322 | 0.048494 |
| 2.72 | 0.019528 | 0.767714 | 0.212758 | 0 | 0.030766 |
| 2.7 | 0.030193 | 0.919865 | 0.019158 | 0.030784 | 0.093644 |
| 2.65 | 0.012828 | 0.801466 | 0.169907 | 0.015799 | 0.035618 |
| 2.56 | 0.046888 | 0.885726 | 0.036393 | 0.030993 | 0.012365 |
| 2.51 | 0.046194 | 0.608006 | 0.345294 | 0.000506 | 0.002681 |
| 2.51 | 0.048432 | 0.813801 | 0.137767 | 0 | 0.016043 |
| 2.5 | 0.101064 | 0.634403 | 0.264178 | 0.000356 | 0.002776 |
| 2.5 | 0.015311 | 0.850012 | 0.134677 | 0 | 0.043252 |
| 2.49 | 0.024205 | 0.53313 | 0.202177 | 0.240487 | 0.002058 |
| 2.48 | 0.134499 | 0.702936 | 0.162566 | 0 | 0.002641 |
| 2.47 | 0.041239 | 0.678391 | 0.280371 | 0 | 0.166558 |
| 2.44 | 0.033665 | 0.827557 | 0.095811 | 0.042966 | 0.085835 |
| 2.42 | 0.028434 | 0.931806 | 0.027306 | 0.012453 | 0.127255 |
| 2.41 | 0.009221 | 0.887556 | 0.073236 | 0.029987 | 0.245076 |
| 2.41 | 0.028499 | 0.562559 | 0.360137 | 0.048805 | 0.001166 |
| 2.38 | 0.018482 | 0.571366 | 0.403494 | 0.006658 | 0.08103 |
| 2.37 | 0.077906 | 0.776797 | 0.145296 | 0 | 0.026626 |
| 2.33 | 0.011822 | 0.922221 | 0.0577 | 0.008256 | 0.027789 |
| 2.33 | 0.031482 | 0.918601 | 0 | 0.049917 | 0.016405 |
| 2.26 | 0.027274 | 0.638686 | 0.329299 | 0.004741 | 0.087119 |
| 2.24 | 0.027304 | 0.917848 | 0.04837 | 0.006477 | 0.349861 |
| 2.24 | 0.139056 | 0.729618 | 0.11691 | 0.014416 | 2.54E-05 |
| 2.21 | 0.110639 | 0.740989 | 0.108626 | 0.039746 | 0.079146 |
| 2.19 | 0.011426 | 0.976589 | 0.011985 | 0 | 0.007056 |
| 2.19 | 0.068853 | 0.901758 | 0.029389 | 0 | 0.153666 |
| 2.17 | 0.018063 | 0.899386 | 0.044394 | 0.038157 | 0.085235 |
| 2.15 | 0.022756 | 0.827933 | 0.149312 | 0 | 0.036017 |
| 2.14 | 0.058012 | 0.561444 | 0.380544 | 0 | 0.028502 |
| 2.12 | 0.034088 | 0.734203 | 0.231709 | 0 | 0.003313 |
| 2.09 | 0.031613 | 0.961818 | 0.003158 | 0.003411 | 0.046069 |
| 2.09 | 0.070047 | 0.841396 | 0.083412 | 0.005146 | 0.038956 |
| 2.05 | 0.092208 | 0.70089 | 0.190913 | 0.015988 | 0.053543 |
| 2.03 | 0.048241 | 0.890315 | 0.061444 | 0 | 0.004877 |
| 2.03 | 0.014978 | 0.876519 | 0.099945 | 0.008559 | 0.01608 |
| 2 | 0.049178 | 0.932341 | 0.018482 | 0 | 0.034648 |
| 2 | 0.063452 | 0.859274 | 0.077275 | 0 | 0.167713 |
| 1.95 | 0.050611 | 0.799196 | 0.130295 | 0.019897 | 0.045145 |
| 1.94 | 0.048955 | 0.647511 | 0.303534 | 0 | 0.045488 |
| 1.93 | 0.03505 | 0.940453 | 0 | 0.024497 | 0.000582 |
| 1.93 | 0.04551 | 0.731711 | 0.222779 | 0 | 0.007162 |
| 1.92 | 0.124811 | 0.814342 | 0.060847 | 0 | 0.105066 |
| 1.91 | 0.096072 | 0.863331 | 0.040596 | 0 | 0.08757 |
| 1.91 | 0.01712 | 0.800569 | 0.132823 | 0.049489 | 0.06699 |
| 1.91 | 0.03957 | 0.723527 | 0.232789 | 0.004115 | 0.000244 |
| 1.89 | 0.035856 | 0.89092 | 0.073224 | 0 | 0.152419 |
| 1.88 | 0.004494 | 0.931113 | 0.025152 | 0.03924 | 0.009948 |
| 1.88 | 0.027328 | 0.959282 | 0.005139 | 0.008252 | 0.023328 |
| 1.87 | 0.073048 | 0.7756 | 0.151352 | 0 | 0.061654 |
| 1.86 | 0.032386 | 0.701748 | 0.255853 | 0.010013 | 0.027642 |
| 1.85 | 0.049931 | 0.628573 | 0.321228 | 0.000269 | 0.036331 |
| 1.83 | 0.04838 | 0.768229 | 0.137646 | 0.045745 | 0.000874 |
| 1.83 | 0.03717 | 0.760903 | 0.201927 | 0 | 0.006919 |
| 1.83 | 0.054568 | 0.700053 | 0.236636 | 0.008742 | 0.051978 |
| 1.83 | 0.049055 | 0.900592 | 0.050353 | 0 | 0.099 |
| 1.8 | 0.039445 | 0.880548 | 0.078509 | 0.001499 | 0.00146 |
| 1.8 | 0.222442 | 0.74335 | 0.034208 | 0 | 0.006338 |
| 1.78 | 0.045885 | 0.95365 | 0.000465 | 0 | 0.003323 |
| 1.78 | 0.137039 | 0.844241 | 0.01872 | 0 | 0.054031 |
| 1.78 | 0.054088 | 0.749397 | 0.163075 | 0.03344 | 0.116868 |
| 1.77 | 0.052284 | 0.743996 | 0.193735 | 0.009985 | 0.045387 |
| 1.72 | 0.069289 | 0.841813 | 0.088899 | 0 | 0.074837 |
| 1.71 | 0.09225 | 0.835858 | 0.071892 | 0 | 0.064066 |
| 1.71 | 0.21499 | 0.702711 | 0.080053 | 0.002246 | 0.007246 |
| 1.7 | 0.058187 | 0.528333 | 0.413479 | 0 | 0.002676 |
| 1.7 | 0.025165 | 0.709589 | 0.265246 | 0 | 0.340679 |
| 1.69 | 0.027717 | 0.596513 | 0.370443 | 0.005327 | 0.035165 |
| 1.69 | 0.025413 | 0.787728 | 0.186858 | 0 | 0.021162 |
| 1.68 | 0.05913 | 0.575212 | 0.365601 | 5.72E-05 | 0.03847 |
| 1.65 | 0.080504 | 0.831155 | 0.005685 | 0.082657 | 0.041065 |
| 1.65 | 0.015465 | 0.974209 | 0.010326 | 0 | 0.001597 |
| 1.64 | 0.023232 | 0.748449 | 0.204923 | 0.023397 | 0.029774 |
| 1.64 | 0.039363 | 0.856503 | 0.095384 | 0.00875 | 0.023128 |
| 1.63 | 0.045934 | 0.924793 | 0.02209 | 0.007184 | 0.19778 |
| 1.63 | 0.139615 | 0.679458 | 0.17389 | 0.007037 | 0.000136 |
| 1.63 | 0.080689 | 0.873851 | 0.041035 | 0.004425 | 0.180591 |
| 1.63 | 0.046144 | 0.680903 | 0.254461 | 0.018493 | 0.000879 |
| 1.63 | 0.111642 | 0.775675 | 0.112682 | 0 | 0.047805 |
| 1.59 | 0.029145 | 0.731754 | 0.289111 | 0 | 0.037475 |
| 1.59 | 0.055185 | 0.873829 | 0.070986 | 0 | 0.060068 |
| 1.59 | 0.025498 | 0.828399 | 0.114062 | 0.032041 | 0.078826 |
| 1.59 | 0.036625 | 0.770464 | 0.167258 | 0.025652 | 0.178928 |
| 1.58 | 0.024747 | 0.964457 | 0.010796 | 0 | 0.256071 |
| 1.57 | 0.036113 | 0.84386 | 0.028627 | 0.0914 | 0.01802 |
| 1.57 | 0.18621 | 0.781401 | 0.032389 | 0 | 0.147147 |
| 1.57 | 0.157547 | 0.729757 | 0.10907 | 0.003625 | 0.027969 |
| 1.56 | 0.038505 | 0.828164 | 0.133331 | 0 | 0.114387 |
| 1.54 | 0.010139 | 0.929534 | 0.039062 | 0.021265 | 0.02452 |
| 1.53 | 0.095985 | 0.700639 | 0.198975 | 0.0044 | 0.02055 |
| 1.53 | 0.040076 | 0.833117 | 0.087333 | 0.039474 | 0.241711 |
| 1.52 | 0.051852 | 0.630668 | 0.304929 | 0.01255 | 0.049537 |
| 1.52 | 0.065776 | 0.916671 | 0.017553 | 0 | 0.021232 |
| 1.51 | 0.01264 | 0.937165 | 0.043062 | 0.007133 | 0.185625 |
| 1.5 | 0.032369 | 0.54685 | 0.415187 | 0.005593 | 0.023739 |
| 1.5 | 0.008526 | 0.940563 | 0.050911 | 0 | 0.124726 |
| 1.5 | 0.033849 | 0.683169 | 0.28066 | 0.002321 | 0.049347 |
| 1.5 | 0.034773 | 0.709889 | 0.247028 | 0.008309 | 0.059819 |
| 1.48 | 0.063263 | 0.772515 | 0.155506 | 0.008716 | 0.17232 |
| 1.48 | 0.053764 | 0.773062 | 0.173173 | 0 | 0.151376 |
| 1.47 | 0.074807 | 0.798245 | 0.126948 | 0 | 0.052297 |
| 1.46 | 0.025046 | 0.688707 | 0.286247 | 0 | 0.026522 |
| 1.45 | 0.029447 | 0.903798 | 0.066755 | 0 | 0.23376 |
| 1.45 | 0.032845 | 0.784505 | 0.155122 | 0.027527 | 0.09242 |
| 1.44 | 0.035595 | 0.757742 | 0.206663 | 0 | 0.026593 |
| 1.43 | 0.039287 | 0.659343 | 0.239964 | 0.061407 | 0.021402 |
| 1.43 | 0.216664 | 0.779225 | 0.004111 | 0 | 0.294511 |
| 1.41 | 0.01082 | 0.979523 | 0 | 0.009656 | 0.210532 |
| 1.41 | 0.043831 | 0.54447 | 0.298957 | 0.112741 | 0.002629 |
| 1.37 | 0.119335 | 0.541145 | 0.336507 | 0.003013 | 0.018434 |
| 1.37 | 0.029603 | 0.751571 | 0.218827 | 0 | 0.079736 |
| 1.36 | 0.007743 | 0.882418 | 0.104562 | 0.005277 | 0.030295 |
| 1.36 | 0.05676 | 0.713201 | 0.23004 | 0 | 0.025905 |
| 1.35 | 0.039863 | 0.711922 | 0.248216 | 0 | 0.020078 |
| 1.35 | 0.003095 | 0.950925 | 0.015182 | 0.030799 | 0.103433 |
| 1.34 | 0.065773 | 0.829725 | 0.103836 | 0.000666 | 0.181817 |
| 1.34 | 0.016972 | 0.707628 | 0.225538 | 0.049862 | 0.111867 |
| 1.34 | 0.203607 | 0.526209 | 0.270184 | 0 | 0.050672 |
| 1.33 | 0.022977 | 0.95925 | 0.006649 | 0.011124 | 0.025333 |
| 1.33 | 0.014883 | 0.952852 | 0.026053 | 0.006211 | 0.039717 |
| 1.32 | 0.017027 | 0.946215 | 0.036758 | 0 | 0.005504 |
| 1.32 | 0.11287 | 0.864337 | 0.022793 | 0 | 0.094936 |
| 1.32 | 0.01259 | 0.695308 | 0.274294 | 0.017807 | 0.002173 |
| 1.32 | 0.021317 | 0.804667 | 0.174016 | 0 | 0.278978 |
| 1.31 | 0.097723 | 0.899411 | 0.002866 | 0 | 0.018141 |
| 1.31 | 0.074414 | 0.869734 | 0.019153 | 0.036699 | 0.003266 |
| 1.3 | 0.009555 | 0.979002 | 0.000198 | 0.011245 | 0.076538 |
| 1.3 | 0.069142 | 0.772286 | 0.14675 | 0.011822 | 0.039618 |
| 1.3 | 0.043339 | 0.663185 | 0.278789 | 0.014687 | 0.102922 |
| 1.28 | 0.042539 | 0.616348 | 0.341113 | 0 | 0.050441 |
| 1.28 | 0.009447 | 0.761519 | 0.21221 | 0.016824 | 0.01016 |
| 1.27 | 0.059469 | 0.940531 | 0 | 0 | 0.036402 |
| 1.27 | 0.059243 | 0.805019 | 0.135738 | 0 | 0.094313 |
| 1.27 | 0.030802 | 0.910639 | 0.052474 | 0.006085 | 0.34264 |
| 1.26 | 0.110983 | 0.870325 | 0.018692 | 0 | 0.141334 |
| 1.26 | 0.029013 | 0.883584 | 0.082962 | 0.004441 | 0.000183 |
| 1.25 | 0.020569 | 0.771368 | 0.155748 | 0.052315 | 0.015713 |
| 1.25 | 0.041876 | 0.634991 | 0.323132 | 0 | 0.019803 |
| 1.25 | 0.035743 | 0.671162 | 0.284171 | 0.008924 | 0.015164 |
| 1.25 | 0.077148 | 0.72609 | 0.196763 | 0 | 0.022359 |
| 1.25 | 0.085555 | 0.904837 | 0.009608 | 0 | 0.034194 |
| 1.24 | 0.030564 | 0.913953 | 0.055484 | 0 | 0.087083 |
| 1.23 | 0.01756 | 0.936756 | 0.045684 | 0 | 0.587545 |
| 1.22 | 0.029573 | 0.84438 | 0.121215 | 0.004832 | 0.031662 |
| 1.21 | 0.036485 | 0.81515 | 0.148365 | 0 | 0.19155 |
| 1.21 | 0.074589 | 0.640797 | 0.26404 | 0.020574 | 0.021459 |
| 1.21 | 0.007404 | 0.887462 | 0.105134 | 0 | 0.179264 |
| 1.2 | 0.058021 | 0.662699 | 0.258411 | 0.020869 | 0.038288 |
| 1.19 | 0.046998 | 0.826689 | 0.126312 | 0 | 0.087959 |
| 1.19 | 0.053113 | 0.649051 | 0.261498 | 0.036338 | 0.042373 |
| 1.18 | 0.141596 | 0.638012 | 0.156134 | 0.064258 | 0.108228 |
| 1.18 | 0.015126 | 0.759174 | 0.204304 | 0.021397 | 0.097869 |
| 1.18 | 0.046884 | 0.923363 | 0.029753 | 0 | 0.00521 |
| 1.17 | 0.07946 | 0.663096 | 0.257444 | 0 | 0.119837 |
| 1.17 | 0.019597 | 0.789053 | 0.19135 | 0 | 0.007049 |
| 1.17 | 0.105751 | 0.72069 | 0.166077 | 0.007481 | 0.034231 |
| 1.16 | 0.078413 | 0.768356 | 0.153232 | 0 | 0.086105 |
| 1.16 | 0.064382 | 0.647883 | 0.274131 | 0.013604 | 0.054466 |
| 1.16 | 0.032721 | 0.728697 | 0.238522 | 5.97E-05 | 0.018052 |
| 1.16 | 0.153329 | 0.620561 | 0.18755 | 0.031629 | 0.091145 |
| 1.15 | 0.072737 | 0.703643 | 0.172948 | 0.050672 | 0.003121 |
| 1.15 | 0.07399 | 0.807539 | 0.022472 | 0.096 | 0.028334 |
| 1.14 | 0.039 | 0.770268 | 0.167469 | 0.023263 | 0.142616 |
| 1.14 | 0.025069 | 0.954991 | 0.01994 | 0 | 0.029211 |
| 1.14 | 0.072977 | 0.856731 | 0.070292 | 0 | 0.074098 |
| 1.13 | 0.008032 | 0.962155 | 0.004333 | 0.02548 | 0.026076 |
| 1.12 | 0.017755 | 0.694353 | 0.28756 | 0.000331 | 0.007311 |
| 1.12 | 0.009634 | 0.907754 | 0.082612 | 0 | 0.016301 |
| 1.11 | 0.12475 | 0.506296 | 0.368954 | 0 | 0.018843 |
| 1.1 | 0.134972 | 0.724281 | 0.120111 | 0.020636 | 0.008062 |
| 1.1 | 0.079122 | 0.728452 | 0.189573 | 0.002853 | 0.013886 |
| 1.09 | 0.012158 | 0.877799 | 0.104953 | 0.00509 | 0.119091 |
| 1.09 | 0.028556 | 0.832744 | 0.1387 | 0 | 0.027699 |
| 1.08 | 0.027512 | 0.92664 | 0.024607 | 0.02124 | 0.166807 |
| 1.08 | 0.065527 | 0.728519 | 0.126969 | 0.078986 | 0.047136 |
| 1.07 | 0.029263 | 0.64246 | 0.263832 | 0.064444 | 0.068015 |
| 1.07 | 0.043956 | 0.854424 | 0.090371 | 0.011248 | 0.042874 |
| 1.06 | 0.064139 | 0.813701 | 0.122161 | 0 | 0.111806 |
| 1.06 | 0.045044 | 0.886899 | 0.026934 | 0.041122 | 0.00106 |
| 1.06 | 0.071109 | 0.886488 | 0.042403 | 0 | 0.027537 |
| 1.06 | 0.102823 | 0.881072 | 0.016105 | 0 | 0.048898 |
| 1.05 | 0.033275 | 0.900866 | 0.060343 | 0.005515 | 0.091831 |
| 1.05 | 0.005345 | 0.589222 | 0.392616 | 0.012817 | 0.022783 |
| 1.05 | 0.025726 | 0.568486 | 0.358865 | 0.046922 | 0.138587 |
| 1.04 | 0.098285 | 0.880774 | 0.020941 | 0 | 0.173938 |
| 1.04 | 0.01512 | 0.858559 | 0.07309 | 0.053231 | 0.037677 |
| 1.04 | 0.077189 | 0.793972 | 0.122556 | 0.006282 | 0.067732 |
| 1.04 | 0.077519 | 0.685448 | 0.237032 | 0 | 0.018202 |
| 1.03 | 0.046989 | 0.861338 | 0.091672 | 0 | 0.288311 |
| 1.03 | 0.041867 | 0.696324 | 0.261809 | 0 | 0.009857 |
| 1.03 | 0.039215 | 0.907213 | 0.053573 | 0 | 0.070329 |
| 1.03 | 0.072839 | 0.741172 | 0.185989 | 0 | 0.02913 |
| 1.02 | 0.032947 | 0.773658 | 0.145192 | 0.048202 | 0.049896 |
| 1.02 | 0.039022 | 0.789893 | 0.171085 | 0 | 0.011916 |
| 1.01 | 0.020885 | 0.97362 | 0.005495 | 0 | 0.031392 |
| 1.01 | 0.02289 | 0.955775 | 0.021335 | 0 | 0.120855 |
| 1.01 | 0.142331 | 0.782279 | 0.058797 | 0.016592 | 0.041235 |
| 1.01 | 0.072101 | 0.86385 | 0.054567 | 0.009482 | 0.064817 |
| 1.01 | 0.040823 | 0.959177 | 0 | 0 | 0.004214 |
| 1.01 | 0.029025 | 0.753063 | 0.182242 | 0.03567 | 0.000693 |
| 1.01 | 0.096224 | 0.839476 | 0.0643 | 0 | 0.055361 |
| 1.01 | 0.061571 | 0.728748 | 0.209681 | 0 | 0.041562 |
| 1 | 0.102583 | 0.897417 | 0 | 0 | 0.045341 |
| 1 | 0.013851 | 0.854947 | 0.094772 | 0.036431 | 0.005141 |
| 1 | 0.049629 | 0.944396 | 0.005975 | 0 | 0.171729 |
| 0.98 | 0.056263 | 0.943737 | 0 | 0 | 0.41748 |
| 0.97 | 0.042169 | 0.597119 | 0.360712 | 0 | 0.036652 |
| 0.97 | 0.030907 | 0.705282 | 0.26381 | 0 | 0.210419 |
| 0.96 | 0.018223 | 0.50482 | 0.425159 | 0 | 0.016896 |
| 0.96 | 0.046376 | 0.702486 | 0.251138 | 0 | 0.003461 |
| 0.96 | 0.012081 | 0.564216 | 0.390193 | 0.03351 | 0.035279 |
| 0.95 | 0.021237 | 0.959433 | 0.019331 | 0 | 0.256702 |
| 0.94 | 0.101092 | 0.659995 | 0.223647 | 0.015266 | 0.004566 |
| 0.93 | 0.017962 | 0.804942 | 0.163669 | 0.013427 | 0.006701 |
| 0.93 | 0.041847 | 0.933975 | 0 | 0.024178 | 0.027727 |
| 0.92 | 0.069296 | 0.774471 | 0.070706 | 0.085528 | 0.031938 |
| 0.92 | 0.04601 | 0.785236 | 0.14245 | 0.026304 | 0.131842 |
| 0.91 | 0.011447 | 0.645638 | 0.323308 | 0.019607 | 0.001809 |
| 0.91 | 0.051831 | 0.828658 | 0.11951 | 0 | 0.107904 |
| 0.9 | 0.019964 | 0.944058 | 0.034331 | 0.001647 | 0.054066 |
| 0.9 | 0.060047 | 0.690705 | 0.249248 | 0 | 0.327264 |
| 0.89 | 0.004923 | 0.757164 | 0.144813 | 0.0931 | 0.011441 |
| 0.89 | 0.040323 | 0.76684 | 0.192836 | 0 | 0.016819 |
| 0.89 | 0.037227 | 0.774049 | 0.188724 | 0 | 0.136711 |
| 0.88 | 0.083189 | 0.898119 | 0.012762 | 0.00593 | 0.16835 |
| 0.88 | 0.098187 | 0.786894 | 0.114919 | 0 | 0.033695 |
| 0.87 | 0.067705 | 0.60951 | 0.322784 | 0 | 0.031049 |
| 0.87 | 0.073033 | 0.813903 | 0.097747 | 0.015317 | 0.19531 |
| 0.87 | 0.026149 | 0.925556 | 0.048294 | 0 | 0.048499 |
| 0.87 | 0.040363 | 0.948775 | 0.010863 | 0 | 0.06336 |
| 0.86 | 0.049996 | 0.803418 | 0.146586 | 0 | 0.137218 |
| 0.85 | 0.092472 | 0.841024 | 0.066505 | 0 | 0.094696 |
| 0.84 | 0.007534 | 0.991543 | 0 | 0.000922 | 0.020689 |
| 0.84 | 0.043952 | 0.822823 | 0.084006 | 0.049219 | 0.044707 |
| 0.83 | 0.08025 | 0.857246 | 0.062497 | 7.34E-06 | 0.108955 |
| 0.83 | 0.032421 | 0.811665 | 0.155915 | 0 | 0.079009 |
| 0.83 | 0.041745 | 0.712863 | 0.243395 | 0.001997 | 0.02306 |
| 0.83 | 0.025748 | 0.720481 | 0.253771 | 0 | 0.036214 |
| 0.83 | 0.010983 | 0.903515 | 0.085502 | 0 | 0.036375 |
| 0.83 | 0.076638 | 0.893076 | 0.030286 | 0 | 0.003956 |
| 0.83 | 0.066768 | 0.576755 | 0.179528 | 0.176949 | 0.046495 |
| 0.83 | 0.04557 | 0.58709 | 0.36734 | 0 | 0.070527 |
| 0.82 | 0.081238 | 0.718524 | 0.200238 | 0 | 0.013759 |
| 0.82 | 0.029683 | 0.738291 | 0.186006 | 0.046019 | 0.017025 |
| 0.81 | 0.058289 | 0.8764 | 0.065311 | 0 | 0.202343 |
| 0.81 | 0.028579 | 0.779842 | 0.184295 | 0.007283 | 0.00428 |
| 0.81 | 0.035435 | 0.823689 | 0.105185 | 0.035691 | 0.047584 |
| 0.8 | 0.016859 | 0.887013 | 0.096128 | 0 | 0.07925 |
| 0.8 | 0.059068 | 0.905269 | 0.035663 | 0 | 0.061176 |
| 0.79 | 0.03372 | 0.954883 | 0.011397 | 0 | 0.059214 |
| 0.78 | 0.02633 | 0.705616 | 0.268053 | 0 | 3.04E-06 |
| 0.78 | 0.028722 | 0.608216 | 0.319598 | 0.043464 | 0.002594 |
| 0.78 | 0.013546 | 0.804015 | 0.125482 | 0.056958 | 0.043361 |
| 0.78 | 0.042068 | 0.913719 | 0.035636 | 0.008577 | 0.070134 |
| 0.78 | 0.022698 | 0.676984 | 0.300318 | 0 | 0.086161 |
| 0.77 | 0.06005 | 0.93995 | 0 | 0 | 0.023202 |
| 0.77 | 0.053881 | 0.915746 | 0.006846 | 0.023526 | 0.034166 |
| 0.77 | 0.100035 | 0.878576 | 0 | 0.021389 | 0.004507 |
| 0.77 | 0.003406 | 0.599878 | 0.396716 | 0 | 0.045399 |
| 0.77 | 0.04306 | 0.876667 | 0.080273 | 0 | 0.034083 |
| 0.76 | 0.014212 | 0.940359 | 0.02911 | 0.01632 | 0.085242 |
| 0.75 | 0.020053 | 0.595764 | 0.384183 | 0 | 0.022035 |
| 0.75 | 0.279126 | 0.620004 | 0.100869 | 0 | 0.032547 |
| 0.75 | 0.08742 | 0.91258 | 0 | 0 | 0.040064 |
| 0.75 | 0.043989 | 0.711189 | 0.244822 | 0 | 0.171178 |
| 0.75 | 0.077374 | 0.85315 | 0.00706 | 0.062416 | 0.027737 |
| 0.75 | 0.039739 | 0.691329 | 0.268932 | 0 | 0.195716 |
| 0.74 | 0.054189 | 0.877665 | 0.068146 | 0 | 0.141787 |
| 0.74 | 0.048003 | 0.729414 | 0.209387 | 0.013197 | 0.033985 |
| 0.74 | 0.034493 | 0.812457 | 0.153051 | 0 | 0.073334 |
| 0.74 | 0.023883 | 0.761086 | 0.215031 | 0 | 0.033082 |
| 0.74 | 0.023913 | 0.904383 | 0.050222 | 0.021483 | 0.365279 |
| 0.74 | 0.032367 | 0.938775 | 0.019579 | 0.009278 | 0.008665 |
| 0.73 | 0.046235 | 0.768727 | 0.185038 | 0 | 0.003722 |
| 0.73 | 0.012941 | 0.611311 | 0.36866 | 0.007087 | 0.050402 |
| 0.72 | 0.086936 | 0.888209 | 0.024855 | 0 | 0.080122 |
| 0.72 | 0.026162 | 0.710156 | 0.240745 | 0.022937 | 0.031545 |
| 0.71 | 0.044407 | 0.778417 | 0.176709 | 0.000467 | 0.032712 |
| 0.71 | 0.036539 | 0.951535 | 0.011926 | 0 | 0.025928 |
| 0.71 | 0.043407 | 0.941455 | 0.015138 | 0 | 0.002294 |
| 0.71 | 0.112847 | 0.540884 | 0.2825 | 0.063769 | 0.234756 |
| 0.7 | 0.077451 | 0.901627 | 0.020923 | 0 | 0.037246 |
| 0.7 | 0.108022 | 0.691244 | 0.180332 | 0.020402 | 0.004315 |
| 0.7 | 0.028501 | 0.93417 | 0.037329 | 0 | 7.53E-06 |
| 0.7 | 0.018303 | 0.841083 | 0.134267 | 0.006347 | 0.007471 |
| 0.69 | 0.060899 | 0.775398 | 0.163702 | 0 | 0.022238 |
| 0.69 | 0.143477 | 0.843699 | 0.006261 | 0.006563 | 0.063003 |
| 0.69 | 0.035474 | 0.794716 | 0.16981 | 0 | 0.222865 |
| 0.68 | 0.066093 | 0.856123 | 0.077784 | 0 | 0.032206 |
| 0.68 | 0.059338 | 0.879605 | 0.056493 | 0 | 0.222732 |
| 0.68 | 0.041801 | 0.944368 | 0 | 0.013831 | 0.010372 |
| 0.68 | 0.029925 | 0.788213 | 0.181862 | 0 | 0.020835 |
| 0.67 | 0.028831 | 0.777557 | 0.193612 | 0 | 0.01115 |
| 0.67 | 0.033119 | 0.862447 | 0.104434 | 0 | 0.024761 |
| 0.67 | 0.028999 | 0.817861 | 0.15314 | 0 | 0.078066 |
| 0.67 | 0.012289 | 0.967888 | 0.019822 | 0 | 0.169411 |
| 0.66 | 0.060716 | 0.682299 | 0.256985 | 0 | 0.053094 |
| 0.66 | 0.037281 | 0.903436 | 0.042729 | 0.016554 | 0.047312 |
| 0.66 | 0.06371 | 0.687375 | 0.248915 | 0 | 0.024296 |
| 0.66 | 0.04474 | 0.643511 | 0.311749 | 0 | 0.033318 |
| 0.66 | 0.059954 | 0.694479 | 0.245567 | 0 | 0.029688 |
| 0.65 | 0.096755 | 0.818844 | 0.082158 | 0.002244 | 0.031972 |
| 0.65 | 0.019281 | 0.635579 | 0.264739 | 0.080401 | 0.007959 |
| 0.65 | 0.02159 | 0.743128 | 0.235281 | 0 | 0.058284 |
| 0.65 | 0.039787 | 0.928108 | 0.016795 | 0.015311 | 0.04215 |
| 0.64 | 0.021306 | 0.96547 | 0.000434 | 0.01279 | 0.093825 |
| 0.64 | 0.070103 | 0.80001 | 0.098141 | 0.031745 | 0.019618 |
| 0.64 | 0.052039 | 0.626445 | 0.321516 | 0 | 0.038994 |
| 0.64 | 0.006401 | 0.762863 | 0.215353 | 0.015384 | 0.120429 |
| 0.64 | 0.033544 | 0.839022 | 0.127434 | 0 | 0.062529 |
| 0.63 | 0.021748 | 0.928259 | 0.049992 | 7.31E-07 | 0.043612 |
| 0.62 | 0.072848 | 0.708754 | 0.212019 | 0.006379 | 0.2346 |
| 0.61 | 0.005309 | 0.943367 | 0.02594 | 0.025384 | 0.000813 |
| 0.61 | 0.04742 | 0.747043 | 0.205537 | 0 | 0.027979 |
| 0.61 | 0.065419 | 0.70728 | 0.227301 | 0 | 0.288139 |
| 0.61 | 0.03865 | 0.910378 | 0.050972 | 0 | 0.003528 |
| 0.61 | 0.103313 | 0.732831 | 0.163856 | 0 | 0.045637 |
| 0.61 | 0.007157 | 0.760099 | 0.232744 | 0 | 0.138899 |
| 0.61 | 0.028701 | 0.880606 | 0.090693 | 0 | 0.1192 |
| 0.6 | 0.030852 | 0.937414 | 0.022909 | 0.008825 | 0.063511 |
| 0.6 | 0.021006 | 0.91466 | 0.064334 | 0 | 0.031401 |
| 0.6 | 0.071363 | 0.916463 | 0.012174 | 0 | 0.181614 |
| 0.6 | 0.099347 | 0.900653 | 0 | 0 | 0.147427 |
| 0.6 | 0.04376 | 0.936753 | 0.019487 | 0 | 0.061764 |
| 0.59 | 0.031151 | 0.746825 | 0.203347 | 0.018677 | 0.101497 |
| 0.59 | 0.042971 | 0.949884 | 0.007145 | 0 | 0.291543 |
| 0.59 | 0.053152 | 0.914463 | 0.025729 | 0.006655 | 0.029585 |
| 0.59 | 0.023832 | 0.952769 | 0.012307 | 0.011092 | 0.083228 |
| 0.57 | 0.017507 | 0.974532 | 0.00609 | 0.001872 | 0.18464 |
| 0.57 | 0.044084 | 0.893109 | 0.062806 | 0 | 0.010772 |
| 0.57 | 0.030356 | 0.662937 | 0.306707 | 0 | 0.021427 |
| 0.56 | 0.061675 | 0.89241 | 0.045559 | 0.000356 | 0.165958 |
| 0.56 | 0.029175 | 0.877794 | 0.093031 | 0 | 0.015471 |
| 0.56 | 0 | 0.891838 | 0.108162 | 0 | 0.002346 |
| 0.55 | 0.056828 | 0.90978 | 0.014798 | 0.018594 | 0.01458 |
| 0.55 | 0.059471 | 0.75569 | 0.181554 | 0.003285 | 0.032832 |
| 0.55 | 0.041595 | 0.871269 | 0.087137 | 0 | 0.085193 |
| 0.54 | 0.02551 | 0.914172 | 0.045998 | 0.01432 | 0.02754 |
| 0.54 | 0.008458 | 0.766388 | 0.13723 | 0.087924 | 0.073515 |
| 0.54 | 0.034667 | 0.836308 | 0.12772 | 0.001305 | 0.026518 |
| 0.53 | 0.039841 | 0.816561 | 0.143598 | 0 | 0.101836 |
| 0.53 | 0.067538 | 0.901478 | 0.024953 | 0.00603 | 0.018914 |
| 0.52 | 0.010679 | 0.952 | 0.001661 | 0.035661 | 0.004639 |
| 0.52 | 0.168176 | 0.81787 | 0.013954 | 0 | 0.049296 |
| 0.52 | 0.023363 | 0.846999 | 0.129638 | 0 | 0.078641 |
| 0.52 | 0.049416 | 0.637355 | 0.298827 | 0.014403 | 0.051553 |
| 0.52 | 0.022113 | 0.740767 | 0.237121 | 0 | 0.271627 |
| 0.51 | 0.180694 | 0.813429 | 0 | 0.005877 | 0.020059 |
| 0.51 | 0.151948 | 0.530499 | 0.120228 | 0.197325 | 0.019356 |
| 0.5 | 0.008343 | 0.991657 | 0 | 0 | 0.039714 |
| 0.5 | 0.02837 | 0.674304 | 0.282227 | 0.015099 | 0.162311 |
| 0.5 | 0.0274 | 0.680913 | 0.280471 | 0.011216 | 0.088547 |
| 0.5 | 0.032981 | 0.830037 | 0.110184 | 0.026798 | 0.145802 |
| 0.49 | 0.167614 | 0.459503 | 0.216179 | 0.156704 | 0.034594 |
| 0.49 | 0.01216 | 0.758907 | 0.228932 | 0 | 0.023113 |
| 0.49 | 0.026939 | 0.973061 | 0 | 0 | 0.021787 |
| 0.49 | 0.038 | 0.931754 | 0 | 0.030246 | 7.47E-05 |
| 0.49 | 0.067136 | 0.715851 | 0.217013 | 0 | 0.03475 |
| 0.48 | 0.027087 | 0.892398 | 0.06958 | 0.010935 | 0.015594 |
| 0.48 | 0.033553 | 0.805444 | 0.155166 | 0.005836 | 0.089808 |
| 0.47 | 0.030385 | 0.902018 | 0.026566 | 0.041031 | 0.017606 |
| 0.47 | 0.056379 | 0.943621 | 0 | 0 | 0.123161 |
| 0.47 | 0.0599 | 0.70447 | 0.203372 | 0.032257 | 0.035467 |
| 0.47 | 0.074518 | 0.828824 | 0.096658 | 0 | 0.049843 |
| 0.47 | 0.032579 | 0.759046 | 0.200145 | 0.008229 | 0.014697 |
| 0.46 | 0.016397 | 0.951369 | 0.032233 | 0 | 0.022951 |
| 0.46 | 0.037805 | 0.73086 | 0.231335 | 0 | 0.156062 |
| 0.46 | 0.068708 | 0.849552 | 0.042768 | 0.038973 | 0.074069 |
| 0.46 | 0.027806 | 0.664197 | 0.305735 | 0.002262 | 0.027812 |
| 0.45 | 0.002975 | 0.947564 | 0.037218 | 0.012244 | 0.071134 |
| 0.45 | 0.108214 | 0.736279 | 0.126343 | 0.029164 | 0.000839 |
| 0.45 | 0.031001 | 0.661591 | 0.277932 | 0.029476 | 0.000647 |
| 0.44 | 0.08957 | 0.6941 | 0.21633 | 0 | 0.055495 |
| 0.44 | 0.032047 | 0.620483 | 0.34747 | 0 | 0.032438 |
| 0.44 | 0.026963 | 0.570467 | 0.183307 | 0.219263 | 0.0174 |
| 0.44 | 0.01543 | 0.653371 | 0.299888 | 0.031312 | 0.052174 |
| 0.44 | 0.142246 | 0.407022 | 0.377298 | 0.073434 | 0.089493 |
| 0.44 | 0.021547 | 0.897054 | 0.081399 | 0 | 0.003304 |
| 0.43 | 0.050745 | 0.719471 | 0.229783 | 0 | 0.077093 |
| 0.43 | 0.037419 | 0.923312 | 0.010583 | 0.028685 | 0.061092 |
| 0.43 | 0.045738 | 0.828349 | 0.125913 | 0 | 0.022493 |
| 0.42 | 0.108231 | 0.891769 | 0 | 0 | 0.081012 |
| 0.42 | 0.050098 | 0.942054 | 0.007554 | 0.000294 | 0.032512 |
| 0.41 | 0.012878 | 0.947298 | 0.03675 | 0.003074 | 0.028568 |
| 0.41 | 0.055552 | 0.69975 | 0.239609 | 0.005088 | 0.003034 |
| 0.41 | 0.011622 | 0.828127 | 0.160251 | 0 | 0.020163 |
| 0.41 | 0.012225 | 0.952622 | 0.035153 | 0 | 0.01266 |
| 0.41 | 0.072147 | 0.370505 | 0.269155 | 0.273316 | 0.041596 |
| 0.4 | 0.048814 | 0.912767 | 0.038419 | 0 | 0.001488 |
| 0.4 | 0.04777 | 0.769868 | 0.182362 | 0 | 0.025439 |
| 0.4 | 0.075708 | 0.831813 | 0.092479 | 0 | 0.125231 |
| 0.4 | 0.062328 | 0.768301 | 0.146098 | 0.023273 | 0.041255 |
| 0.39 | 0.12639 | 0.853682 | 0.019928 | 0 | 0.018693 |
| 0.39 | 0.019231 | 0.918595 | 0.062174 | 0 | 0.02441 |
| 0.39 | 0.069932 | 0.770161 | 0.159907 | 0 | 0.300129 |
| 0.39 | 0.091309 | 0.876741 | 0.03195 | 0 | 0.02242 |
| 0.39 | 0.017442 | 0.520835 | 0.389462 | 0.072261 | 0.001443 |
| 0.38 | 0.020372 | 0.836787 | 0.071528 | 0.071313 | 0.01532 |
| 0.38 | 0.122952 | 0.730519 | 0.14653 | 0 | 0.089381 |
| 0.38 | 0.020504 | 0.893617 | 0.085879 | 0 | 0.028256 |
| 0.37 | 0.135385 | 0.837915 | 0.0267 | 0 | 0.059579 |
| 0.37 | 0.036346 | 0.809759 | 0.128229 | 0.025666 | 0.037496 |
| 0.36 | 0.045414 | 0.745517 | 0.195632 | 0.013437 | 0.031831 |
| 0.36 | 0.050315 | 0.598024 | 0.351513 | 0.000147 | 0.038121 |
| 0.35 | 0.038604 | 0.954512 | 0.006884 | 0 | 0.02608 |
| 0.35 | 0.032002 | 0.781137 | 0.152364 | 0.034498 | 0.000807 |
| 0.32 | 0.059391 | 0.887374 | 0.052975 | 0.00026 | 0.002722 |
| 0.32 | 0.070667 | 0.90593 | 0.02212 | 0.001283 | 0.083461 |
| 0.31 | 0.029173 | 0.847523 | 0.123304 | 0 | 0.02368 |
| 0.31 | 0.016053 | 0.877654 | 0.080995 | 0.025299 | 0.022378 |
| 0.31 | 0.182413 | 0.806462 | 0.011125 | 0 | 0.037714 |
| 0.31 | 0.042009 | 0.716236 | 0.241755 | 0 | 0.25503 |
| 0.3 | 0.070053 | 0.928604 | 0.001343 | 0 | 0.237388 |
| 0.3 | 0.055696 | 0.879582 | 0.060887 | 0.003835 | 0.021538 |
| 0.29 | 0.086829 | 0.552521 | 0.36065 | 0 | 0.123484 |
| 0.29 | 0.031111 | 0.552667 | 0.394879 | 0.021342 | 0.026238 |
| 0.28 | 0.032119 | 0.666449 | 0.28536 | 0.016073 | 0.089523 |
| 0.27 | 0.027594 | 0.755788 | 0.216618 | 0 | 0.07257 |
| 0.26 | 0.045204 | 0.596497 | 0.333565 | 0.01242 | 0.118649 |
| 0.25 | 0.014656 | 0.937846 | 0.041141 | 0.006357 | 0.017017 |
| 0.25 | 0.022281 | 0.817366 | 0.144456 | 0.015897 | 0.002968 |
| 0.24 | 0.052255 | 0.898211 | 0.046067 | 0.003468 | 0.000502 |
| 0.24 | 0.058124 | 0.890746 | 0.007969 | 0.043162 | 0.066551 |
| 0.24 | 0.02038 | 0.918323 | 0.036001 | 0.025295 | 0.032543 |
| 0.24 | 0.092467 | 0.907533 | 0 | 0 | 0.110143 |
| 0.23 | 0.11188 | 0.782154 | 0.105966 | 0 | 0.001959 |
| 0.23 | 0.036829 | 0.673528 | 0.283352 | 0.006291 | 0.031069 |
| 0.21 | 0.04377 | 0.927657 | 0.028573 | 0 | 0.115429 |
| 0.2 | 0.224025 | 0.744055 | 0.03192 | 0 | 0.080825 |
| 0.2 | 0.077048 | 0.723402 | 0.19955 | 0 | 0.077261 |
| 0.19 | 0.018798 | 0.873342 | 0.096714 | 0.011147 | 0.000615 |
| 0.17 | 0.035057 | 0.565328 | 0.393756 | 0.005859 | 0.098151 |
| 0.14 | 0.035536 | 0.632685 | 0.323556 | 0.008224 | 0.021802 |
| 0.14 | 0.028708 | 0.609643 | 0.361648 | 0 | 0.082055 |
| 0.12 | 0.041492 | 0.83554 | 0.122968 | 0 | 0.236861 |

*European polecat*

| Harvest  (ind./100ha) | SETTLEMENTS  (%) | CROPS  (%) | FORESTS  (%) | WATER  (%) | WIND FARM  (%) |
| --- | --- | --- | --- | --- | --- |
| 11.23 | 0.064454 | 0.807537 | 0.118761 | 0.009248 | 0.039809 |
| 9.58 | 0.078715 | 0.778306 | 0.133458 | 0.009521 | 0.022315 |
| 8.35 | 0.051785 | 0.901516 | 0.046699 | 0 | 0.007171 |
| 6.32 | 0.017478 | 0.896971 | 0.020908 | 0.064643 | 0.124726 |
| 5.8 | 0.096224 | 0.839476 | 0.0643 | 0 | 0.055361 |
| 5.48 | 0.012366 | 0.881566 | 0.053689 | 0.052379 | 0.104641 |
| 4.64 | 0.012828 | 0.801466 | 0.169907 | 0.015799 | 0.035618 |
| 4.4 | 0.034684 | 0.90422 | 0.061095 | 0 | 0.079926 |
| 4.12 | 0.035595 | 0.757742 | 0.206663 | 0 | 0.026593 |
| 4.07 | 0.130716 | 0.674756 | 0.194527 | 0 | 0.138547 |
| 4.07 | 0.033665 | 0.827557 | 0.095811 | 0.042966 | 0.085835 |
| 3.37 | 0.039445 | 0.880548 | 0.078509 | 0.001499 | 0.00146 |
| 3.31 | 0.03398 | 0.965065 | 0.000956 | 0 | 0.457655 |
| 3.27 | 0.02225 | 0.900537 | 0.067932 | 0.00928 | 0.000577 |
| 3.22 | 0.028434 | 0.931806 | 0.027306 | 0.012453 | 0.127255 |
| 3.16 | 0.024747 | 0.964457 | 0.010796 | 0 | 0.256071 |
| 3.11 | 0.041354 | 0.793022 | 0.109663 | 0.055961 | 0.156689 |
| 3.03 | 0.02289 | 0.955775 | 0.021335 | 0 | 0.120855 |
| 2.89 | 0.032845 | 0.784505 | 0.155122 | 0.027527 | 0.09242 |
| 2.82 | 0.027328 | 0.959282 | 0.005139 | 0.008252 | 0.023328 |
| 2.72 | 0.011822 | 0.922221 | 0.0577 | 0.008256 | 0.027789 |
| 2.71 | 0.030864 | 0.930063 | 0 | 0.039073 | 0.003036 |
| 2.64 | 0.01259 | 0.695308 | 0.274294 | 0.017807 | 0.002173 |
| 2.61 | 0.036113 | 0.84386 | 0.028627 | 0.0914 | 0.01802 |
| 2.49 | 0.024205 | 0.53313 | 0.202177 | 0.240487 | 0.002058 |
| 2.49 | 0.041708 | 0.943745 | 0 | 0.014547 | 0.008971 |
| 2.48 | 0.030564 | 0.913953 | 0.055484 | 0 | 0.087083 |
| 2.46 | 0.005309 | 0.943367 | 0.02594 | 0.025384 | 0.000813 |
| 2.44 | 0.03865 | 0.910378 | 0.050972 | 0 | 0.003528 |
| 2.42 | 0.029447 | 0.903798 | 0.066755 | 0 | 0.23376 |
| 2.41 | 0.009221 | 0.887556 | 0.073236 | 0.029987 | 0.245076 |
| 2.36 | 0.046884 | 0.923363 | 0.029753 | 0 | 0.00521 |
| 2.35 | 0.108022 | 0.691244 | 0.180332 | 0.020402 | 0.004315 |
| 2.33 | 0.031482 | 0.918601 | 0 | 0.049917 | 0.016405 |
| 2.32 | 0.031613 | 0.961818 | 0.003158 | 0.003411 | 0.046069 |
| 2.24 | 0.027304 | 0.917848 | 0.04837 | 0.006477 | 0.349861 |
| 2.23 | 0.026258 | 0.530627 | 0.436197 | 0.006918 | 0.028396 |
| 2.19 | 0.068853 | 0.901758 | 0.029389 | 0 | 0.153666 |
| 2.17 | 0.018063 | 0.899386 | 0.044394 | 0.038157 | 0.085235 |
| 2.15 | 0.06005 | 0.93995 | 0 | 0 | 0.023202 |
| 2.15 | 0.072737 | 0.703643 | 0.172948 | 0.050672 | 0.003121 |
| 2.14 | 0.039287 | 0.659343 | 0.239964 | 0.061407 | 0.021402 |
| 2.11 | 0.050098 | 0.942054 | 0.007554 | 0.000294 | 0.032512 |
| 2.08 | 0.043339 | 0.663185 | 0.278789 | 0.014687 | 0.102922 |
| 2 | 0.095858 | 0.675914 | 0.223198 | 0.005031 | 0.017825 |
| 1.99 | 0.050363 | 0.949637 | 0 | 0 | 0.000103 |
| 1.96 | 0.050548 | 0.895549 | 0.053477 | 0.000426 | 0.040513 |
| 1.95 | 0.050611 | 0.799196 | 0.130295 | 0.019897 | 0.045145 |
| 1.95 | 0.105751 | 0.72069 | 0.166077 | 0.007481 | 0.034231 |
| 1.94 | 0.048955 | 0.647511 | 0.303534 | 0 | 0.045488 |
| 1.93 | 0.036312 | 0.904341 | 0.024842 | 0.034504 | 0.160208 |
| 1.93 | 0.04551 | 0.731711 | 0.222779 | 0 | 0.007162 |
| 1.92 | 0.037255 | 0.909058 | 0.053687 | 0 | 0.110157 |
| 1.91 | 0.01712 | 0.800569 | 0.132823 | 0.049489 | 0.06699 |
| 1.88 | 0.004494 | 0.931113 | 0.025152 | 0.03924 | 0.009948 |
| 1.87 | 0.073048 | 0.7756 | 0.151352 | 0 | 0.061654 |
| 1.86 | 0.032386 | 0.701748 | 0.255853 | 0.010013 | 0.027642 |
| 1.85 | 0.054189 | 0.877665 | 0.068146 | 0 | 0.141787 |
| 1.83 | 0.04838 | 0.768229 | 0.137646 | 0.045745 | 0.000874 |
| 1.8 | 0.008458 | 0.766388 | 0.13723 | 0.087924 | 0.073515 |
| 1.8 | 0.222442 | 0.74335 | 0.034208 | 0 | 0.006338 |
| 1.79 | 0.02416 | 0.973681 | 0.00216 | 0 | 0.016484 |
| 1.79 | 0.086482 | 0.913518 | 0 | 0 | 0.005667 |
| 1.78 | 0.036539 | 0.951535 | 0.011926 | 0 | 0.025928 |
| 1.78 | 0.137039 | 0.844241 | 0.01872 | 0 | 0.054031 |
| 1.77 | 0.098187 | 0.786894 | 0.114919 | 0 | 0.033695 |
| 1.72 | 0.069289 | 0.841813 | 0.088899 | 0 | 0.074837 |
| 1.71 | 0.21499 | 0.702711 | 0.080053 | 0.002246 | 0.007246 |
| 1.7 | 0.025165 | 0.709589 | 0.265246 | 0 | 0.340679 |
| 1.69 | 0.074414 | 0.869734 | 0.019153 | 0.036699 | 0.003266 |
| 1.68 | 0.072101 | 0.86385 | 0.054567 | 0.009482 | 0.064817 |
| 1.66 | 0.036323 | 0.95832 | 0.005356 | 0 | 0.348035 |
| 1.64 | 0.039363 | 0.856503 | 0.095384 | 0.00875 | 0.023128 |
| 1.63 | 0.111642 | 0.775675 | 0.112682 | 0 | 0.047805 |
| 1.61 | 0.074589 | 0.640797 | 0.26404 | 0.020574 | 0.021459 |
| 1.61 | 0.028499 | 0.562559 | 0.360137 | 0.048805 | 0.001166 |
| 1.59 | 0.018482 | 0.571366 | 0.403494 | 0.006658 | 0.08103 |
| 1.58 | 0.030356 | 0.662937 | 0.306707 | 0 | 0.021427 |
| 1.57 | 0.18621 | 0.781401 | 0.032389 | 0 | 0.147147 |
| 1.54 | 0.010139 | 0.929534 | 0.039062 | 0.021265 | 0.02452 |
| 1.52 | 0.051852 | 0.630668 | 0.304929 | 0.01255 | 0.049537 |
| 1.51 | 0.034088 | 0.734203 | 0.231709 | 0 | 0.003313 |
| 1.5 | 0.008526 | 0.940563 | 0.050911 | 0 | 0.124726 |
| 1.5 | 0.034773 | 0.709889 | 0.247028 | 0.008309 | 0.059819 |
| 1.47 | 0.110639 | 0.740989 | 0.108626 | 0.039746 | 0.079146 |
| 1.47 | 0.074807 | 0.798245 | 0.126948 | 0 | 0.052297 |
| 1.47 | 0.023883 | 0.761086 | 0.215031 | 0 | 0.033082 |
| 1.46 | 0.032947 | 0.773658 | 0.145192 | 0.048202 | 0.049896 |
| 1.45 | 0.059836 | 0.773228 | 0.059752 | 0.107184 | 0.135669 |
| 1.44 | 0.018223 | 0.50482 | 0.425159 | 0 | 0.016896 |
| 1.43 | 0.026162 | 0.710156 | 0.240745 | 0.022937 | 0.031545 |
| 1.42 | 0.141596 | 0.638012 | 0.156134 | 0.064258 | 0.108228 |
| 1.42 | 0.029263 | 0.64246 | 0.263832 | 0.064444 | 0.068015 |
| 1.41 | 0.01082 | 0.979523 | 0 | 0.009656 | 0.210532 |
| 1.38 | 0.03505 | 0.940453 | 0 | 0.024497 | 0.000582 |
| 1.36 | 0.019528 | 0.767714 | 0.212758 | 0 | 0.030766 |
| 1.35 | 0.039863 | 0.711922 | 0.248216 | 0 | 0.020078 |
| 1.34 | 0.139056 | 0.729618 | 0.11691 | 0.014416 | 2.54E-05 |
| 1.33 | 0.014883 | 0.952852 | 0.026053 | 0.006211 | 0.039717 |
| 1.32 | 0.017027 | 0.946215 | 0.036758 | 0 | 0.005504 |
| 1.28 | 0.135385 | 0.837915 | 0.0267 | 0 | 0.059579 |
| 1.27 | 0.059469 | 0.940531 | 0 | 0 | 0.036402 |
| 1.27 | 0.080504 | 0.831155 | 0.005685 | 0.082657 | 0.041065 |
| 1.27 | 0.059243 | 0.805019 | 0.135738 | 0 | 0.094313 |
| 1.27 | 0.030802 | 0.910639 | 0.052474 | 0.006085 | 0.34264 |
| 1.26 | 0.007534 | 0.991543 | 0 | 0.000922 | 0.020689 |
| 1.25 | 0.01512 | 0.858559 | 0.07309 | 0.053231 | 0.037677 |
| 1.25 | 0.101064 | 0.634403 | 0.264178 | 0.000356 | 0.002776 |
| 1.25 | 0.046194 | 0.608006 | 0.345294 | 0.000506 | 0.002681 |
| 1.24 | 0.067538 | 0.901478 | 0.024953 | 0.00603 | 0.018914 |
| 1.21 | 0.036485 | 0.81515 | 0.148365 | 0 | 0.19155 |
| 1.2 | 0.033553 | 0.805444 | 0.155166 | 0.005836 | 0.089808 |
| 1.19 | 0.216664 | 0.779225 | 0.004111 | 0 | 0.294511 |
| 1.17 | 0.069932 | 0.770161 | 0.159907 | 0 | 0.300129 |
| 1.16 | 0.064382 | 0.647883 | 0.274131 | 0.013604 | 0.054466 |
| 1.13 | 0.014978 | 0.876519 | 0.099945 | 0.008559 | 0.01608 |
| 1.13 | 0.007743 | 0.882418 | 0.104562 | 0.005277 | 0.030295 |
| 1.1 | 0.025046 | 0.688707 | 0.286247 | 0 | 0.026522 |
| 1.1 | 0.134972 | 0.724281 | 0.120111 | 0.020636 | 0.008062 |
| 1.1 | 0.040076 | 0.833117 | 0.087333 | 0.039474 | 0.241711 |
| 1.08 | 0.009555 | 0.979002 | 0.000198 | 0.011245 | 0.076538 |
| 1.08 | 0.035856 | 0.89092 | 0.073224 | 0 | 0.152419 |
| 1.05 | 0.033275 | 0.900866 | 0.060343 | 0.005515 | 0.091831 |
| 1.04 | 0.08025 | 0.857246 | 0.062497 | 7.34E-06 | 0.108955 |
| 1.04 | 0.137764 | 0.692755 | 0.169481 | 0 | 0.01181 |
| 1.04 | 0.070047 | 0.841396 | 0.083412 | 0.005146 | 0.038956 |
| 1.04 | 0.077519 | 0.685448 | 0.237032 | 0 | 0.018202 |
| 1.02 | 0.044407 | 0.778417 | 0.176709 | 0.000467 | 0.032712 |
| 1.02 | 0.021323 | 0.624195 | 0.354482 | 0 | 0.002746 |
| 1.01 | 0.029025 | 0.753063 | 0.182242 | 0.03567 | 0.000693 |
| 1 | 0.008343 | 0.991657 | 0 | 0 | 0.039714 |
| 0.97 | 0.013546 | 0.804015 | 0.125482 | 0.056958 | 0.043361 |
| 0.95 | 0.021237 | 0.959433 | 0.019331 | 0 | 0.256702 |
| 0.94 | 0.021748 | 0.928259 | 0.049992 | 7.31E-07 | 0.043612 |
| 0.94 | 0.056379 | 0.943621 | 0 | 0 | 0.123161 |
| 0.93 | 0.041847 | 0.933975 | 0 | 0.024178 | 0.027727 |
| 0.93 | 0.029175 | 0.877794 | 0.093031 | 0 | 0.015471 |
| 0.92 | 0.069296 | 0.774471 | 0.070706 | 0.085528 | 0.031938 |
| 0.91 | 0.068708 | 0.849552 | 0.042768 | 0.038973 | 0.074069 |
| 0.91 | 0.049055 | 0.900592 | 0.050353 | 0 | 0.099 |
| 0.9 | 0.071363 | 0.916463 | 0.012174 | 0 | 0.181614 |
| 0.89 | 0.061675 | 0.89241 | 0.045559 | 0.000356 | 0.165958 |
| 0.89 | 0.071109 | 0.886488 | 0.042403 | 0 | 0.027537 |
| 0.89 | 0.045885 | 0.95365 | 0.000465 | 0 | 0.003323 |
| 0.89 | 0.022756 | 0.827933 | 0.149312 | 0 | 0.036017 |
| 0.88 | 0.083189 | 0.898119 | 0.012762 | 0.00593 | 0.16835 |
| 0.88 | 0.053152 | 0.914463 | 0.025729 | 0.006655 | 0.029585 |
| 0.87 | 0.073033 | 0.813903 | 0.097747 | 0.015317 | 0.19531 |
| 0.87 | 0.026149 | 0.925556 | 0.048294 | 0 | 0.048499 |
| 0.87 | 0.040363 | 0.948775 | 0.010863 | 0 | 0.06336 |
| 0.86 | 0.04377 | 0.927657 | 0.028573 | 0 | 0.115429 |
| 0.86 | 0.007404 | 0.887462 | 0.105134 | 0 | 0.179264 |
| 0.86 | 0.072839 | 0.741172 | 0.185989 | 0 | 0.02913 |
| 0.85 | 0.046888 | 0.885726 | 0.036393 | 0.030993 | 0.012365 |
| 0.85 | 0.092472 | 0.841024 | 0.066505 | 0 | 0.094696 |
| 0.84 | 0.016972 | 0.707628 | 0.225538 | 0.049862 | 0.111867 |
| 0.84 | 0.05913 | 0.575212 | 0.365601 | 5.72E-05 | 0.03847 |
| 0.83 | 0.066768 | 0.576755 | 0.179528 | 0.176949 | 0.046495 |
| 0.82 | 0.055552 | 0.69975 | 0.239609 | 0.005088 | 0.003034 |
| 0.82 | 0.119335 | 0.541145 | 0.336507 | 0.003013 | 0.018434 |
| 0.82 | 0.015126 | 0.759174 | 0.204304 | 0.021397 | 0.097869 |
| 0.81 | 0.046144 | 0.680903 | 0.254461 | 0.018493 | 0.000879 |
| 0.81 | 0.003095 | 0.950925 | 0.015182 | 0.030799 | 0.103433 |
| 0.81 | 0.028579 | 0.779842 | 0.184295 | 0.007283 | 0.00428 |
| 0.81 | 0.203607 | 0.526209 | 0.270184 | 0 | 0.050672 |
| 0.8 | 0.045044 | 0.886899 | 0.026934 | 0.041122 | 0.00106 |
| 0.8 | 0.029145 | 0.731754 | 0.289111 | 0 | 0.037475 |
| 0.78 | 0.038505 | 0.828164 | 0.133331 | 0 | 0.114387 |
| 0.78 | 0.028722 | 0.608216 | 0.319598 | 0.043464 | 0.002594 |
| 0.77 | 0.053881 | 0.915746 | 0.006846 | 0.023526 | 0.034166 |
| 0.77 | 0.122952 | 0.730519 | 0.14653 | 0 | 0.089381 |
| 0.77 | 0.016397 | 0.951369 | 0.032233 | 0 | 0.022951 |
| 0.77 | 0.124811 | 0.814342 | 0.060847 | 0 | 0.105066 |
| 0.76 | 0.011447 | 0.645638 | 0.323308 | 0.019607 | 0.001809 |
| 0.75 | 0.041876 | 0.634991 | 0.323132 | 0 | 0.019803 |
| 0.75 | 0.03637 | 0.8723 | 0.091329 | 0 | 0.000179 |
| 0.75 | 0.110983 | 0.870325 | 0.018692 | 0 | 0.141334 |
| 0.75 | 0.033849 | 0.683169 | 0.28066 | 0.002321 | 0.049347 |
| 0.74 | 0.023913 | 0.904383 | 0.050222 | 0.021483 | 0.365279 |
| 0.74 | 0.032367 | 0.938775 | 0.019579 | 0.009278 | 0.008665 |
| 0.73 | 0.042539 | 0.616348 | 0.341113 | 0 | 0.050441 |
| 0.73 | 0.042169 | 0.597119 | 0.360712 | 0 | 0.036652 |
| 0.73 | 0.142246 | 0.407022 | 0.377298 | 0.073434 | 0.089493 |
| 0.73 | 0.058187 | 0.528333 | 0.413479 | 0 | 0.002676 |
| 0.73 | 0.028556 | 0.832744 | 0.1387 | 0 | 0.027699 |
| 0.73 | 0.030872 | 0.686417 | 0.272097 | 0.010614 | 0.010509 |
| 0.72 | 0.02551 | 0.914172 | 0.045998 | 0.01432 | 0.02754 |
| 0.71 | 0.112847 | 0.540884 | 0.2825 | 0.063769 | 0.234756 |
| 0.71 | 0.043831 | 0.54447 | 0.298957 | 0.112741 | 0.002629 |
| 0.7 | 0.025413 | 0.787728 | 0.186858 | 0 | 0.021162 |
| 0.7 | 0.028501 | 0.93417 | 0.037329 | 0 | 7.53E-06 |
| 0.69 | 0.07399 | 0.807539 | 0.022472 | 0.096 | 0.028334 |
| 0.69 | 0.060899 | 0.775398 | 0.163702 | 0 | 0.022238 |
| 0.68 | 0.059338 | 0.879605 | 0.056493 | 0 | 0.222732 |
| 0.68 | 0.041801 | 0.944368 | 0 | 0.013831 | 0.010372 |
| 0.67 | 0.033119 | 0.862447 | 0.104434 | 0 | 0.024761 |
| 0.66 | 0.037281 | 0.903436 | 0.042729 | 0.016554 | 0.047312 |
| 0.66 | 0.032047 | 0.620483 | 0.34747 | 0 | 0.032438 |
| 0.66 | 0.11287 | 0.864337 | 0.022793 | 0 | 0.094936 |
| 0.66 | 0.097723 | 0.899411 | 0.002866 | 0 | 0.018141 |
| 0.66 | 0.017442 | 0.520835 | 0.389462 | 0.072261 | 0.001443 |
| 0.65 | 0.02633 | 0.705616 | 0.268053 | 0 | 3.04E-06 |
| 0.65 | 0.039787 | 0.928108 | 0.016795 | 0.015311 | 0.04215 |
| 0.64 | 0.021306 | 0.96547 | 0.000434 | 0.01279 | 0.093825 |
| 0.64 | 0.03957 | 0.723527 | 0.232789 | 0.004115 | 0.000244 |
| 0.64 | 0.033544 | 0.839022 | 0.127434 | 0 | 0.062529 |
| 0.63 | 0.020372 | 0.836787 | 0.071528 | 0.071313 | 0.01532 |
| 0.63 | 0.014212 | 0.940359 | 0.02911 | 0.01632 | 0.085242 |
| 0.63 | 0.015311 | 0.850012 | 0.134677 | 0 | 0.043252 |
| 0.62 | 0.016053 | 0.877654 | 0.080995 | 0.025299 | 0.022378 |
| 0.62 | 0.039215 | 0.907213 | 0.053573 | 0 | 0.070329 |
| 0.62 | 0.072848 | 0.708754 | 0.212019 | 0.006379 | 0.2346 |
| 0.62 | 0.042009 | 0.716236 | 0.241755 | 0 | 0.25503 |
| 0.61 | 0.007157 | 0.760099 | 0.232744 | 0 | 0.138899 |
| 0.61 | 0.035435 | 0.823689 | 0.105185 | 0.035691 | 0.047584 |
| 0.61 | 0.061571 | 0.728748 | 0.209681 | 0 | 0.041562 |
| 0.61 | 0.028701 | 0.880606 | 0.090693 | 0 | 0.1192 |
| 0.6 | 0.030852 | 0.937414 | 0.022909 | 0.008825 | 0.063511 |
| 0.6 | 0.020053 | 0.595764 | 0.384183 | 0 | 0.022035 |
| 0.6 | 0.043989 | 0.711189 | 0.244822 | 0 | 0.171178 |
| 0.59 | 0.029683 | 0.738291 | 0.186006 | 0.046019 | 0.017025 |
| 0.58 | 0.022698 | 0.676984 | 0.300318 | 0 | 0.086161 |
| 0.56 | 0.017755 | 0.694353 | 0.28756 | 0.000331 | 0.007311 |
| 0.56 | 0.142331 | 0.782279 | 0.058797 | 0.016592 | 0.041235 |
| 0.55 | 0.059471 | 0.75569 | 0.181554 | 0.003285 | 0.032832 |
| 0.55 | 0.023232 | 0.748449 | 0.204923 | 0.023397 | 0.029774 |
| 0.55 | 0.06371 | 0.687375 | 0.248915 | 0 | 0.024296 |
| 0.55 | 0.046235 | 0.768727 | 0.185038 | 0 | 0.003722 |
| 0.55 | 0.053764 | 0.773062 | 0.173173 | 0 | 0.151376 |
| 0.54 | 0.012941 | 0.611311 | 0.36866 | 0.007087 | 0.050402 |
| 0.53 | 0.064139 | 0.813701 | 0.122161 | 0 | 0.111806 |
| 0.53 | 0.034493 | 0.812457 | 0.153051 | 0 | 0.073334 |
| 0.53 | 0.03372 | 0.954883 | 0.011397 | 0 | 0.059214 |
| 0.53 | 0.005345 | 0.589222 | 0.392616 | 0.012817 | 0.022783 |
| 0.52 | 0.010679 | 0.952 | 0.001661 | 0.035661 | 0.004639 |
| 0.52 | 0.049416 | 0.637355 | 0.298827 | 0.014403 | 0.051553 |
| 0.52 | 0.022113 | 0.740767 | 0.237121 | 0 | 0.271627 |
| 0.51 | 0.100035 | 0.878576 | 0 | 0.021389 | 0.004507 |
| 0.51 | 0.180694 | 0.813429 | 0 | 0.005877 | 0.020059 |
| 0.51 | 0.151948 | 0.530499 | 0.120228 | 0.197325 | 0.019356 |
| 0.51 | 0.029013 | 0.883584 | 0.082962 | 0.004441 | 0.000183 |
| 0.5 | 0.025748 | 0.720481 | 0.253771 | 0 | 0.036214 |
| 0.5 | 0.040823 | 0.959177 | 0 | 0 | 0.004214 |
| 0.49 | 0.167614 | 0.459503 | 0.216179 | 0.156704 | 0.034594 |
| 0.49 | 0.01756 | 0.936756 | 0.045684 | 0 | 0.587545 |
| 0.49 | 0.067136 | 0.715851 | 0.217013 | 0 | 0.03475 |
| 0.48 | 0.016859 | 0.887013 | 0.096128 | 0 | 0.07925 |
| 0.48 | 0.025498 | 0.828399 | 0.114062 | 0.032041 | 0.078826 |
| 0.48 | 0.012081 | 0.564216 | 0.390193 | 0.03351 | 0.035279 |
| 0.47 | 0.077906 | 0.776797 | 0.145296 | 0 | 0.026626 |
| 0.47 | 0.101092 | 0.659995 | 0.223647 | 0.015266 | 0.004566 |
| 0.47 | 0.0599 | 0.70447 | 0.203372 | 0.032257 | 0.035467 |
| 0.47 | 0.016337 | 0.866699 | 0.111594 | 0.00537 | 0.013295 |
| 0.46 | 0.04742 | 0.747043 | 0.205537 | 0 | 0.027979 |
| 0.46 | 0.037805 | 0.73086 | 0.231335 | 0 | 0.156062 |
| 0.46 | 0.039 | 0.770268 | 0.167469 | 0.023263 | 0.142616 |
| 0.46 | 0.054568 | 0.700053 | 0.236636 | 0.008742 | 0.051978 |
| 0.46 | 0.027806 | 0.664197 | 0.305735 | 0.002262 | 0.027812 |
| 0.46 | 0.04601 | 0.785236 | 0.14245 | 0.026304 | 0.131842 |
| 0.45 | 0.002975 | 0.947564 | 0.037218 | 0.012244 | 0.071134 |
| 0.45 | 0.065773 | 0.829725 | 0.103836 | 0.000666 | 0.181817 |
| 0.45 | 0.072476 | 0.909047 | 0.018477 | 0 | 0.060021 |
| 0.44 | 0.053113 | 0.649051 | 0.261498 | 0.036338 | 0.042373 |
| 0.43 | 0.069142 | 0.772286 | 0.14675 | 0.011822 | 0.039618 |
| 0.43 | 0.027512 | 0.92664 | 0.024607 | 0.02124 | 0.166807 |
| 0.43 | 0.058012 | 0.561444 | 0.380544 | 0 | 0.028502 |
| 0.43 | 0.102823 | 0.881072 | 0.016105 | 0 | 0.048898 |
| 0.42 | 0.035474 | 0.794716 | 0.16981 | 0 | 0.222865 |
| 0.42 | 0.035057 | 0.565328 | 0.393756 | 0.005859 | 0.098151 |
| 0.41 | 0.058289 | 0.8764 | 0.065311 | 0 | 0.202343 |
| 0.41 | 0.072147 | 0.370505 | 0.269155 | 0.273316 | 0.041596 |
| 0.4 | 0.048814 | 0.912767 | 0.038419 | 0 | 0.001488 |
| 0.4 | 0.224025 | 0.744055 | 0.03192 | 0 | 0.080825 |
| 0.4 | 0.036625 | 0.770464 | 0.167258 | 0.025652 | 0.178928 |
| 0.39 | 0.021546 | 0.785222 | 0.093462 | 0.09977 | 0.001934 |
| 0.39 | 0.091309 | 0.876741 | 0.03195 | 0 | 0.02242 |
| 0.38 | 0.01264 | 0.937165 | 0.043062 | 0.007133 | 0.185625 |
| 0.38 | 0.025069 | 0.954991 | 0.01994 | 0 | 0.029211 |
| 0.37 | 0.022281 | 0.817366 | 0.144456 | 0.015897 | 0.002968 |
| 0.37 | 0.103313 | 0.732831 | 0.163856 | 0 | 0.045637 |
| 0.37 | 0.077374 | 0.85315 | 0.00706 | 0.062416 | 0.027737 |
| 0.37 | 0.039739 | 0.691329 | 0.268932 | 0 | 0.195716 |
| 0.36 | 0.043956 | 0.854424 | 0.090371 | 0.011248 | 0.042874 |
| 0.36 | 0.012158 | 0.877799 | 0.104953 | 0.00509 | 0.119091 |
| 0.36 | 0.030907 | 0.705282 | 0.26381 | 0 | 0.210419 |
| 0.35 | 0.041492 | 0.83554 | 0.122968 | 0 | 0.236861 |
| 0.35 | 0.018303 | 0.841083 | 0.134267 | 0.006347 | 0.007471 |
| 0.34 | 0.029925 | 0.788213 | 0.181862 | 0 | 0.020835 |
| 0.33 | 0.139615 | 0.679458 | 0.17389 | 0.007037 | 0.000136 |
| 0.33 | 0.076638 | 0.893076 | 0.030286 | 0 | 0.003956 |
| 0.33 | 0.04557 | 0.58709 | 0.36734 | 0 | 0.070527 |
| 0.32 | 0.070103 | 0.80001 | 0.098141 | 0.031745 | 0.019618 |
| 0.32 | 0.096072 | 0.863331 | 0.040596 | 0 | 0.08757 |
| 0.32 | 0.027087 | 0.892398 | 0.06958 | 0.010935 | 0.015594 |
| 0.32 | 0.070667 | 0.90593 | 0.02212 | 0.001283 | 0.083461 |
| 0.31 | 0.029173 | 0.847523 | 0.123304 | 0 | 0.02368 |
| 0.31 | 0.182413 | 0.806462 | 0.011125 | 0 | 0.037714 |
| 0.3 | 0.019964 | 0.944058 | 0.034331 | 0.001647 | 0.054066 |
| 0.3 | 0.08957 | 0.6941 | 0.21633 | 0 | 0.055495 |
| 0.3 | 0.032369 | 0.54685 | 0.415187 | 0.005593 | 0.023739 |
| 0.3 | 0.021547 | 0.897054 | 0.081399 | 0 | 0.003304 |
| 0.3 | 0.055696 | 0.879582 | 0.060887 | 0.003835 | 0.021538 |
| 0.29 | 0.050745 | 0.719471 | 0.229783 | 0 | 0.077093 |
| 0.29 | 0.086829 | 0.552521 | 0.36065 | 0 | 0.123484 |
| 0.29 | 0.031111 | 0.552667 | 0.394879 | 0.021342 | 0.026238 |
| 0.28 | 0.072977 | 0.856731 | 0.070292 | 0 | 0.074098 |
| 0.27 | 0.027594 | 0.755788 | 0.216618 | 0 | 0.07257 |
| 0.27 | 0.029603 | 0.751571 | 0.218827 | 0 | 0.079736 |
| 0.27 | 0.059068 | 0.905269 | 0.035663 | 0 | 0.061176 |
| 0.27 | 0.031001 | 0.661591 | 0.277932 | 0.029476 | 0.000647 |
| 0.26 | 0.039841 | 0.816561 | 0.143598 | 0 | 0.101836 |
| 0.25 | 0.020569 | 0.771368 | 0.155748 | 0.052315 | 0.015713 |
| 0.25 | 0.052039 | 0.626445 | 0.321516 | 0 | 0.038994 |
| 0.25 | 0.038 | 0.931754 | 0 | 0.030246 | 7.47E-05 |
| 0.25 | 0.032981 | 0.830037 | 0.110184 | 0.026798 | 0.145802 |
| 0.24 | 0.086936 | 0.888209 | 0.024855 | 0 | 0.080122 |
| 0.24 | 0.058124 | 0.890746 | 0.007969 | 0.043162 | 0.066551 |
| 0.24 | 0.02038 | 0.918323 | 0.036001 | 0.025295 | 0.032543 |
| 0.23 | 0.027274 | 0.638686 | 0.329299 | 0.004741 | 0.087119 |
| 0.23 | 0.108214 | 0.736279 | 0.126343 | 0.029164 | 0.000839 |
| 0.22 | 0.004923 | 0.757164 | 0.144813 | 0.0931 | 0.011441 |
| 0.22 | 0.059954 | 0.694479 | 0.245567 | 0 | 0.029688 |
| 0.21 | 0.108231 | 0.891769 | 0 | 0 | 0.081012 |
| 0.21 | 0.077189 | 0.793972 | 0.122556 | 0.006282 | 0.067732 |
| 0.2 | 0.02837 | 0.674304 | 0.282227 | 0.015099 | 0.162311 |
| 0.2 | 0.077048 | 0.723402 | 0.19955 | 0 | 0.077261 |
| 0.18 | 0.034667 | 0.836308 | 0.12772 | 0.001305 | 0.026518 |
| 0.17 | 0.143477 | 0.843699 | 0.006261 | 0.006563 | 0.063003 |
| 0.17 | 0.032119 | 0.666449 | 0.28536 | 0.016073 | 0.089523 |
| 0.15 | 0.032721 | 0.728697 | 0.238522 | 5.97E-05 | 0.018052 |
| 0.14 | 0.035536 | 0.632685 | 0.323556 | 0.008224 | 0.021802 |
| 0.13 | 0.019231 | 0.918595 | 0.062174 | 0 | 0.02441 |
| 0.12 | 0.052255 | 0.898211 | 0.046067 | 0.003468 | 0.000502 |
| 0.12 | 0.014656 | 0.937846 | 0.041141 | 0.006357 | 0.017017 |
| 0.12 | 0.032002 | 0.781137 | 0.152364 | 0.034498 | 0.000807 |

*European hare*

| Harvest  (ind./100ha) | SETTLEMENTS  (%) | CROPS  (%) | FORESTS  (%) | WATER  (%) | WIND FARM  (%) |
| --- | --- | --- | --- | --- | --- |
| 22.53 | 0.027328 | 0.959282 | 0.005139 | 0.008252 | 0.023328 |
| 22.26 | 0.026939 | 0.973061 | 0 | 0 | 0.021787 |
| 20.87 | 0.18621 | 0.781401 | 0.032389 | 0 | 0.147147 |
| 19.13 | 0.011426 | 0.976589 | 0.011985 | 0 | 0.007056 |
| 17.55 | 0.017027 | 0.946215 | 0.036758 | 0 | 0.005504 |
| 16.96 | 0.092472 | 0.841024 | 0.066505 | 0 | 0.094696 |
| 14.14 | 0.02289 | 0.955775 | 0.021335 | 0 | 0.120855 |
| 13.93 | 0.028501 | 0.93417 | 0.037329 | 0 | 7.53E-06 |
| 12.76 | 0.096072 | 0.863331 | 0.040596 | 0 | 0.08757 |
| 12.55 | 0.02551 | 0.914172 | 0.045998 | 0.01432 | 0.02754 |
| 12.17 | 0.055552 | 0.69975 | 0.239609 | 0.005088 | 0.003034 |
| 12.08 | 0.048814 | 0.912767 | 0.038419 | 0 | 0.001488 |
| 11.22 | 0.039445 | 0.880548 | 0.078509 | 0.001499 | 0.00146 |
| 11.15 | 0 | 0.891838 | 0.108162 | 0 | 0.002346 |
| 11.07 | 0.005345 | 0.589222 | 0.392616 | 0.012817 | 0.022783 |
| 10.96 | 0.025046 | 0.688707 | 0.286247 | 0 | 0.026522 |
| 10.53 | 0.017478 | 0.896971 | 0.020908 | 0.064643 | 0.124726 |
| 10.18 | 0.007743 | 0.882418 | 0.104562 | 0.005277 | 0.030295 |
| 9.83 | 0.046235 | 0.768727 | 0.185038 | 0 | 0.003722 |
| 9.58 | 0.122952 | 0.730519 | 0.14653 | 0 | 0.089381 |
| 9.39 | 0.056379 | 0.943621 | 0 | 0 | 0.123161 |
| 8.51 | 0.020885 | 0.97362 | 0.005495 | 0 | 0.031392 |
| 8.37 | 0.033275 | 0.900866 | 0.060343 | 0.005515 | 0.091831 |
| 8.28 | 0.012878 | 0.947298 | 0.03675 | 0.003074 | 0.028568 |
| 8.22 | 0.039363 | 0.856503 | 0.095384 | 0.00875 | 0.023128 |
| 7.63 | 0.059469 | 0.940531 | 0 | 0 | 0.036402 |
| 7.61 | 0.012366 | 0.881566 | 0.053689 | 0.052379 | 0.104641 |
| 7.49 | 0.022281 | 0.817366 | 0.144456 | 0.015897 | 0.002968 |
| 7.41 | 0.054189 | 0.877665 | 0.068146 | 0 | 0.141787 |
| 7.38 | 0.114896 | 0.770582 | 0.111487 | 0.003035 | 0.057439 |
| 7.21 | 0.222442 | 0.74335 | 0.034208 | 0 | 0.006338 |
| 7.16 | 0.071363 | 0.916463 | 0.012174 | 0 | 0.181614 |
| 7.08 | 0.046884 | 0.923363 | 0.029753 | 0 | 0.00521 |
| 7.05 | 0.01082 | 0.979523 | 0 | 0.009656 | 0.210532 |
| 6.9 | 0.060899 | 0.775398 | 0.163702 | 0 | 0.022238 |
| 6.89 | 0.021546 | 0.785222 | 0.093462 | 0.09977 | 0.001934 |
| 6.66 | 0.004923 | 0.757164 | 0.144813 | 0.0931 | 0.011441 |
| 6.55 | 0.063612 | 0.891342 | 0.045046 | 0 | 0.107764 |
| 6.48 | 0.069142 | 0.772286 | 0.14675 | 0.011822 | 0.039618 |
| 6.04 | 0.029025 | 0.753063 | 0.182242 | 0.03567 | 0.000693 |
| 5.83 | 0.026149 | 0.925556 | 0.048294 | 0 | 0.048499 |
| 5.79 | 0.036312 | 0.904341 | 0.024842 | 0.034504 | 0.160208 |
| 5.74 | 0.017507 | 0.974532 | 0.00609 | 0.001872 | 0.18464 |
| 5.72 | 0.020504 | 0.893617 | 0.085879 | 0 | 0.028256 |
| 5.46 | 0.05676 | 0.713201 | 0.23004 | 0 | 0.025905 |
| 5.41 | 0.019964 | 0.944058 | 0.034331 | 0.001647 | 0.054066 |
| 5.37 | 0.04377 | 0.927657 | 0.028573 | 0 | 0.115429 |
| 5.36 | 0.058012 | 0.561444 | 0.380544 | 0 | 0.028502 |
| 5.34 | 0.025069 | 0.954991 | 0.01994 | 0 | 0.029211 |
| 5.31 | 0.045044 | 0.886899 | 0.026934 | 0.041122 | 0.00106 |
| 5.22 | 0.070047 | 0.841396 | 0.083412 | 0.005146 | 0.038956 |
| 5.11 | 0.046888 | 0.885726 | 0.036393 | 0.030993 | 0.012365 |
| 5.01 | 0.035743 | 0.671162 | 0.284171 | 0.008924 | 0.015164 |
| 5.01 | 0.050745 | 0.719471 | 0.229783 | 0 | 0.077093 |
| 5.01 | 0.016972 | 0.707628 | 0.225538 | 0.049862 | 0.111867 |
| 4.93 | 0.097723 | 0.899411 | 0.002866 | 0 | 0.018141 |
| 4.88 | 0.033665 | 0.827557 | 0.095811 | 0.042966 | 0.085835 |
| 4.88 | 0.01216 | 0.758907 | 0.228932 | 0 | 0.023113 |
| 4.77 | 0.092467 | 0.907533 | 0 | 0 | 0.110143 |
| 4.69 | 0.034103 | 0.818442 | 0.147454 | 0 | 0.087954 |
| 4.62 | 0.029173 | 0.847523 | 0.123304 | 0 | 0.02368 |
| 4.59 | 0.11188 | 0.782154 | 0.105966 | 0 | 0.001959 |
| 4.5 | 0.055696 | 0.879582 | 0.060887 | 0.003835 | 0.021538 |
| 4.44 | 0.023913 | 0.904383 | 0.050222 | 0.021483 | 0.365279 |
| 4.44 | 0.21499 | 0.702711 | 0.080053 | 0.002246 | 0.007246 |
| 4.41 | 0.074807 | 0.798245 | 0.126948 | 0 | 0.052297 |
| 4.35 | 0.077451 | 0.901627 | 0.020923 | 0 | 0.037246 |
| 4.35 | 0.045738 | 0.828349 | 0.125913 | 0 | 0.022493 |
| 4.24 | 0.108231 | 0.891769 | 0 | 0 | 0.081012 |
| 4.14 | 0.041354 | 0.793022 | 0.109663 | 0.055961 | 0.156689 |
| 4.11 | 0.051831 | 0.828658 | 0.11951 | 0 | 0.107904 |
| 3.97 | 0.03309 | 0.827144 | 0.139765 | 0 | 0.030663 |
| 3.96 | 0.03372 | 0.954883 | 0.011397 | 0 | 0.059214 |
| 3.92 | 0.02225 | 0.900537 | 0.067932 | 0.00928 | 0.000577 |
| 3.91 | 0.010679 | 0.952 | 0.001661 | 0.035661 | 0.004639 |
| 3.83 | 0.033544 | 0.839022 | 0.127434 | 0 | 0.062529 |
| 3.77 | 0.044084 | 0.893109 | 0.062806 | 0 | 0.010772 |
| 3.76 | 0.008343 | 0.991657 | 0 | 0 | 0.039714 |
| 3.74 | 0.033849 | 0.683169 | 0.28066 | 0.002321 | 0.049347 |
| 3.7 | 0.063263 | 0.772515 | 0.155506 | 0.008716 | 0.17232 |
| 3.66 | 0.027806 | 0.664197 | 0.305735 | 0.002262 | 0.027812 |
| 3.6 | 0.008526 | 0.940563 | 0.050911 | 0 | 0.124726 |
| 3.58 | 0.032421 | 0.811665 | 0.155915 | 0 | 0.079009 |
| 3.52 | 0.029683 | 0.738291 | 0.186006 | 0.046019 | 0.017025 |
| 3.51 | 0.095985 | 0.700639 | 0.198975 | 0.0044 | 0.02055 |
| 3.48 | 0.031613 | 0.961818 | 0.003158 | 0.003411 | 0.046069 |
| 3.44 | 0.059243 | 0.805019 | 0.135738 | 0 | 0.094313 |
| 3.35 | 0.040323 | 0.76684 | 0.192836 | 0 | 0.016819 |
| 3.33 | 0.022977 | 0.95925 | 0.006649 | 0.011124 | 0.025333 |
| 3.3 | 0.059471 | 0.75569 | 0.181554 | 0.003285 | 0.032832 |
| 3.28 | 0.101092 | 0.659995 | 0.223647 | 0.015266 | 0.004566 |
| 3.28 | 0.032047 | 0.620483 | 0.34747 | 0 | 0.032438 |
| 3.27 | 0.023232 | 0.748449 | 0.204923 | 0.023397 | 0.029774 |
| 3.19 | 0.064139 | 0.813701 | 0.122161 | 0 | 0.111806 |
| 3.19 | 0.029145 | 0.731754 | 0.289111 | 0 | 0.037475 |
| 3.13 | 0.021748 | 0.928259 | 0.049992 | 7.31E-07 | 0.043612 |
| 3.12 | 0.016053 | 0.877654 | 0.080995 | 0.025299 | 0.022378 |
| 3.1 | 0.042539 | 0.616348 | 0.341113 | 0 | 0.050441 |
| 3.07 | 0.010139 | 0.929534 | 0.039062 | 0.021265 | 0.02452 |
| 3.06 | 0.016397 | 0.951369 | 0.032233 | 0 | 0.022951 |
| 3.05 | 0.080689 | 0.873851 | 0.041035 | 0.004425 | 0.180591 |
| 3.04 | 0.058289 | 0.8764 | 0.065311 | 0 | 0.202343 |
| 3.02 | 0.01264 | 0.937165 | 0.043062 | 0.007133 | 0.185625 |
| 3.02 | 0.279126 | 0.620004 | 0.100869 | 0 | 0.032547 |
| 2.99 | 0.07399 | 0.807539 | 0.022472 | 0.096 | 0.028334 |
| 2.95 | 0.08957 | 0.6941 | 0.21633 | 0 | 0.055495 |
| 2.92 | 0.032947 | 0.773658 | 0.145192 | 0.048202 | 0.049896 |
| 2.89 | 0.035536 | 0.632685 | 0.323556 | 0.008224 | 0.021802 |
| 2.83 | 0.141596 | 0.638012 | 0.156134 | 0.064258 | 0.108228 |
| 2.81 | 0.025413 | 0.787728 | 0.186858 | 0 | 0.021162 |
| 2.75 | 0.040779 | 0.952662 | 0 | 0.006559 | 0.231493 |
| 2.66 | 0.014883 | 0.952852 | 0.026053 | 0.006211 | 0.039717 |
| 2.65 | 0.055185 | 0.873829 | 0.070986 | 0 | 0.060068 |
| 2.56 | 0.078715 | 0.778306 | 0.133458 | 0.009521 | 0.022315 |
| 2.49 | 0.041745 | 0.712863 | 0.243395 | 0.001997 | 0.02306 |
| 2.47 | 0.056828 | 0.90978 | 0.014798 | 0.018594 | 0.01458 |
| 2.44 | 0.02038 | 0.918323 | 0.036001 | 0.025295 | 0.032543 |
| 2.42 | 0.028434 | 0.931806 | 0.027306 | 0.012453 | 0.127255 |
| 2.38 | 0.058124 | 0.890746 | 0.007969 | 0.043162 | 0.066551 |
| 2.38 | 0.137039 | 0.844241 | 0.01872 | 0 | 0.054031 |
| 2.36 | 0.01221 | 0.707236 | 0.280555 | 0 | 0.010554 |
| 2.35 | 0.0599 | 0.70447 | 0.203372 | 0.032257 | 0.035467 |
| 2.24 | 0.02416 | 0.973681 | 0.00216 | 0 | 0.016484 |
| 2.17 | 0.018063 | 0.899386 | 0.044394 | 0.038157 | 0.085235 |
| 2.11 | 0.012567 | 0.970659 | 0.016774 | 0 | 0.011678 |
| 2.06 | 0.035595 | 0.757742 | 0.206663 | 0 | 0.026593 |
| 1.97 | 0.019231 | 0.918595 | 0.062174 | 0 | 0.02441 |
| 1.97 | 0.067705 | 0.60951 | 0.322784 | 0 | 0.031049 |
| 1.95 | 0.009555 | 0.979002 | 0.000198 | 0.011245 | 0.076538 |
| 1.95 | 0.042169 | 0.597119 | 0.360712 | 0 | 0.036652 |
| 1.91 | 0.053881 | 0.915746 | 0.006846 | 0.023526 | 0.034166 |
| 1.89 | 0.052255 | 0.898211 | 0.046067 | 0.003468 | 0.000502 |
| 1.89 | 0.014212 | 0.940359 | 0.02911 | 0.01632 | 0.085242 |
| 1.87 | 0.077374 | 0.85315 | 0.00706 | 0.062416 | 0.027737 |
| 1.85 | 0.069296 | 0.774471 | 0.070706 | 0.085528 | 0.031938 |
| 1.8 | 0.099347 | 0.900653 | 0 | 0 | 0.147427 |
| 1.75 | 0.014656 | 0.937846 | 0.041141 | 0.006357 | 0.017017 |
| 1.73 | 0.143477 | 0.843699 | 0.006261 | 0.006563 | 0.063003 |
| 1.67 | 0.010983 | 0.903515 | 0.085502 | 0 | 0.036375 |
| 1.63 | 0.046144 | 0.680903 | 0.254461 | 0.018493 | 0.000879 |
| 1.57 | 0.157547 | 0.729757 | 0.10907 | 0.003625 | 0.027969 |
| 1.4 | 0.064454 | 0.807537 | 0.118761 | 0.009248 | 0.039809 |
| 1.36 | 0.011822 | 0.922221 | 0.0577 | 0.008256 | 0.027789 |
| 1.36 | 0.011622 | 0.828127 | 0.160251 | 0 | 0.020163 |
| 1.36 | 0.019528 | 0.767714 | 0.212758 | 0 | 0.030766 |
| 1.23 | 0.041239 | 0.678391 | 0.280371 | 0 | 0.166558 |
| 1.13 | 0.096224 | 0.839476 | 0.0643 | 0 | 0.055361 |
| 0.97 | 0.041854 | 0.859523 | 0.094217 | 0.004407 | 0.152709 |
| 0.81 | 0.049856 | 0.810036 | 0.140108 | 0 | 0.019612 |
| 0.71 | 0.031111 | 0.552667 | 0.394879 | 0.021342 | 0.026238 |
| 0.6 | 0.046376 | 0.702486 | 0.251138 | 0 | 0.003461 |
| 0.56 | 0.009634 | 0.907754 | 0.082612 | 0 | 0.016301 |
| 0.52 | 0.01512 | 0.858559 | 0.07309 | 0.053231 | 0.037677 |
| 0.38 | 0.080504 | 0.831155 | 0.005685 | 0.082657 | 0.041065 |
