# Supplementary material for: The contingent impact of wind farms on game mammal density demonstrated in a large-scale analysis of hunting bag data in Poland
Source: Sci Rep. 2024 Oct 25;14:25290. doi: 10.1038/s41598-024-76999-2 (PMC11511958; doi:10.1038/s41598-024-76999-2)
Supplement: Supplementary file 2 — Supplementary Material 2 [file 41598_2024_76999_MOESM2_ESM.docx]

Table S2. Relation of hunting bags and estimated animal density (DENSITY) for all seven studied species with a generalized linear mixed model with gamma distribution and log link function.

| **Source** | ***B-coefficient*** | ***Standard Error*** | ***T-test*** | ***p-value*** |
| --- | --- | --- | --- | --- |
| ***Roe deer (marginal R^2^=0.134, conditional R^2^=0.603)*** | | | | |
| Intercept | 1.582 | 0.358 | 4.420 | <0.001 |
| DENSITY | 0.015 | 0.001 | 10.975 | <0.001 |
| ***Wild boar (marginal R^2^=0.106, conditional R^2^=0.631)*** | | | | |
| Intercept | 1.271 | 0.642 | 1.980 | 0.048 |
| DENSITY | 0.159 | 0.016 | 9.744 | <0.001 |
| ***Red fox (marginal R^2^=0.088, conditional R^2^=0.485)*** | | | | |
| Intercept | 1.793 | 0.415 | 4.326 | <0.001 |
| DENSITY | 0.055 | 0.006 | 9.494 | <0.001 |
| ***Raccoon dog (marginal R^2^=0.247, conditional R^2^=0.531)*** | | | | |
| Intercept | 0.015 | 0.478 | 0.032 | 0.974 |
| DENSITY | 0.291 | 0.59 | 16.046 | <0.001 |
| ***European badger (marginal R^2^=0.147, conditional R^2^=0.427)*** | | | | |
| Intercept | -0.407 | 0.446 | -0.911 | 0.363 |
| DENSITY | 0.269 | 0.023 | 11.594 | <0.001 |
| ***European polecat (marginal R^2^=0.124, conditional R^2^=0.331)*** | | | | |
| Intercept | -0.389 | 0.534 | -0.728 | 0.467 |
| DENSITY | 0.238 | 0.026 | 9.216 | <0.001 |
| ***European hare (marginal R^2^=0.110, conditional R^2^=0.466)*** | | | | |
| Intercept | 0.731 | 0.508 | 1.438 | 0.152 |
| DENSITY | 0.011 | 0.002 | 6.007 | <0.001 |
