# Supplementary material for: The contingent impact of wind farms on game mammal density demonstrated in a large-scale analysis of hunting bag data in Poland
Source: Sci Rep. 2024 Oct 25;14:25290. doi: 10.1038/s41598-024-76999-2 (PMC11511958; doi:10.1038/s41598-024-76999-2)
Supplement: Supplementary file 3 — Supplementary Material 3 [file 41598_2024_76999_MOESM3_ESM.docx]

Table S3. Ranking of models for each studied species (included only models within *ΔAICc* < 2 and null models) predicting the density of each animal within 95% confidence intervals (*∑ωi* = 0.95) (*ΔAICc* - *AICc* differences, ωi - Akaike weights, Rank - rank of the models based on *AICc* values); variables: see methods).

| **ID** | ***MODEL*** | ***ΔAICc*** | ***ω_i_*** | ***Rank*** |
| --- | --- | --- | --- | --- |
| ***Roe deer*** | | | | |
| R15 | FORESTS + WIND FARM | 0.00 | 0.11 | 1 |
| R24 | CROPS + FORESTS + WIND FARM | 0.33 | 0.09 | 2 |
| R21 | SETTLEMENTS + FORESTS + WIND FARM | 0.84 | 0.07 | 3 |
| R26 | FORESTS + WATER + WIND FARM | 1.37 | 0.06 | 4 |
| R6 | WIND FARM | 1.40 | 0.06 | 5 |
| R19 | SETTLEMENTS + CROPS + WIND FARM | 1.66 | 0.05 | 6 |
| R10 | SETTLEMENTS + WIND FARM | 1.74 | 0.05 | 7 |
| R1 | *Null model* | 2.71 | 0.03 | 15 |
| ***Wild boar*** | | | | |
| W8 | SETTLEMENTS + FORESTS | 0.00 | 0.16 | 1 |
| W12 | SETTLEMENTS + WATER | 0.63 | 0.12 | 2 |
| W17 | SETTLEMENTS + CROPS + FORESTS | 1.23 | 0.09 | 3 |
| W20 | SETTLEMENTS + FORESTS + WATER | 1.29 | 0.08 | 4 |
| W18 | SETTLEMENTS + CROPS + WATER | 1.38 | 0.08 | 5 |
| W21 | SETTLEMENTS + FORESTS + WIND FARM | 1.42 | 0.08 | 6 |
| W23 | CROPS + FORESTS + WATER | 1.45 | 0.08 | 7 |
| W1 | *Null model* | 16.25 | 0.00 | 25 |
| ***Red fox*** | | | | |
| F3 | CROPS | 0.00 | 0.12 | 1 |
| F4 | FORESTS | 0.35 | 0.10 | 2 |
| F14 | FORESTS + WATER | 0.85 | 0.08 | 3 |
| F7 | SETTLEMENTS + CROPS | 1.39 | 0.06 | 4 |
| F12 | CROPS + WATER | 1.49 | 0.06 | 5 |
| F11 | CROPS + FORESTS | 1.93 | 0.05 | 6 |
| F1 | *Null model* | 2.10 | 0.04 | 8 |
| ***Raccoon dog*** | | | | |
| D3 | CROPS | 0.00 | 0.11 | 1 |
| D1 | *Null model* | 0.59 | 0.08 | 2 |
| D4 | FORESTS | 0.87 | 0.07 | 3 |
| D5 | WATER | 1.18 | 0.06 | 4 |
| D13 | CROPS + WIND FARM | 1.29 | 0.06 | 5 |
| D14 | FORESTS + WATER | 1.66 | 0.05 | 6 |
| D12 | CROPS + WATER | 1.72 | 0.05 | 7 |
| D11 | CROPS + FORESTS | 1.99 | 0.04 | 8 |
| ***European badger*** | | | | |
| B1 | *Null model* | 0.00 | 0.19 | 1 |
| B5 | WATER | 1.59 | 0.08 | 2 |
| B6 | WIND FARM | 1.71 | 0.08 | 3 |
| B2 | SETTLEMENTS | 1.90 | 0.07 | 4 |
| ***European polecat*** | | | | |
| P12 | CROPS + WATER | 0.00 | 0.13 | 1 |
| P4 | FORESTS | 0.02 | 0.13 | 2 |
| P14 | FORESTS + WATER | 0.87 | 0.08 | 3 |
| P8 | SETTLEMENTS + FORESTS | 1.21 | 0.07 | 4 |
| P3 | CROPS | 1.65 | 0.06 | 5 |
| P17 | SETTLEMENTS + CROPS + FORESTS | 1.85 | 0.05 | 6 |
| P20 | SETTLEMENTS + FORESTS + WATER | 1.96 | 0.05 | 7 |
| P1 | *Null model* | 6.28 | 0.01 | 23 |
| ***European hare*** | | | | |
| H3 | CROPS | 0.00 | 0.21 | 1 |
| H8 | SETTLEMENTS + FORESTS | 1.63 | 0.09 | 2 |
| H12 | CROPS + WATER | 1.65 | 0.09 | 3 |
| H1 | *Null model* | 13.13 | 0.00 | 24 |
