# Supplementary material for: The contingent impact of wind farms on game mammal density demonstrated in a large-scale analysis of hunting bag data in Poland
Source: Sci Rep. 2024 Oct 25;14:25290. doi: 10.1038/s41598-024-76999-2 (PMC11511958; doi:10.1038/s41598-024-76999-2)
Supplement: Supplementary file 4 — Supplementary Material 4 [file 41598_2024_76999_MOESM4_ESM.docx]

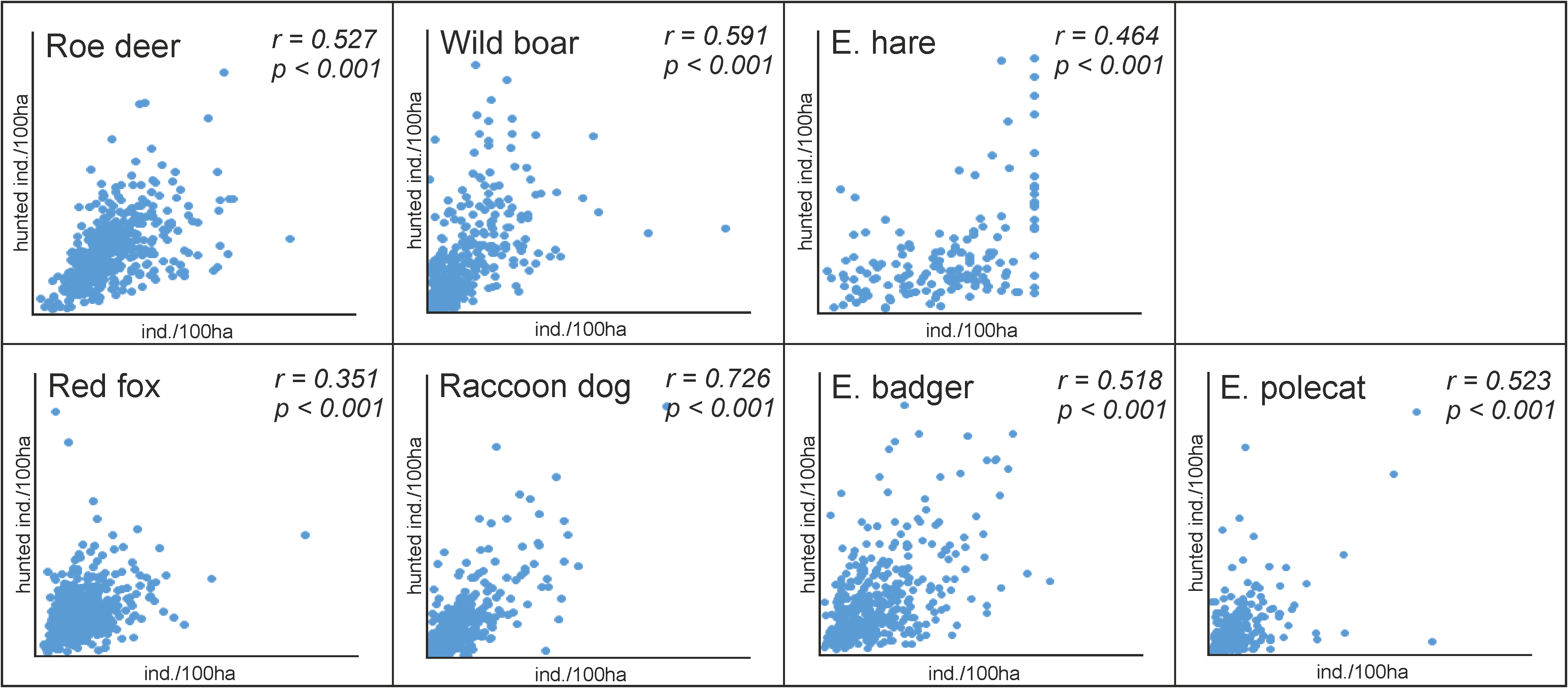


Figure S1. Graphical presentation of correlation between hunting bugs and estimated animal density for seven studied game mammals.
